# Molecular Engineering for Nonlinear Fluorescence: En Route to Three-Photon Absorption via Sequential One-Photon Excitation

**DOI:** 10.1021/jacs.6c03621

**Published:** 2026-05-01

**Authors:** Jinyoung Oh, Carlos Benitez-Martin, Eduard Fron, Johan Hofkens, Uwe Pischel, Morten Grøtli, Joakim Andréasson

**Affiliations:** † Department of Chemistry and Molecular Biology, 3570 University of Gothenburg, SE-41296 Göteborg, Sweden; ‡ Chemistry and Chemical Engineering, Chemistry and Biochemistry, Chalmers University of Technology, SE-41296 Göteborg, Sweden; § Department of Chemistry, 54517 KU Leuven, Celestijnenlaan 200F, B-3001 Leuven, Belgium; ∥ Core Facility for Advanced Spectroscopy, KU Leuven, Celestijnenlaan 200F, B-3001 Leuven, Belgium; ⊥ Max Planck Institute for Polymer Research, Ackermannweg 10, 55128 Mainz, Germany; # CIQSO—Center for Research in Sustainable Chemistry and Department of Chemistry, University of Huelva, Campus de El Carmen s/n, E-21071 Huelva, Spain

## Abstract

Multiphoton excitation (MPE) processes enable three-dimensionally confined fluorescence with reduced background as well as improved image contrast and signal-to-noise ratio. However, MPE finds major technological limitations derived from the need for high light intensities at long excitation wavelengths. To circumvent these challenges, we herein propose a molecular strategy that reproduces multiphoton-like nonlinear responses using only sequential one-photon excitations (1PE). A dyad (2for1), consisting of the acedan fluorophore covalently connected to a spironaphthopyran photoswitch, shows a quadratic dependence of the emission intensity on the excitation intensity, thus emulating a two-photon absorption behavior. The incorporation of a BODIPY photocage to this construct yields a triad (3for1) that results in an even stronger nonlinear fluorescence response. These findings pave the way for sequential 1PE as a practical approach to capitalize on the benefits of nonlinear fluorescence while avoiding the inherent limitations of simultaneous MPE.

## Graphical abstract

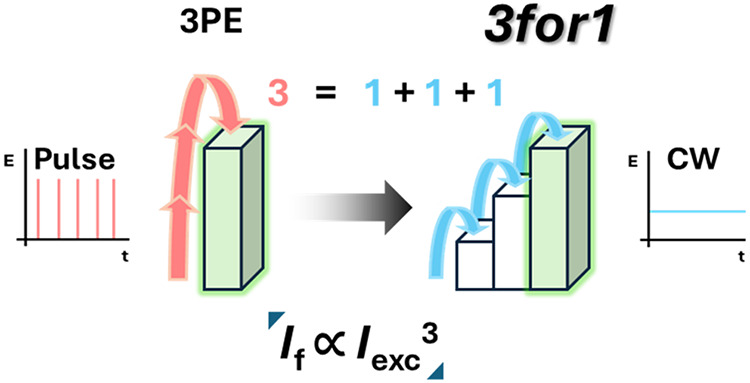

## Introduction

Fluorescence spectroscopy and microscopy have become indispensable tools across a wide variety of scientific disciplines including molecular biology, nanotechnology, and materials chemistry.^1–8^ Traditionally, excitation of fluorescent molecules has relied on one-photon absorption, where a single photon promotes the molecule from the ground state to an electronically excited state. Multiphoton excitation (MPE) offers a powerful alternative to conventional one-photon absorption, with spatial confinement as its most distinctive feature.^9^ In MPE, two (2PE), three (3PE), or even more photons of lower energy are absorbed simultaneously to trigger excitation.^10,11^ As the probability of simultaneous multiphoton absorption is proportional to the square or the cube of the excitation light intensity for 2PE^12,13^ and 3PE,^14,15^ respectively, excitation is effectively restricted to the focal volume of, for example, the microscope. This results in a substantial reduction in out-of-focus fluorescence and background signal, as well as in improved image contrast and signal-to-noise ratio. Thus, the emitted photons can be more precisely localized even within complex and/or autofluorescent environments.^16–21^

However, MPE comes with certain limitations. One of the main challenges is the requirement for higher excitation intensities to prompt simultaneous multiphoton absorption, as the required excitation intensity increases exponentially with the number of photons involved.^22^ 2PE is still relatively widely adopted (e.g., two-photon microscopy), while the extreme excitation intensities required for 3PE or higher-order processes prevent widespread use.^23,24^ Moreover, MPE requires photons of at least twice (twice for 2PE, three times for 3PE, etc.) the wavelength compared to the corresponding fundamental one-photon excitation (1PE). Thus, 2PE and 3PE generally utilize excitation wavelengths within the near-infrared spectral range. The use of such “large photons” has a negative impact on the spatial resolution, due to the linear dependence of the resolution on the excitation wavelength.^22,25^

In this work, we bypass these limitations with an alternative 1PE strategy to achieve nonlinear fluorescence responses equivalent to those obtained when using 2PE or 3PE. Our concept builds on the use of multiple sequential 1PE processes^26,27^ for the eventual observation of nonlinear fluorescence responses from molecular constructs containing a fluorescent probe equipped with photoactivatable molecules (i.e., photoswitches and photocages). Although the underlying processes involve sequential 1PE rather than simultaneous multiphoton absorption, the overall fluorescence intensity exhibits potentiated nonlinear dependence on excitation intensity.

To embed nonlinear excitation behavior into a fundamental 1PE fluorescent probe, the probe is first conjugated to a photoswitch to constitute a molecular dyad, enabling two sequential 1PE processes: a first excitation step to trigger photoisomerization to the fluorescent isomer (activation), followed by a second excitation for fluorescence readout. This molecular design, termed 2for1, yields a quadratic dependence of the fluorescence intensity on the excitation intensity, thereby mimicking a 2PE behavior.^28^ Expanding on this scheme, the 2for1 dyad is further attached to a photocage to constitute a molecular triad that we refer to as 3for1. Following similar reasoning, a 3for1 triad requires a total of three sequential 1PE processes prior to fluorescence emission, ideally resulting in an overall 3PE behavior. Herein, we report on the design, synthesis, and spectroscopic characterization of 2for1 and 3for1 systems, as well as the unprecedented experimental observation of a nonlinear response that goes well beyond that of 2PE by employing sequential 1PE.

### Results and Discussion

### Design

To facilitate understanding of the 3for1 function, the design of the corresponding dyad for a 2for1 function will be first explained. As previously indicated, 2for1 implies that the fluorescence response behaves as if triggered by 2PE although 1PE is used. The molecular dyad for this purpose consists of a fluorescent probe (F) covalently linked to a photoswitch (PS), schematically represented in Figure 1a.

**Figure 1:**
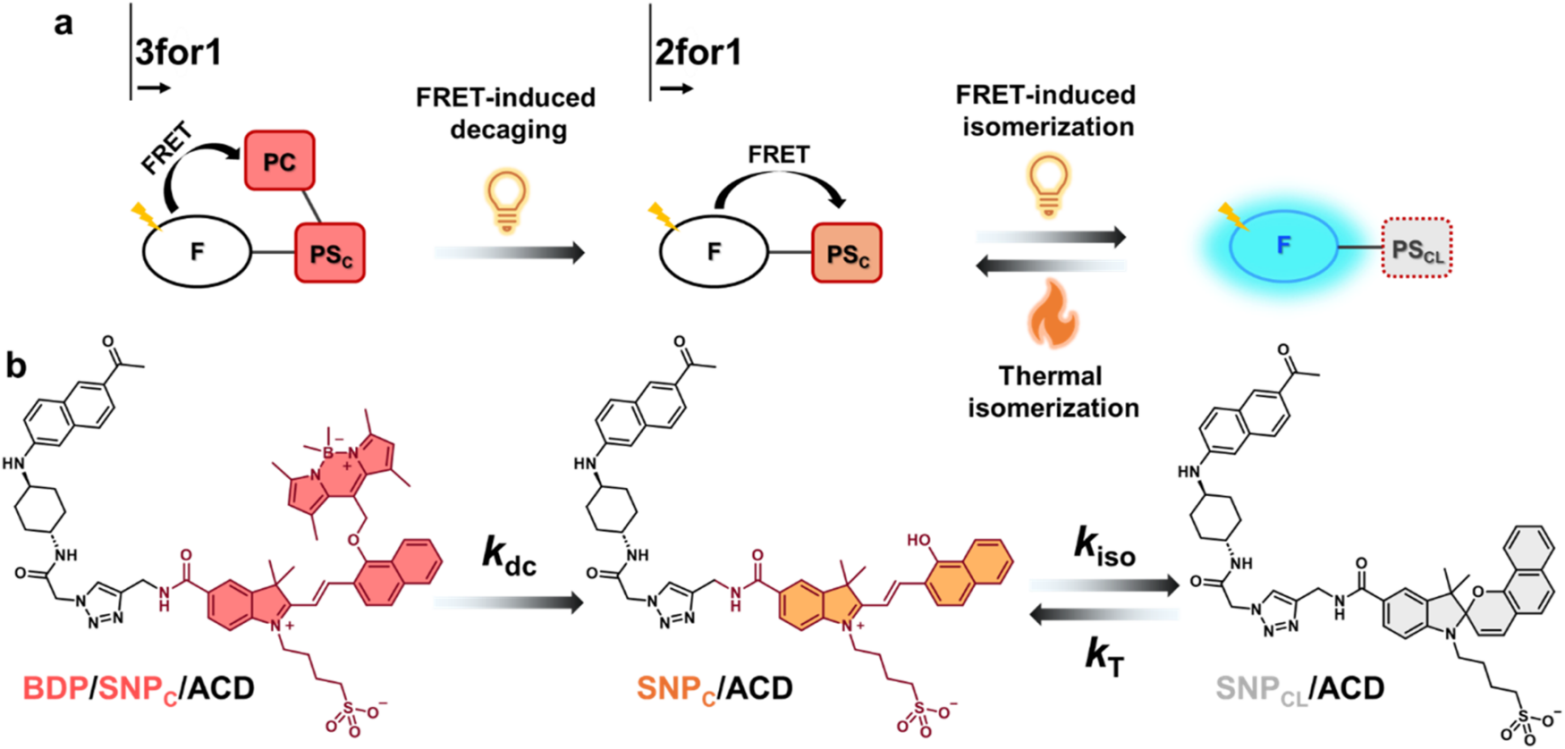
Schematic illustration and corresponding chemical structures. (a) Schematic representation of the design principles for the 3for1 triad and 2for1 dyad. F: Fluorophore, PS_C_: Colored isomer of photoswitch, PS_CL_: Colorless isomer of photoswitch, PC: Photocage. (b) Photochemical and thermal isomerization pathways for the 3for1 triad (**BDP/SNP_C_/ACD**) and the 2for1 dyad (**SNP_C_/ACD**). Upon FRET-induced decaging (*k*_dc_), the 3for1 triad releases the **BDP** unit to generate the 2for1 dyad, which subsequently undergoes FRET-induced isomerization (*k*_iso_) and thermal isomerization (*k*_T_). **SNP**_**CL**_ **/ACD** is the only fluorescent form. **BDP**: BODIPY, **SNP**: Spironaphthopyran, **ACD**: Acedan.

A conventional fluorescent probe F excited by a single photon emits light with an intensity *I*_f_ which is proportional to the excitation intensity *I*_exc_, that is, *I*_f_ ∝ *I*_exc_. In our study, we incorporated a T-type negative photoswitch to yield the F-PS dyad. The thermally stable form, to which the photoswitch reverts spontaneously in a thermal isomerization process, is the colored isomer PS_C_. Crucial for the function of the system is that the absorption of PS_C_ overlaps with the fluorescence of F, resulting in highly efficient Förster resonance energy transfer (FRET) from F to PS_C_. This FRET process quenches the fluorescence of F and renders the F-PS_C_ isomer nonfluorescent.

PS_C_ is isomerized to the colorless form PS_CL_ upon exposure to visible light. The absorption spectrum of PS_CL_ does not display any overlap with the fluorescence spectrum of F, thus there is no longer any FRET quenching of F. This is why F is highly fluorescent in the F-PS_CL_ isomeric form. In addition to photoisomerization triggered by direct excitation, PS_C_ is isomerized to PS_CL_ also upon FRET-induced sensitization.^28–30^ This implies that when PS_C_ is quenching the fluorescence of F in the F-PS_C_ form, it is isomerized to PS_CL_ to yield F-PS_CL_ in which F emits intense fluorescence. Irrespective of direct excitation or FRET-induced excitation, the following applies: the higher the intensity of the excitation light, *I*_exc_, the higher the concentration of the fluorescent isomer F-PS_CL_.

Under conditions when the rate of thermal isomerization back to the thermally stable nonfluorescent form F-PS_C_ is substantially faster than the corresponding rate of photoinduced formation of the fluorescent form F-PS_CL_, *the concentration of F-PS_CL_* *is directly proportional to I*_exc_. In addition, the intrinsic fluorescence intensity of F in the F-PS_CL_ population is of course also proportional to the excitation intensity. Hence, we have a situation in which the overall fluorescence intensity from the sample displays a quadratic dependence on the excitation intensity, that is, *I*_f_ ∝ *I*_exc_^2^. This quadratic dependence describes the nonlinear relation between the excitation and the emission intensity for a 2PE process, and therefore we would expect a 2PE response from the dyad although sequential 1PE was used. The overall process can be regarded as if one photon is needed to “activate” the dyad to the fluorescent form, and another photon is needed to trigger the excitation for fluorescent readout. The photophysical characterization of such a 2for1 dyad will be described in later sections.

The transition to a 3for1 construct requires the addition of a second “activation” step to the 2for1 sequence (Figure 1a). For this purpose, we introduced a photocage (PC) to the PS_C_ unit to afford the F-PS_C_-PC triad. The attachment of the PC inhibits the photoisomerization reaction PS_C_ → PS_CL_.^31^ This means that before photoisomerization can occur, PC must be decaged from the PS by the action of another photon, either by direct excitation of the PC or by FRET-induced sensitization. Therefore, in the 3for1 triad, the first photon is used to decage PC, a subsequent photon is responsible for the isomerization of F-PS_C_ to F-PS_CL_, and a third photon eventually excites F within the F-PS_CL_ isomer for emission readout (Figure 1a). Altogether, the requirement of three photons to observe fluorescence emission results in a cubic dependence of the fluorescence intensity *I*_f_ on the excitation intensity *I*_exc_, that is, *I*_f_ ∝ *I*_exc_^3^. A more stringent treatment of this kinetic situation is given in the Supporting Information (Section 4).

### Implementation

The selection of molecular candidates to implement our design concepts was primarily guided by spectroscopic criteria ensuring the proper function of the systems. Among the many potential candidates, an acedan derivative (**ACD**),^32^ a spironaphthopyran derivative (**SNP**),^33–36^ and a BODIPY derivative (**BDP**)^37–40^ were chosen as F, PS, and PC, respectively. The synthetic versatility of these building blocks, together with their complementary photophysical properties, made them suitable as building blocks for the molecular constructs for both 2for1 and 3for1.

A summary of the synthetic route of the **SNP/ACD** dyad (2for1) and the **BDP/SNP/ACD** triad (3for1) is shown in Scheme 1 (see Section 2 and Section 8 in the Supporting Information, for synthetic details and analytical characterization, respectively). In brief, we opted for a late-stage modular synthetic strategy, where the different building blocks were individually designed and synthesized. Accordingly, **SNP** and **BDP**/**SNP** were prepared via a Knoevenagel condensation between the reactive indolinium compound **S3**, bearing an alkyne moiety, and the corresponding aldehydes. Subsequently, **ACD**, containing an azido group, was conjugated with either **SNP** or **BDP/SNP** through Cu­(I)-catalyzed azide–alkyne cycloaddition reaction to afford the 2for1 dyad and the 3for1 triad, respectively.

**Scheme 1:**
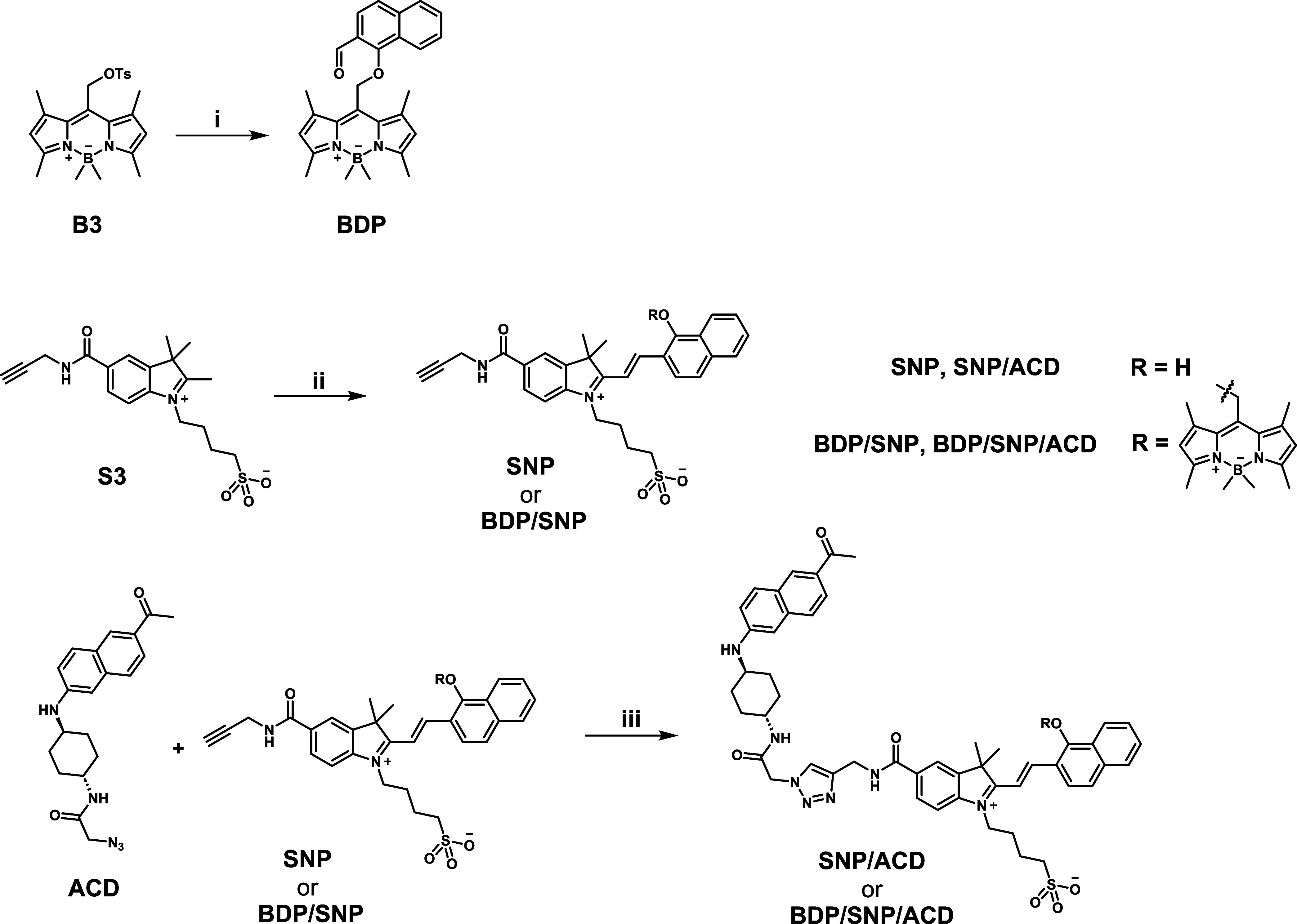
Synthesis of the 2for1 Dyad and the 3for1 Triad^a^

a Reagents and conditions: (i) 1-hydroxy-2-naphthaldehyde, K_2_CO_3_, KI, ACN, room temperature, 90% yield; (ii) 1-hydroxy-2-naphthaldehyde, EtOH, reflux, 53% yield or **BDP**, NaOAc, Ac_2_O, 60 °C, quantitative yield; (iii) [Cu­(CH_3_CN)_4_]­PF_6_, DMSO or DCM/MeOH (4/1), room temperature, 39 and 24% yield for **SNP/ACD** and **BDP/SNP/ACD**, respectively.

The isomerization/decaging schemes of **SNP**_**C**_ **/ACD** and **BDP/SNP**_**C**_ **/ACD** are shown in Figure 1b. **SNP** exists in a colored open merocyanine form **SNP**_**C**_, which is the thermally stable isomer under the conditions employed. Visible light exposure isomerizes **SNP**_**C**_ to the colorless spirocyclic form **SNP**_**CL**_. As previously indicated, FRET-induced sensitization triggers the same reaction. The rate constant of this process is referred to as *k*_iso._ Note that we are referring to the bulk rate constant occurring on a time scale of seconds, as opposed to the molecular rate constant occurring on the ps-ns time scale. Thermal isomerization triggers the reverse process back to **SNP**_**C**_ with the associated rate constant *k*_T_. For the BODIPY-based photocage **BDP**, the ether bond is broken to release the decaged compound upon photoinduced excitation (*k*_dc_).

### Photophysical/Spectroscopic Characterization

All spectroscopic measurements were performed in acidic (1 mM HCl) methanol solution containing 1% DMSO unless otherwise mentioned. In neutral methanol, **SNP**_**CL**_ is the predominant isomer (Figure S2a), precluding efficient FRET quenching (Figure S2b). Addition of HCl shifts the thermal equilibrium **SNP**_**C**_ ⇆ **SNP**_**CL**_ further toward **SNP**_**C**_, which is required for the intended function.^33,34^ Moreover, acidic medium was used to ensure a high concentration of the protonated form of **SNP**_**C**_, as this form has a much more efficient photoisomerization reaction compared to the nonprotonated form.^41,42^

The thermal stability of the compounds was not affected by the addition of acid (Figure S3a). This is true also for the stability during repeated photocycling (Figure S3b,c). The relevant spectra of the implicated monomers, dyads, and triad are shown in Figure 2.

**Figure 2:**
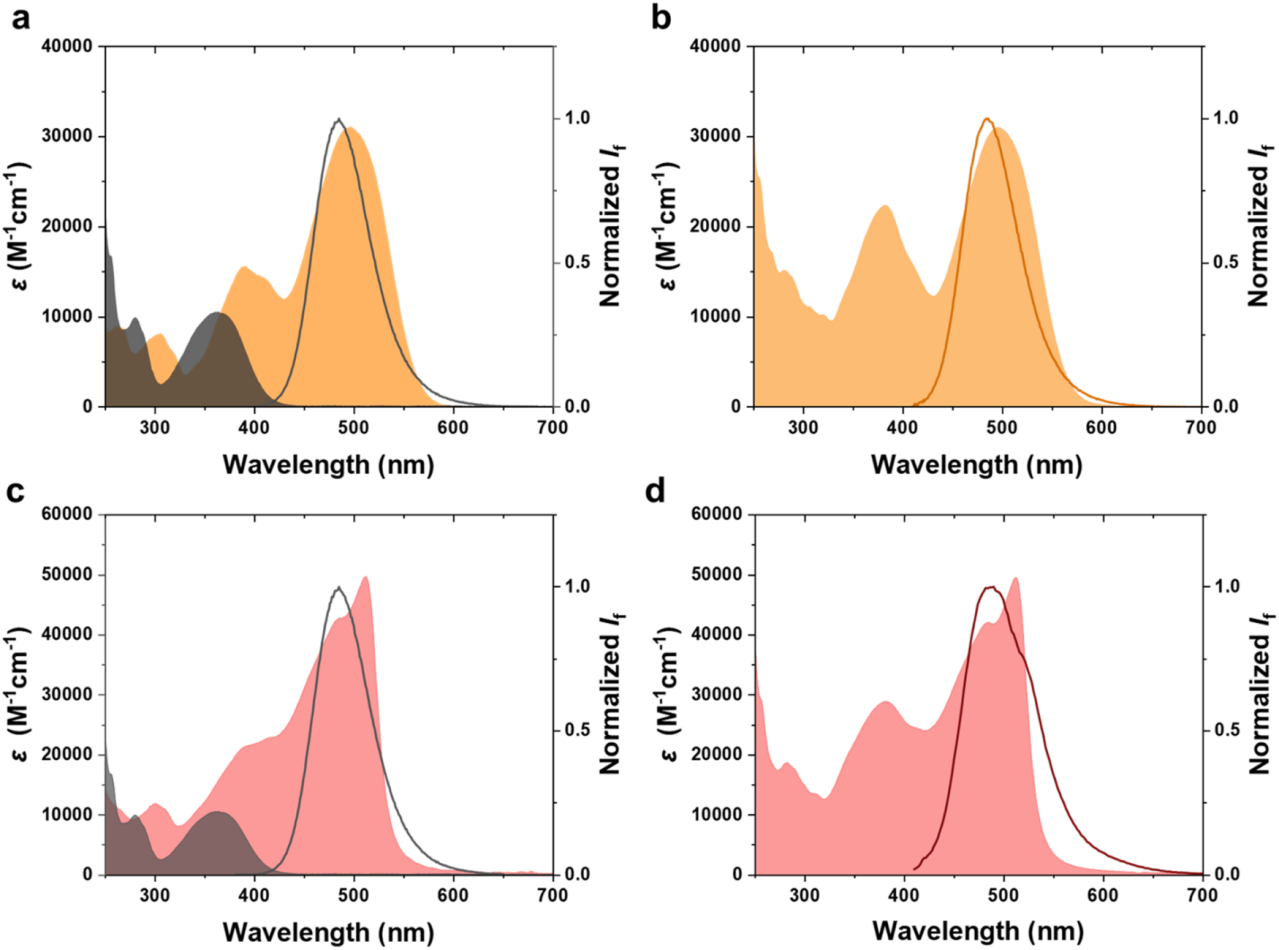
Absorption/emission spectra of the monomer, dyad and triad for 2for1 and 3for1. (a) Absorption (filled area) and emission spectra (solid line) of **ACD** (black) and **SNP**_**C**_ (orange). (b) Absorption spectrum (filled area) of the **SNP**_**C**_ **/ACD** dyad and emission spectrum (solid line) recorded after 405 nm irradiation of **SNP**_**C**_ **/ACD** to yield a significant population of the fluorescent form **SNP**_**CL**_ **/ACD**. (c) Absorption (filled area) and emission spectra (solid line) of **ACD** (black) and **BDP/SNP**_**C**_ (red). (d) Absorption spectrum (filled area) of the **BDP/SNP**_**C**_ **/ACD** triad and emission spectrum (solid line) recorded after 405 nm irradiation of **BDP/SNP**_**C**_ **/ACD** to yield a significant population of the fluorescent form **SNP**_**CL**_ **/ACD**. The shoulder at around 525 nm in the emission spectra originates from the minor BDP fluorescence centered at 524 nm.

The individual spectra of the building blocks are preserved in the conjugate, implying that **ACD** is electronically decoupled from **SNP**_**C**_ and **BDP/SNP**_**C**_ in the dyad and the triad, respectively (also confirmed by TDDFT calculations, see Section 5b in the Supporting Information). It is apparent from Figure 2 that there is a substantial overlap between the emission of **ACD** and the absorption of both **SNP**_**C**_ and **BDP/SNP**_**C**_. The overlap integrals were determined to be on the order of 10^15^ nm^4^ M^–1^ cm^–1^ using the FRET formalism (See Section 5a in the Supporting Information).

The **ACD** monomer emits fluorescence with a quantum yield of 0.29. This fluorescence is heavily quenched in the **SNP**_**C**_ **/ACD** dyad (quantum yield of 0.02) and the **BDP/SNP**_**C**_ **/ACD** triad (quantum yield of 0.003) as seen in Table 1, displaying photophysical data of all relevant compounds. The comparison of the fluorescence lifetimes of **ACD**, **SNP**_**C**_ **/ACD**, and **BDP/SNP**_**C**_ **/ACD** also reveals a dramatic quenching of the fluorescence in the dyad and triad. The fluorescence decays of the dyad and triad feature lifetimes of 2.3 and 1.9 ps, respectively, whereas the **ACD** monomer displays a lifetime of 2.0 ns, as determined from femtosecond up-conversion and time-correlated single-photon counting measurements (see Section 6 in the Supporting Information). This corresponds to nearly quantitative fluorescence quenching (>99%). Based on the critical FRET radius *R*_0_ (45 and 47 Å for the dyad and the triad, respectively), 98% and 99% of FRET efficiency is expected from Förster theory, with a center-to-center interchromophore distance of 23 Å for both the dyad and the triad (see Section 5a in the Supporting Information). Given that the FRET efficiencies determined from lifetime measurement and theoretical calculations are nearly quantitative, the residual background emission (BE, see Table 1) is attributed to the presence of the fluorescent isomer prior to photoisomerization.

**Table 1:** Photophysical Properties of the Monomers, Dyads, and Triad

	λ_abs_ (nm)^a^	ε (M^–1^cm^–1^)^b^	*t*_1/2_ (s)^c^	λ_em_ (nm)^d^	Φ_F_ ^e^	τ_F_ (ns)^f^	BE (%)^g^
ACD	363	10,600		485	0.29	2.0	
SNP_C_	500	30,900	0.57				
SNP_C_/ACD	497	30,900	0.87		0.02	0.0023	7
BDP/SNP_C_	512	49,700					
BDP/SNP_C_/ACD	512	49,500			0.003	0.0019	1

a UV/vis absorption maximum.

b Molar absorption coefficient.

c Thermal isomerization half-life determined by UV/vis absorption for **SNP**_**C**_ and fluorescence spectroscopy for **SNP**_**C**_ **/ACD**.

d Emission maximum upon excitation at 365 nm.

e Fluorescence quantum yield.

f Fluorescence lifetime, measured by time-correlated single-photon counting and femtosecond fluorescence up-conversion.

g Background emission, determined by comparing the steady-state emission of **SNP**_**C**_ **/ACD** to **ACD** which corresponds to the maximum attainable emission intensity of the dyad.

### Nonlinearity Factor

To facilitate the performance evaluation of our compounds, we introduce the nonlinearity factor (NLF), which corresponds to the slope of the double-logarithmic plot of fluorescence intensity *I*_f_ vs excitation intensity *I*_exc_. The NLF indicates in a straightforward manner how many photons are required for the observation of fluorescence emission, that is, it quantifies the degree of nonlinearity: NLF = 1 for linear 1PE (1for1), 2 for 2PE (2for1), and 3 for 3PE (3for1).

### Performance Validation of the 2for1 System

From the data above, it is seen that **ACD** emits only weak fluorescence in the **SNP**_**C**_ **/ACD** dyad which is in line with the design criteria described above. Next, experiments were undertaken to show that the **ACD** fluorescence intensity increases in the **SNP/ACD** dyad upon extended visible light exposure to trigger the isomerization from **SNP**_**C**_ **/ACD** to **SNP**_**CL**_ **/ACD**. This was investigated by monitoring the emission intensity *I*_f_ at 485 nm (where **ACD** is the sole emitter) upon continuous exposure to 375 nm light at different intensities *I*_exc_. It is noteworthy that at this irradiation wavelength direct as well as FRET-sensitized photoisomerization of **SNP**_**C**_ apply, both having the same effect in the outlined 2for1 approach. The results are displayed in Figure 3 for two excitation intensities: *I*_exc_ = 39 and 78 mW/cm^2^, referred to as Half *I*_exc_ and Full *I*_exc_, respectively.

**Figure 3:**
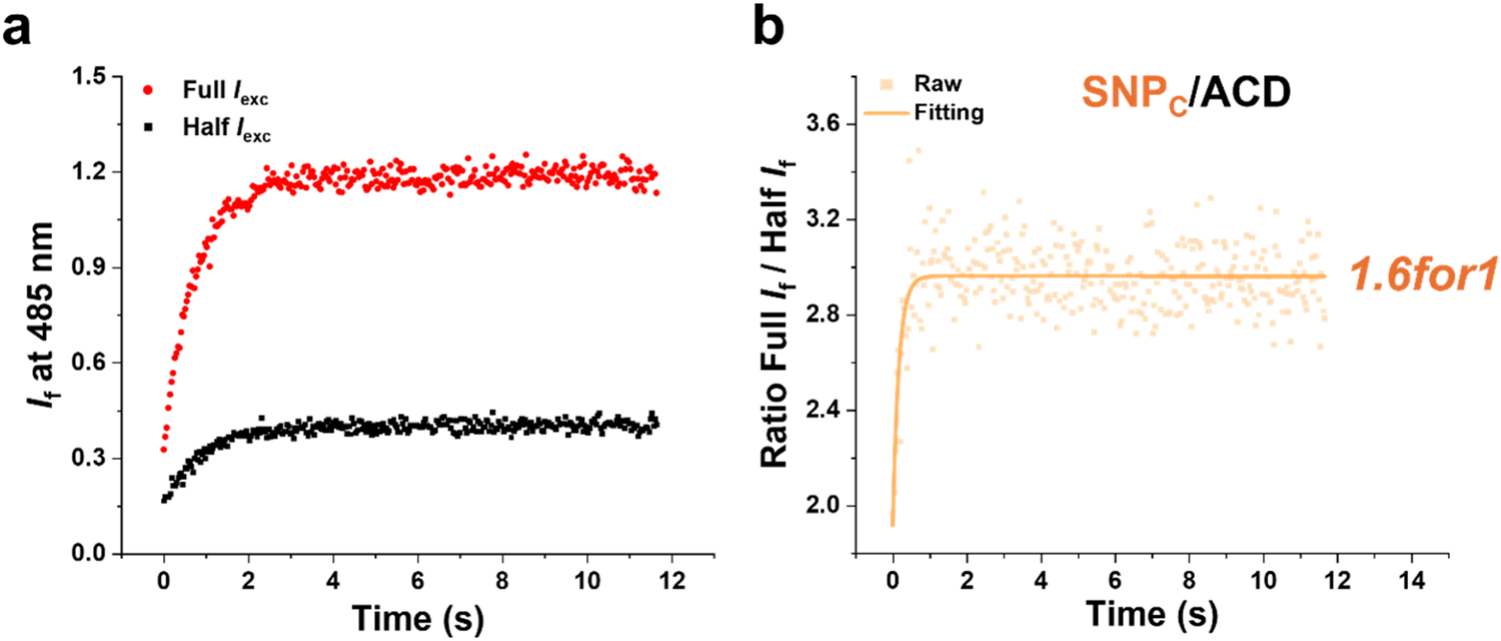
Time-dependent fluorescence responses of the **SNP**_**C**_ **/ACD** dyad under continuous irradiation with light at two excitation intensities. (a) Fluorescence kinetics of **SNP**_**C**_ **/ACD** upon 375 nm irradiation at Half *I*_exc_ (39 mW/cm^2^, black) and Full *I*_exc_ (78 mW/cm^2^, red). (b) Ratio of fluorescence intensities shown in panel a (dots) and exponential fitting (solid line). An NLF of 1.6 was observed at the plateau. See text for details.

The time evolution of the fluorescence intensities is well described by a monoexponential process with rate constant *k*_obs_. The *k*_obs_ reflects the rate of the equilibrium establishment between **SNP**_**C**_ **/ACD** and **SNP**_**CL**_ **/ACD**, referred to as the “photothermal” equilibrium. This rate constant equals the sum of *k*_iso_ and *k*_T_, as defined in Figure 1b, so that *k*_iso_ can be extracted when *k*_obs_ and *k*_T_ are known. Here, it is encouraging to note that the rate of the photoinduced reaction **SNP**_**C**_ **/ACD** → **SNP**_**CL**_ **/ACD** increases by a factor of 2 (*k*_iso_ increases from 0.3 s^–1^ to 0.6 s^–1^) when doubling the excitation intensity (see Section 4a in the Supporting Information for more details about the determination of *k*_obs_ and *k*_T_). This confirms that photoisomerization of the **SNP** photoswitch in the **SNP/ACD** dyad is a pure 1P process which is in line with our theoretical framework.

After the initial rise, the signal reaches a plateau intensity. Here, the photoinduced isomerization **SNP**_**C**_ **/ACD** → **SNP**_**CL**_ **/ACD** is counterbalanced by the reverse thermal isomerization reaction **SNP**_**CL**_ **/ACD** → **SNP**_**C**_ **/ACD**, implying that the photothermal equilibrium position is reached. As expected, the plateau intensity is higher when using the higher excitation intensity. Any conventional fluorophore excited in a 1P process would display an increase in the fluorescence intensity by a factor 2 upon doubling the excitation intensity (see Section 3e for the details of the 1PE characterization of **ACD** in the Supporting Information). For our **SNP/ACD** dyad, however, the fluorescence intensity at the plateau is increased by a factor 3.0, which corresponds to NLF of 1.6 (Figure 3b).

This is a clear manifestation of the 2for1 phenomenon, resulting from the fact that the photothermal equilibrium position is more enriched in the fluorescent isomer **SNP**_**CL**_ **/ACD** when doubling the excitation intensity. To further validate this notion quantitatively, a kinetic model where the fluorescence signal results from a 2for1 process was developed and it was compared with the experimentally determined NLF. The experimental results align well with kinetic modeling, confirming the expected 2for1 mechanism (See Figures S9 and S11 and Section 4a in the Supporting Information).

In the ideal 2for1 situation, the plateau intensity would increase by a factor 4 (2^2^ = 4) upon doubling the excitation intensity. This discrepancy can be rationalized by the following considerations. In our case, it is obvious from Figure 3a that the emission intensity is nonzero at *t* = 0, that is before any photoinduced isomerization to the fluorescent form occurs. We refer to this emission as background emission (BE). The BE was determined to be 7% of the maximum attainable fluorescence intensity arising from a sample fully isomerized to the fluorescent **SNP**_**CL**_ **/ACD** isomer.

The BE intensity is directly proportional to the excitation intensity, implying that an ideal 2for1 behavior will never be observed in the presence of BE. To deconvolute the emission that stems from photoinduced isomerization, we subtracted the BE intensity from the two traces in Figure 3a. This exercise yielded a plateau ratio of 3.6, which corresponds to NLF of 1.9 (Figure S4). Thus, after compensation for BE, the dyad displays a nearly pure quadratic dependence *I*_f_ ∝ *I*_exc_^2^, which is fully in line with our design criteria for the dyad.

### Performance Validation of the 3for1 System

As mentioned above, adding a second “activation step” to the sequential series of 1PE is expected to ideally increase the NLF from 2 to 3. Moreover, it is evident that the BE should be minimized to maximize the NLF. A proper choice of an additional photoresponsive substituent attached to the dyad could in fact fulfill these requirements by introducing a second activation step while simultaneously reducing the BE. The most likely reason for the presence of 7% BE is the establishment of a dark equilibrium containing ca. 7% of the undesired fluorescent isomer **SNP**_**CL**_ **/ACD**. The optimal additional photoresponsive substituent should therefore minimize the amount of **SNP**_**CL**_ **/ACD** before light exposure (decreasing the BE), while allowing for photoinduced formation of the same isomer (a second activation step).

The photophysical data for the **BDP/SNP**_**C**_ **/ACD** triad synthesized for this purpose are shown in Table 1. It is very encouraging to note that the BE is effectively reduced to 1% in the **BDP/SNP**_**C**_ **/ACD** triad, which can be attributed to the introduction of the **BDP** cage. *I*_f_ at 470 nm was monitored with time for the **BDP/SNP**_**C**_ **/ACD** triad under exposure to light at 405 nm at two different excitation intensities: *I*_exc_ = 80 and 160 mW/cm^2^, referred to as Half *I*_exc_ and Full *I*_exc_, respectively (Figure 4a). The ratio between these fluorescence traces is shown on a logarithmic time-axis in Figure 4b. To quantitatively rationalize the fluorescence response with time, kinetic modeling was performed for a 3for1 process (solid line in Figure 4b; see Section 4b in the Supporting Information for details).

**Figure 4:**
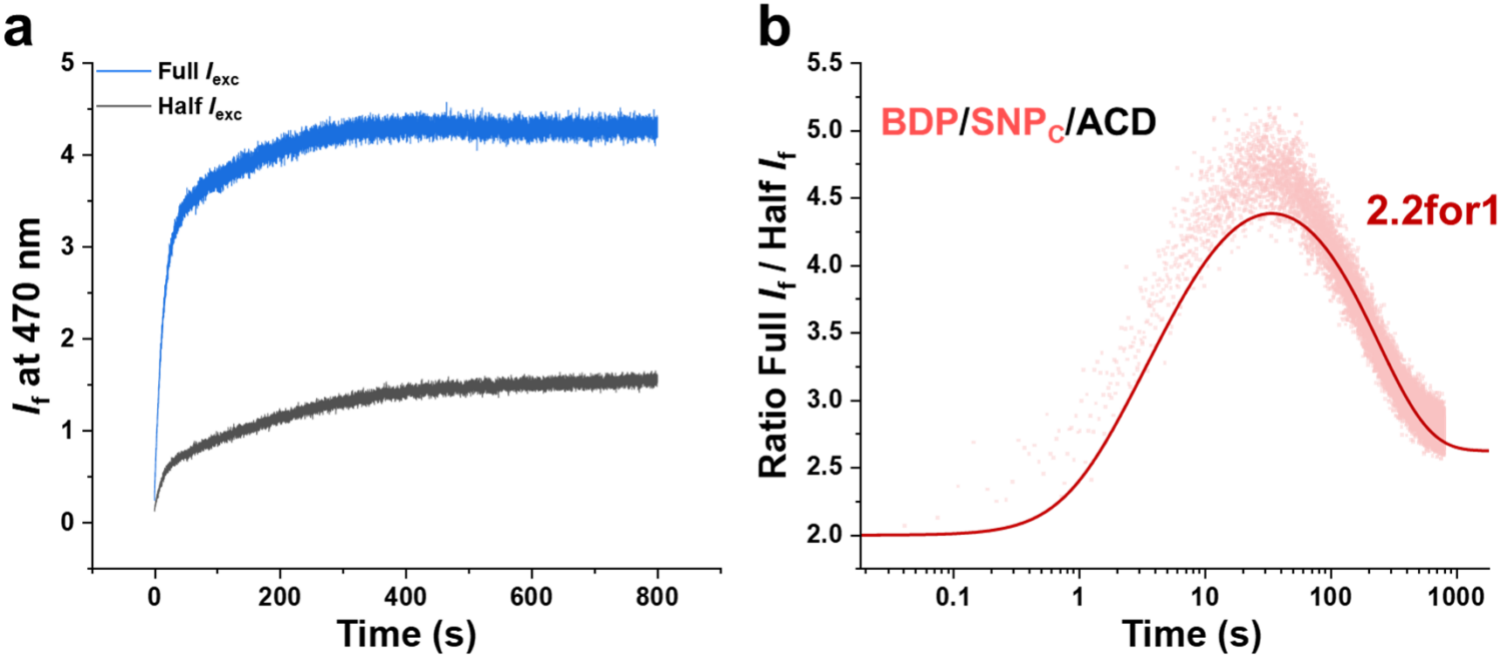
Time-dependent fluorescence responses of the **BDP/SNP**_**C**_ **/ACD** triad under continuous irradiation at two excitation intensities. (a) Fluorescence kinetics of **BDP/SNP**_**C**_ **/ACD** upon 405 nm irradiation at Half *I*_exc_ (80 mW/cm^2^, black) and Full *I*_exc_ (160 mW/cm^2^, blue). (b) Ratio of fluorescence intensities (dots) and kinetic simulation (solid line) performed using parameters determined from experimental data. A maximum NLF of 2.2 was observed.

In the absence of any BE at all, the ratio presented in Figure 4b should be equal to 8 at *t* = 0 and decrease until the photothermal equilibrium is reached at longer times. This is due to the fact that for a 3PE process, doubling the excitation intensity would result in an 8-fold increase in the emission intensity (2^3^ = 8). In our case, the experimentally obtained trace instead starts at ca. 2 at *t* = 0 as seen in Figure 4b. The highly satisfying agreement between experimental and simulated data demonstrated that this deviation is fully accounted for by the negative influence of the 1% BE at low degrees of isomerization to the fluorescent isomer **SNP**_**CL**_ **/ACD**. After an initial rise to reach a maximum value of ca. 4.6, the ratio of the fluorescence intensities at Full and Half excitation power decreases again to a plateau level of ca. 2.8, which agrees well with the performance of the **SNP**_**C**_ **/ACD** formed after decaging. The maximum value of 4.6 corresponds to a NLF of 2.2, that is, *I*_f_ ∝ *I*_exc_^2.2^ which is equivalent to a 2.2for1 function. The discrepancy between the observed NLF of 2.2 and the ideal value of 3 is, again, a predictable consequence of the BE (see Figure S14).

Although an NLF of 2.2 is relatively far from the theoretically obtainable value of 3, it should be noted that 2.2 is a substantial improvement compared to the same value of 1.6 observed for the dyad. This illustrates the positive effect from introducing the **BDP** cage, both to decrease the initial BE from 7% to 1% and to add the extra activation step. It is acknowledged that the irreversibility of the **BDP** decaging reaction does not allow for repeated use of the triad, as it is converted to the corresponding dyad in the process. Nevertheless, the results and the kinetic modeling establish the molecular design principle that sequential 1PE events enable higher-order nonlinearity beyond 2PE. The good agreement between the experiment and modeling of 2for1 dyad and 3for1 triad validates the underlying sequential 1PE mechanism (see Section 4 in the Supporting Information for a comprehensive description of the data modeling).

It is obvious that any modification of the molecular design that reduces the BE will have a positive effect on the NLF. Alternative photoswitches with a thermal equilibrium position strongly shifted to the colored FRET-active isomer, ideally without acidification, would be worthwhile candidates. Moreover, replacing the irreversible photodecaging with a reversible photoreaction would not only enable repeated use but also prevent unwanted decaging by stray light prior to controlled irradiation.

## Conclusions

In summary, we demonstrated molecular design principles for higher-order nonlinear fluorescence response using sequential 1PE. The presented molecular constructs are composed of a fluorophore covalently linked to a T-type negative photoswitch (2for1 dyad) together with a photocage (3for1 triad). The design implies that one or two photons must be absorbed to activate the dyad and the triad, respectively, to the fluorescent isomeric form. Absorption of the last photon is used to stimulate the emission of fluorescence. Hence, although each individual step is triggered by 1PE, a total of two and three photons must be sequentially absorbed before fluorescence is emitted in the dyad and in the triad, respectively. Thus, the compounds have the capacity to emit fluorescence where the intensity *I*_f_ is proportional to the square and the cube of the excitation intensity *I*_exc_, that is, to display the same dependence as for conventional two- and three-photon absorbing probes. The experimental results show that the dyad displays a nonlinear behavior near 2PE, whereas the nonlinearity of the triad extends significantly beyond that of 2PE. Overall, these findings demonstrate that molecular engineering strategies can effectively emulate complex multiphoton processes using conventional light sources, providing a promising blueprint for future nonlinear optical applications without the need for sophisticated instrumentation.

## Supplementary Material



## Notes

Experimental details, details on synthesis of compounds and characterization data (NMR, HRMS), additional spectral data, details on kinetic modeling and theoretical calculations (PDF)

## References

[1] Lakowicz, J. R. Principles of Fluorescence Spectroscopy; Springer, 2006.

[2] Ntziachristos V. (2006). Fluorescence
Molecular Imaging. Annu. Rev. Biomed. Eng..

[3] Hötzer B., Medintz I. L., Hildebrandt N. (2012). Fluorescence
in Nanobiotechnology:
Sophisticated Fluorophores for Novel Applications. Small.

[4] Yang Z., Mao Z., Xie Z., Zhang Y., Liu S., Zhao J., Xu J., Chi Z., Aldred M. P. (2017). Recent Advances in Organic Thermally
Activated Delayed Fluorescence Materials. Chem.
Soc. Rev..

[5] Wang C., Li Z. (2017). Molecular Conformation
and Packing: Their Critical Roles in the Emission
Performance of Mechanochromic Fluorescence Materials. Mater. Chem. Front..

[6] Lichtman J. W., Conchello J.-A. (2005). Fluorescence Microscopy. Nat.
Methods.

[7] Michalet X., Kapanidis A. N., Laurence T., Pinaud F., Doose S., Pflughoefft M., Weiss S. (2003). The Power and Prospects of Fluorescence
Microscopies and Spectroscopies. Annu. Rev.
Biophys. Biomol. Struct..

[8] Yao J., Yang M., Duan Y. (2014). Chemistry,
Biology, and Medicine
of Fluorescent Nanomaterials and Related Systems: New Insights into
Biosensing, Bioimaging, Genomics, Diagnostics, and Therapy. Chem. Rev..

[9] Albota M., Beljonne D., Bredas J.-L., Ehrlich J. E., Fu J.-Y., Heikal A. A., Hess S. E., Kogej T., Levin M. D., Marder S. R. (1998). Design of Organic Molecules with Large Two-Photon
Absorption Cross Sections. Science.

[10] Helmchen F., Denk W. (2005). Deep Tissue Two-Photon
Microscopy. Nat. Methods.

[11] Denk W., Strickler J. H., Webb W. W. (1990). Two-Photon Laser Scanning Fluorescence
Microscopy. Science.

[12] Pawlicki M., Collins H. A., Denning R. G., Anderson H. L. (2009). Two-Photon Absorption
and the Design of Two-Photon Dyes. Angew. Chem.,
Int. Ed..

[13] Terenziani F., Katan C., Badaeva E., Tretiak S., Blanchard-Desce M. (2008). Enhanced Two-Photon
Absorption of Organic Chromophores: Theoretical and Experimental Assessments. Adv. Mater..

[14] He G. S., Markowicz P. P., Lin T.-C., Prasad P. N. (2002). Observation of Stimulated
Emission by Direct Three-Photon Excitation. Nature.

[15] He G. S., Bhawalkar J. D., Prasad P. N., Reinhardt B. A. (1995). Three-Photon-Absorption-Induced
Fluorescence and Optical Limiting Effects in an Organic Compound. Opt. Lett..

[16] Xu C., Zipfel W., Shear J. B., Williams R. M., Webb W. W. (1996). Multiphoton
Fluorescence Excitation: New Spectral Windows for Biological Nonlinear
Microscopy. Proc. Natl. Acad. Sci. U.S.A..

[17] Horton N. G., Wang K., Kobat D., Clark C. G., Wise F. W., Schaffer C. B., Xu C. (2013). In Vivo Three-Photon
Microscopy of
Subcortical Structures within an Intact Mouse Brain. Nat. Photonics.

[18] Szmacinski H., Gryczynski I., Lakowicz J. R. (1996). Three-Photon Induced
Fluorescence
of the Calcium Probe Indo-1. Biophys. J..

[19] Maiti S., Shear J. B., Williams R., Zipfel W., Webb W. W. (1997). Measuring
Serotonin Distribution in Live Cells with Three-Photon Excitation. Science.

[20] Lakowicz J. R., Gryczynski I., Malak H., Schrader M., Engelhardt P., Kano H., Hell S. W. (1997). Time-Resolved Fluorescence
Spectroscopy
and Imaging of DNA Labeled with Dapi and Hoechst 33342 Using Three-Photon
Excitation. Biophys. J..

[21] Nilsson J. R., Benitez-Martin C., Sansom H. G., Pfeiffer P., Baladi T., Le H.-N., Dahlén A., Magennis S. W., Wilhelmsson L. M. (2023). Multiphoton
Characterization and Live Cell Imaging Using Fluorescent Adenine Analogue
2CNqA. Phys. Chem. Chem. Phys..

[22] He G. S., Tan L.-S., Zheng Q., Prasad P. N. (2008). Multiphoton Absorbing
Materials: Molecular Designs, Characterizations, and Applications. Chem. Rev..

[23] Prevedel R., Ferrer Ortas J., Kerr J. N. D., Waters J., Breckwoldt M. O., Deneen B., Monje M., Soyka S. J., Venkataramani V. (2025). Three-Photon
Microscopy: An Emerging Technique for Deep Intravital Brain Imaging. Nat. Rev. Neurosci..

[24] Xiao Y., Deng P., Zhao Y., Yang S., Li B. (2023). Three-Photon
Excited Fluorescence Imaging in Neuroscience: From Principles to Applications. Front. Neurosci..

[25] Hoover E. E., Squier J. A. (2013). Advances in Multiphoton
Microscopy Technology. Nat. Photonics.

[26] Kobayashi Y., Abe J. (2022). Recent Advances in
Low-Power-Threshold Nonlinear Photochromic Materials. Chem. Soc. Rev..

[27] Kobayashi Y., Mutoh K., Abe J. (2016). Fast Photochromic Molecules toward
Realization of Photosynergetic Effects. J. Phys.
Chem. Lett..

[28] Benitez-Martin C., Li S., Dominguez-Alfaro A., Najera F., Pérez-Inestrosa E., Pischel U., Andréasson J. (2020). Toward Two-Photon Absorbing Dyes
with Unusually Potentiated Nonlinear Fluorescence Response. J. Am. Chem. Soc..

[29] Benitez-Martin C., Rouillon J., Fron E., de Jong F., Grøtli M., Hofkens J., Pischel U., Andréasson J. (2025). Exploiting
Negative Photochromism to Harness a Four-Photon-Like Fluorescence
Response with Two-Photon Excitation. Nat. Commun..

[30] Song L., Jares-Erijman E., Jovin T. (2002). A Photochromic Acceptor as a Reversible
Light-Driven Switch in Fluorescence Resonance Energy Transfer (FRET). J. Photochem. Photobiol., A: Chem..

[31] Fleming C. L., Li S., Grøtli M., Andréasson J. (2018). Shining New Light on the Spiropyran
Photoswitch: A Photocage Decides between Cis–Trans or Spiro-Merocyanine
Isomerization. J. Am. Chem. Soc..

[32] Singha S., Kim D., Roy B., Sambasivan S., Moon H., Rao A. S., Kim J. Y., Joo T., Park J. W., Rhee Y. M. (2015). A Structural Remedy
toward Bright Dipolar Fluorophores in Aqueous
Media. Chem. Sci..

[33] Hammarson M., Nilsson J. R., Li S., Beke-Somfai T., Andréasson J. (2013). Characterization of the Thermal and Photoinduced Reactions
of Photochromic Spiropyrans in Aqueous Solution. J. Phys. Chem. B.

[34] Wimberger L., Prasad S. K. K., Peeks M. D., Andréasson J., Schmidt T. W., Beves J. E. (2021). Large, Tunable,
and Reversible pH
Changes by Merocyanine Photoacids. J. Am. Chem.
Soc..

[35] Shiraishi Y., Takagi S., Yomo K., Hirai T. (2021). Spontaneous Isomerization
of a Hydroxynaphthalene-Containing Spiropyran in Polar Solvents Enhanced
by Hydrogen Bonding Interactions. ACS Omega.

[36] Moldenhauer D., Gröhn F. (2017). Water-Soluble
Spiropyrans with Inverse Photochromism
and Their Photoresponsive Electrostatic Self-Assembly. Chem. - Eur. J..

[37] Slanina T., Shrestha P., Palao E., Kand D., Peterson J. A., Dutton A. S., Rubinstein N., Weinstain R., Winter A. H., Klán P. (2017). In Search of the Perfect Photocage:
Structure–Reactivity Relationships in Meso-Methyl Bodipy Photoremovable
Protecting Groups. J. Am. Chem. Soc..

[38] Shrestha P., Kand D., Weinstain R., Winter A. H. (2023). Meso-Methyl Bodipy
Photocages: Mechanisms, Photochemical Properties, and Applications. J. Am. Chem. Soc..

[39] Peterson J. A., Fischer L. J., Gehrmann E. J., Shrestha P., Yuan D., Wijesooriya C. S., Smith E. A., Winter A. H. (2020). Direct Photorelease
of Alcohols from Boron-Alkylated Bodipy Photocages. J. Org. Chem..

[40] Rubinstein N., Liu P., Miller E. W., Weinstain R. (2015). Meso-Methylhydroxy Bodipy: A Scaffold
for Photo-Labile Protecting Groups. Chem. Commun..

[41] Feeney M. J., Thomas S. W. (2018). Tuning the Negative Photochromism
of Water-Soluble Spiropyran Polymers. Macromolecules.

[42] Kortekaas L., Chen J., Jacquemin D., Browne W. R. (2018). Proton-Stabilized
Photochemically Reversible E/Z Isomerization of Spiropyrans. J. Phys. Chem. B.

