# Supplementary material for: Molecular Engineering for Nonlinear Fluorescence: En Route to Three-Photon Absorption via Sequential One-Photon Excitation
Source: J Am Chem Soc. 2026 May 1;148(20):20813–20. doi: 10.1021/jacs.6c03621 (PMC13220266; doi:10.1021/jacs.6c03621)
Supplement: Supplementary file 1 [file ja6c03621_si_001.pdf]

Supporting Information

Molecular Engineering for Nonlinear Fluorescence: *En route* to Three-Photon Absorption via Sequential One-Photon Excitation

Jinyoung Oh,[1] Carlos Benitez-Martin,[1,2] Eduard Fron,[3,4] Johan Hofkens,[3,5] Uwe Pischel,[6]* Morten Grøtli,[1]* Joakim Andréasson[2]**

[1] *Department of Chemistry and Molecular Biology, University of Gothenburg, SE-41296 Göteborg, Sweden;* E-mail: grotli@chem.gu.se

[2] *Chemistry and Chemical Engineering, Chemistry and Biochemistry, Chalmers University of Technology. SE-41296 Göteborg,* Sweden; E-mail: a-son@chalmers.se

[3] *Department of Chemistry, KU Leuven, Celestijnenlaan 200F, B-3001 Leuven, Belgium;* E-mail: johan.hofkens@kuleuven.be

[4] *Core Facility for Advanced Spectroscopy, KU Leuven, Celestijnenlaan 200F, B-3001 Leuven, Belgium*

[5] *Max Planck Institute for Polymer Research, Ackermannweg 10, 55128 Mainz, Germany*

[6] *CIQSO – Center for Research in Sustainable Chemistry and Department of Chemistry, University of Huelva, Campus de El Carmen s/n, E-21071 Huelva, Spain;* uwe.pischel@diq.uhu.es

Table of Contents

1. Experimental Methods 3

1a. Materials and instruments for synthesis 3

1b. General methods and materials for optical spectroscopy 4

1c. General methods for theoretical calculations 4

1d. General methods for lifetime determination 5

2. Synthesis 7

3. Optical spectroscopy 17

3a. Absorption spectra of **SNP$_{C}$** and **SNP$_{CL}$** 17

3b. Fluorescence background emission upon HCl addition 18

3c. Chemical and photostability 19

3d. BE compensation for 2for1 performance validation in **SNP$_{C}$/ACD** 20

3e. Behavior of monomer fluorophore **ACD** in 1PE 21

4. Kinetic modeling 22

4a. Modeling of **SNP$_{C}$/ACD** 2for1 behavior 22

4b. Modeling of **BDP/SNP$_{C}$/ACD** 3for1 behavior 27

5. Theoretical calculations 30

5a. Optimized structure at ground state 30

5b. TDDFT calculations 32

6. Lifetime measurements 35

6a. TCSPC for the determination of monomer lifetime 35

6b. fs-UC for FRET characterization 35

7. References 36

8. NMR spectra 37

9. Atomic coordinates of optimized structures at the ground state 60

1. Experimental Methods

1a. Materials and instruments for synthesis

Commercially available reagents and solvents were purchased and used without further purification. Dry toluene, acetonitrile (ACN), and dichloromethane (DCM) were prepared from a solvent purification system (PS-MD-5/7 Inert Technology). Microwave reactions were performed in a Biotage Initiator Reactor using single-mode irradiation, with controlled temperature and pressure. Reactions were monitored by TLC (Merck TLC Silica gel 60 F_{254}), LC-MS (Waters, Acquity Arc HPLC system; 2489 UV/Vis Detector; XBridge BEH C18 column, 130Å, 2.5 µm, 2.1 x 50 mm; XBridge BEH C18 Guard column, V-Gd Cart 2.5 µm, 2.1 x 5 mm; Acquity QDa Mass Detector; ACN, H_2O with 0.01% formic acid, 40°C), and HPLC (Waters, 2690 Separation Module; 996 Photodiode Array Detector; Chromolith Speed ROD RP-18 endcapped 50-4.6 HPLC column; ACN, H_2O with 0.1% TFA). Flash-column chromatography was performed either on a Biotage Isolera or a Biotage Selekt flash chromatography system with silica gel columns/cartridges (Sfär Silica D Duo 60 µm for normal phase and Sfär C18 D Duo 100 Å 30 µm for reversed phase).

NMR spectra were obtained using a Bruker Avance NEO spectrometer, operating at 600 and 151 MHz for ^{1}H and ^{13}C, respectively. Chemical shifts (δ) are reported in parts per million (ppm) with reference to the residual protic solvent peak of $CDCl_3$, DMSO-d_6, or CD_3OD. Abbreviations for the multiplicity in ^{1}H NMR spectra are as follows: s = singlet, d = doublet, t = triplet, q = quartet, m = multiplet. Coupling constants (J) are reported in Hz.

HRMS data were recorded with a QExactive HF Orbitrap mass spectrometer interfaced with Dionex Ultimate 3000 liquid chromatography system (Thermo Fisher Scientific). The instrument operated in full MS mode only, where the ion mass spectra were acquired at a resolution of 120 000, maximum injection time 200 ms for $3x10^{6}$ ions. The Orbitrap was calibrated with Pierce LTQ ESI Positive Ion Calibration Solution prior to the analysis, resulting in a mass accuracy better than 5 ppm. Electrospray ionization was performed at 4 kV and 320 °C using a metal emitter in the ion source. The sample (1 or 10 µL) was injected onto a reversed-phase XBridge BEH C18 column (3.5 µm, 2.1 x 50 mm, Waters). The analysis was performed using a linear gradient over 2.5 min from 10 to 100% solvent B, followed by isocratic elution with 100% solvent B for 17.5 min with a flow of 0.300 mL/min (solvent A: H_2O with 0.1% formic acid; solvent B: 80% ACN in

H_2O with 0.1% formic acid). Data analysis was performed using the Xcalibur software (Thermo Fisher Scientific).

1b. General methods and materials for optical spectroscopy

All photophysical studies were performed in spectroscopic grade solvents at room temperature using 1 cm path-length quartz cuvettes, unless otherwise stated.

Ground state absorption spectra were recorded on a Cary 50 UV-Vis-NIR spectrometer. For photo- and thermal isomerization measurements, samples were irradiated using LEDs (LED Engin) centered around 365 nm (LZ1-10UV100, FWHM = 12 nm) and 405 nm (LZ1-10UB00-00U8, FWHM = 19 nm). Fluorescence quantum yields were determined using coumarin 153 in EtOH as a reference. Steady-state fluorescence spectra were recorded on a Cary Eclipse fluorimeter or, for the excitation intensity dependent fluorescence studies, on a home-built system consisting of continuous-wave OBIS lasers (Coherent) at 375 nm (Beam diameter = 0.7 mm) and 405 nm (Beam diameter = 0.8 mm) as the excitation source, a 1681 SPEX monochromator, and a 9-stage photomultiplier tube (PMT) detector. To vary the laser power, a linear variable neutral density (ND) filter was used, and the data were recorded using a home-built LabVIEW software.

1c. General methods for theoretical calculations

Gaussian 16.C.01[1] was employed for the theoretical calculations.

The geometrical parameters for the ground state (S_0) were determined with the density functional level of theory (DFT), employing the M062X[2] and the Def2TZVPP basis set[3]. Solvent effects were considered by including the solvation model based on density (SMD).[4] The absolute nature of the energetic minima was established by the absence of a negative frequency in the vibrational analysis.

Energy parameters were calculated as vertical electronic excitations from the S0 minima structure using the linear response (LR) approach and the time-dependent density functional response theory (TDDFT).[5] These calculations were carried out for the fifteen first excited states at the SMD(water)/M062X/Def2TZVPP level.

Natural Transition Orbitals (NTOs) were analyzed to confirm the absence of electronic interactions within the dyads and triads. These orbitals result from combining proportionally the different elementary orbitals that participate in an electronic transition, thus giving a better description of its nature.[6]

1d. General methods for lifetime determination

The fluorescence decay times at the nanosecond time scale were determined using the time-correlated single photon counting (TCSPC) technique. The frequency-doubled output (360–400 nm, 81 MHz, 2 ps FWHM) of a mode-locked Ti:Sapphire laser (Tsunami, Spectra Physics) was used as the excitation source. The repetition rate of the laser system was reduced to 8.1 MHz by a pulse picker (Spectra-Physics). The linearly polarized excitation light was rotated to a vertical plane using a Berek compensator (New Focus) in combination with a polarization filter and directed onto the sample.

The samples, in solution form with an OD of 0.1, were placed in a quartz cuvette (10 mm path length) sealed with a Teflon stopper. The emission was collected at a 90° angle relative to the incident light and guided through a polarization filter set to the magic angle (54.7°) with respect to the polarization plane of the excitation beam. The fluorescence was spectrally resolved using a double monochromator (Sciencetech 9030, 100 nm focal length, wavelength accuracy 0.3 nm) and detected by a microchannel plate photomultiplier tube (MCP-PMT, R3809U-51, Hamamatsu).

A time-correlated single-photon timing PC module (PicoHarp 330, Picoquant) was employed to obtain the fluorescence decay histogram. The decays were recorded time windows of 30–60 ns and analyzed individually with time-resolved fluorescence analysis (EasyTau 2, Picoquant) software based on iterative reconvolution of the data with the instrumental response function (IRF). The full width at half maximum (FWHM) of the IRF was typically on the order of 25 ps.

For the femtosecond fluorescence up-conversion (fs-UC) measurements, an amplified femtosecond double OPA (optical parametric amplifier) 35 fs-laser system was used to provide excitation pulses of 360–400 nm. The power of the excitation beam was set to 300 µW and the fluorescence light emitted from the samples was efficiently collected using a parabolic mirror. The

fluorescence was then filtered using a 420 nm long pass filters to suppress the scattered light, directed, focused and overlapped with a gate pulse (800 nm, ca. 10 μJ) derived from the regenerative amplifier onto an LBO crystal. By tuning the incident angle of these two beams relative to the crystal plane the sum frequency of the fluorescence light and the gate pulse was generated. The time-resolved traces were then recorded by detecting the sum frequency light while changing the relative delay of the gate pulse versus the sample excitation time. Fluorescence gating was done under magic angle conditions in time windows of 6 and 50 ps.

Monochromatic detection was carried out in heterodyne mode and performed using a PMT (R928, Hamamatsu) placed at the second exit of the spectrograph mounted behind a slit. Optical heterodyne detection is a highly sensitive technique that is used to measure very weak changes in absorption induced by a frequency modulated pump beam. An additional bandpass filter 260–380 nm was placed in front of the monochromator to reject excitation light and the second harmonic of the gate pulse. The electrical signal from the photomultiplier tube was gated by a boxcar averager (SR 250, Stanford Research Systems) and detected by a lock-in amplifier (SR830, Stanford Research Systems). The prompt response (or instrumental response function, IRF) of this setup (including laser sources) was determined by detection of scattered light of the excitation pulse under identical condition and found to be approximately 100 fs (FWHM). This value was used in the analysis of all measurements for curve fitting using iterative reconvolution of the data sets while assuming a Gaussian shape for the prompt response.

The samples were prepared in solution and contained in a quartz cuvette. To improve the signal to noise ratio, every measurement was averaged 15 times at 128 delay positions where a delay position is referred to as the time interval between the arrival of the pump and gate pulses at the sample position.

2. Synthesis

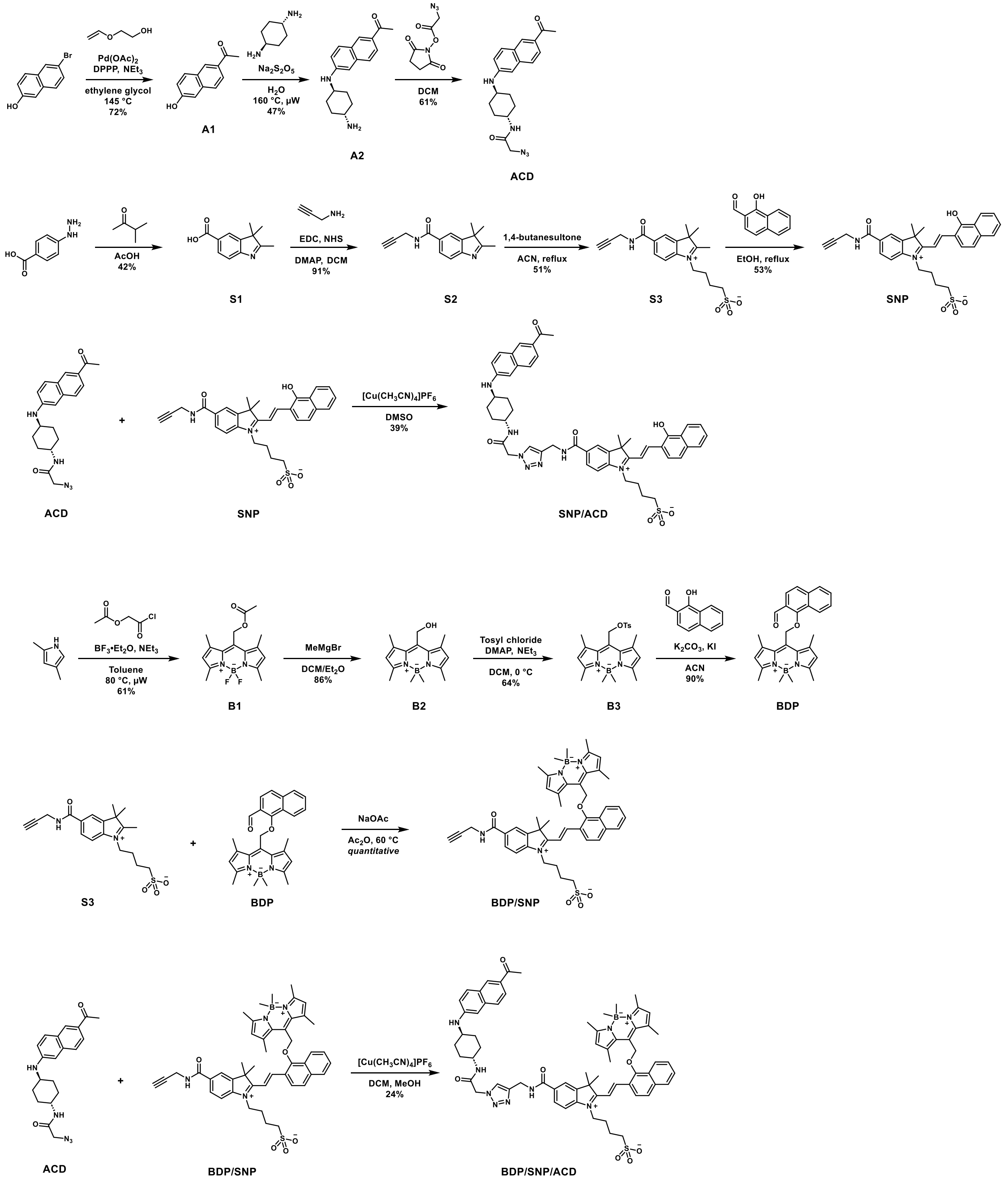

Scheme S1 Synthetic route for the monomer, dyad and triad.

1-(6-Hydroxynaphthalen-2-yl)ethan-1-one (**A1**)

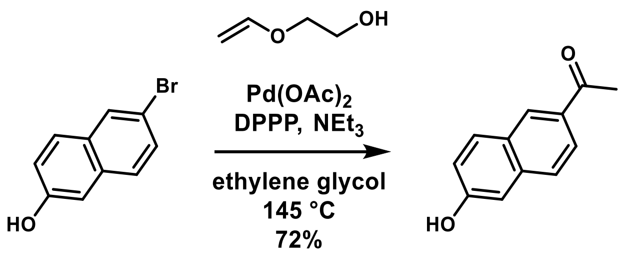

A1

Compound **A1** was prepared according to a modified literature procedure.[7] To the solution of 6-bromo-2-naphthol (2.00 g, 9.0 mmol), $Pd(OAc)_2$ (0.10 g, 0.4 mmol) and DPPP (0.37 g, 0.9 mmol) in degassed ethylene glycol (15 mL) was added hydroxyethyl vinyl ether (2.4 mL, 27 mmol) and NEt_3 (3.1 mL, 22 mmol) at room temperature. The reaction mixture was stirred overnight at 145 °C. Then, the reaction mixture was cooled to room temperature and diluted in DCM (15 mL) and 5% HCl (30 mL) and then stirred for 1 hour at room temperature. The organic layer was collected and washed with H_2O, brine, dried over anhydrous Na_2SO_4, filtered and concentrated under reduced pressure. The residue was purified by silica gel column chromatography (DCM) to afford **A1** (1.2 g, 72%) as a yellowish-green solid.

^{1}H NMR (600 MHz, CD_3OD) δ 8.48 (d, J = 1.2 Hz, 1H), 7.94 – 7.86 (m, 2H), 7.69 (d, J = 8.8 Hz, 1H), 7.17 – 7.13 (m, 2H), 2.68 (s, 3H).

1-(6-(((1r,4r)-4-Aminocyclohexyl)amino)naphthalen-2-yl)ethan-1-one (**A2**)

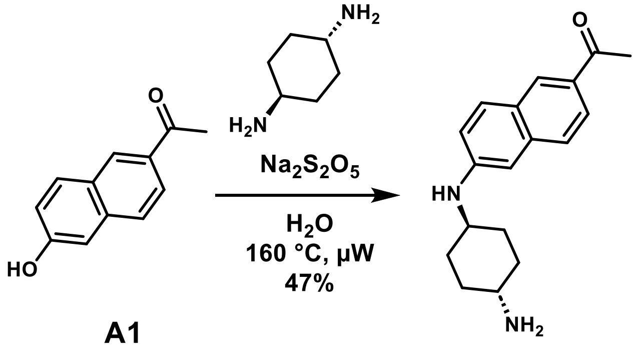

A2

Compound **A2** was prepared according to a modified literature procedure.[8] **A1** (0.5 g, 2.7 mmol), trans-1,6-diaminocyclohexane(1.5 g, 13 mmol), and sodium metabisulfite (1.0 g, 5.4 mmol) were placed in a microwave vial. H_2O (10 mL) was added and the reaction mixture was stirred for 7 hours at 160 °C under microwave irradiation. The reaction mixture was cooled down to room temperature and evaporated under reduced pressure. The residue was dissolved in MeOH, filtered and washed with MeOH. The filtrate was evaporated, and the residue was purified by silica gel

column chromatography (MeOH/DCM = 10 to 50%) to afford **A2** (0.4 g, 47%) as a yellowish-green solid.

^{1}H NMR (600 MHz, CD_3OD) δ 8.34 (d, *J* = 2.1 Hz, 1H), 7.84 (dd, *J* = 8.7, 1.9 Hz, 1H), 7.72 (d, *J* = 8.9 Hz, 1H), 7.57 (d, *J* = 8.7 Hz, 1H), 6.99 (dd, *J* = 8.9, 2.3 Hz, 1H), 6.80 (d, *J* = 2.5 Hz, 1H), 3.46 (tt, *J* = 11.4, 4.0 Hz, 1H), 3.15 (tt, *J* = 11.8, 4.0 Hz, 1H), 2.64 (s, 3H), 2.29 – 2.22 (m, 2H), 2.16 – 2.09 (m, 2H), 1.67 – 1.57 (m, 2H), 1.43 – 1.33 (m, 2H).

N-((1r,4r)-4-((6-Acetylnaphthalen-2-yl)amino)cyclohexyl)-2-azidoacetamide (**ACD**)

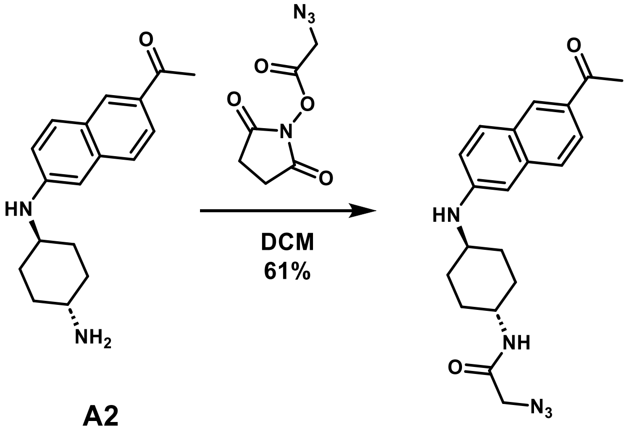

A2

ACD

To the solution of **A2** (0.34 g, 1.2 mmol) in DCM (30 mL) was added the azidoacetic acid NHS ester (0.36 g, 1.8 mmol) and the reaction mixture was stirred at room temperature. Saturated NH_4Cl solution was added to the reaction mixture and extracted with DCM. The combined organic layers were washed with H_2O, brine, dried over anhydrous Na_2SO_4, filtered and concentrated under reduced pressure. The residue was purified by silica gel column chromatography (EtOAc/Pentane = 50% and MeOH/DCM = 2 to 5%) to afford **ACD** (270 mg, 61%) as a yellowish-green solid.

^{1}H NMR (600 MHz, $CDCl_3$) δ 8.03 (s, 1H), 7.59 (dd, *J* = 8.7, 1.9 Hz, 1H), 7.44 (d, *J* = 8.9 Hz, 1H), 7.31 (d, *J* = 8.7 Hz, 1H), 6.69 (dd, *J* = 8.9, 2.3 Hz, 1H), 6.49 (d, *J* = 2.5 Hz, 1H), 3.61 (s, 3H), 3.57 – 3.47 (m, 1H), 3.20 – 3.10 (m, 1H), 2.40 (s, 3H), 2.01 – 1.92 (m, 2H), 1.82 – 1.73 (m, 2H), 1.25 – 1.15 (m, 2H), 1.15 – 1.05 (m, 2H).
^{13}C NMR (151 MHz, $CD_3OD/CDCl_3$) δ 199.0, 167.2, 147.8, 138.2, 130.6, 129.7, 125.5, 125.3, 124.0, 118.67, 103.0, 51.8, 50.4, 46.1, 38.5, 30.9, 30.9, 25.7.
HRMS (ESI), calculated mass for $[M]^+$: 365.18520, found 365.18440; Δ = 2.19 ppm.

2,3,3-Trimethyl-3H-indole-5-carboxylic acid (**S1**)

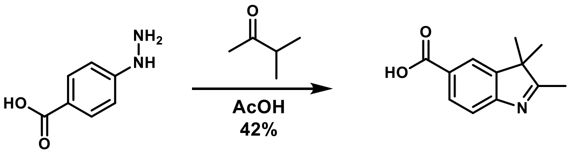

S1

Compound **S1** was prepared according to a modified literature procedure.[9] To the solution of 4-hydrazinebenzoic acid (1.0 g, 6.6 mmol) in glacial acetic acid (10 mL) was added 3-methyl-2-butanone (1.2 mL, 11 mmol) at room temperature and the reaction mixture was refluxed overnight. The acetic acid was evaporated under reduced pressure, and the residue was dissolved in H_2O and the pH was adjusted to 4.0 and then extracted with DCM. The combined organic layers were dried over anhydrous $MgSO_4$, filtered and concentrated under reduced pressure. The residue was purified by silica gel column chromatography (MeOH/DCM = 2 to 10%) to afford **S1** (0.6 g, 42%) as an orange solid.

^{1}H NMR (600 MHz, DMSO-d_6) δ 12.80 (s, 1H), 7.99 (d, J = 1.7 Hz, 1H), 7.91 (dd, J = 8.0, 1.7 Hz, 1H), 7.50 (d, J = 8.1 Hz, 1H), 2.25 (s, 3H), 1.28 (s, 6H).

2,3,3-Trimethyl-N-(prop-2-yn-1-yl)-3H-indole-5-carboxamide (**S2**)

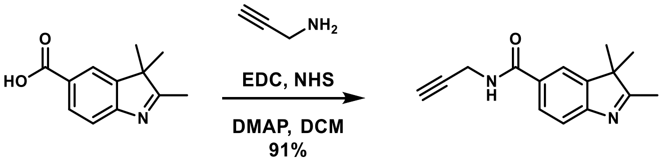

S1 **S2**

To the solution of **S1** (1.0 g, 4.9 mmol) in DCM (50 mL) was added EDC (1.4 g, 7.4 mmol), NHS (0.8 g, 7.4 mmol), and DMAP (0.06 g, 0.5 mmol) at 0 °C and stirred for 4 hours at room temperature. Propargylamine (0.8 mL, 12 mmol) was added and the reaction mixture was stirred overnight at room temperature. Saturated $NaHCO_3$ solution was poured into the reaction mixture and extracted with DCM. The combined organic layers were washed with brine, dried over anhydrous Na_2SO_4, filtered and concentrated under reduced pressure. The residue was purified by silica gel column chromatography (EtOAc/Pentane = 65 to 75%) to afford **S2** (1.1 g, 91%) as an orange solid.

^{1}H NMR (600 MHz, $CDCl_3$) δ 7.80 (d, J = 2.0 Hz, 1H), 7.71 (dd, J = 8.0, 1.9 Hz, 1H), 7.45 (d, J = 8.1 Hz, 1H), 7.00 (t, J = 5.4 Hz, 1H), 4.21 (dd, J = 5.4, 2.5 Hz, 2H), 2.24 (s, 3H), 2.21 (t, J = 2.5 Hz, 1H), 1.24 (s, 6H).
^{13}C NMR (151 MHz, DMSO-d_6) δ 190.7, 166.0, 156.2, 145.9, 130.4, 127.3, 120.9, 118.8, 81.4, 72.8, 53.4, 28.5, 22.4, 15.3.
HRMS (ESI), calculated mass for $[M]^+$: 240.12630, found 240.12529; Δ = 4.21 ppm.

4-(2,3,3-Trimethyl-5-(prop-2-yn-1-ylcarbamoyl)-3H-indol-1-ium-1-yl)butane-1-sulfonate (**S3**)

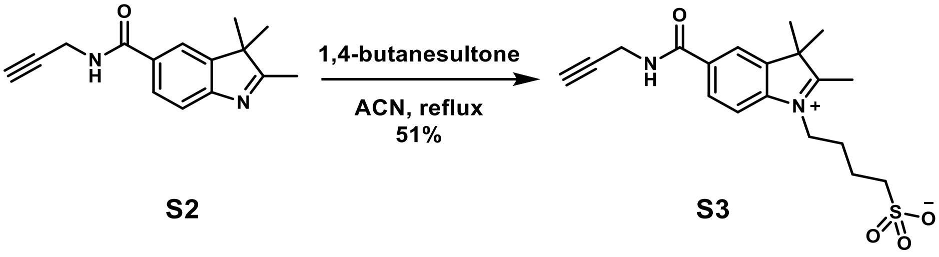

To the solution of **S2** (0.2 g, 0.8 mmol) in ACN (5 mL) was added 1,4-butanesultone (0.1 mL, 1.0 mmol) at room temperature, and the reaction mixture was refluxed overnight. The resulting suspension was dissolved in a minimum amount of MeOH and precipitated into Et_2O. The purple precipitate was filtered and washed with Et_2O. The collected precipitate was purified by reversed-phase silica gel column chromatography (ACN/H_2O = 0 to 90%) to afford **S3** (0.16 g, 51%) as a purple solid.

^{1}H NMR (600 MHz, CD_3OD) δ 8.20 (s, 1H), 8.10 (d, *J* = 8.4 Hz, 1H), 8.05 (d, *J* = 8.5 Hz, 1H), 4.58 (t, *J* = 7.9 Hz, 2H), 4.20 (d, *J* = 2.6 Hz, 2H), 2.90 (t, *J* = 7.1 Hz, 2H), 2.65 (t, *J* = 2.6 Hz, 1H), 2.21 – 2.12 (m, 2H), 2.01 – 1.90 (m, 2H), 1.65 (s, 6H).
^{13}C NMR (151 MHz, DMSO-*d*$_6$) δ 198.7, 165.0, 143.3, 142.0, 134.7, 128.2, 122.7, 115.6, 81.0, 73.2, 54.4, 50.1, 47.6, 28.7, 26.0, 22.1, 22.0, 14.2.
HRMS (ESI), calculated mass for $[M]^+$: 376.14568, found 376.14489; Δ = 2.10 ppm.

(E)-4-(2-(2-(1-Hydroxynaphthalen-2-yl)vinyl)-3,3-dimethyl-5-(prop-2-yn-1-ylcarbamoyl)-3H-indol-1-ium-1-yl)butane-1-sulfonate (**SNP**)

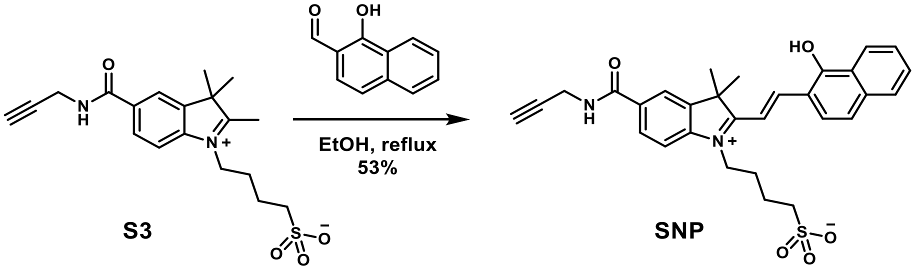

The solution of **S3** (80 mg, 0.21 mmol) and 1-hydroxy-2-naphthaldehyde (48 mg, 0.28 mmol) in EtOH (2 mL) was refluxed overnight and the reaction mixture was precipitated into Et_2O. The resulting red precipitate was filtered and washed with Et_2O. The precipitate was purified by reversed-phase silica gel column chromatography (ACN/H_2O = 0 to 90%) to afford **SNP** (60 mg, 53%) as a red solid.

^{1}H NMR (600 MHz, CD_3OD) δ 9.10 (d, *J* = 16.1 Hz, 1H), 8.35 (d, *J* = 7.4 Hz, 1H), 8.20 (d, *J* = 1.7 Hz, 1H), 8.12 – 8.05 (m, 2H), 7.91 (d, *J* = 8.4 Hz, 1H), 7.86 (d, *J* = 8.0 Hz, 1H), 7.73 (d, *J* = 16.0 Hz, 1H), 7.70 – 7.64 (m, 1H), 7.61 – 7.56 (m, 1H), 7.51 (d, *J* = 8.9 Hz, 1H), 4.68 (t, *J* = 7.8 Hz, 2H), 4.21 (d, *J* = 2.5 Hz, 2H), 2.96 (t, *J* = 7.1 Hz, 2H), 2.66 (t, *J* = 2.5 Hz, 1H), 2.25 – 2.13 (m, 2H), 2.09 – 2.00 (m, 2H), 1.91 (s, 6H).

^{13}**C NMR** (151 MHz, CD_3OD) δ 185.0, 168.2, 160.3, 152.9, 144.9, 139.5, 135.8, 131.4, 130.1, 129.7, 127.8, 126.5, 124.9, 124.7, 123.3, 122.8, 119.1, 115.8, 111.0, 80.6, 72.3, 53.6, 51.3, 47.7, 30.2, 27.9, 27.5, 23.4.
HRMS (ESI), calculated mass for $[M]^+$: 530.18750, found 530.18700; Δ = 0.94 ppm.

4-(5-(((1-(2-(((1r,4r)-4-((6-Acetylnaphthalen-2-yl)amino)cyclohexyl)amino)-2-oxoethyl)-1H-1,2,3-triazol-4-yl)methyl)carbamoyl)-2-((E)-2-(1-hydroxynaphthalen-2-yl)vinyl)-3,3-dimethyl-3H-indol-1-ium-1-yl)butane-1-sulfonate (**SNP/ACD)**

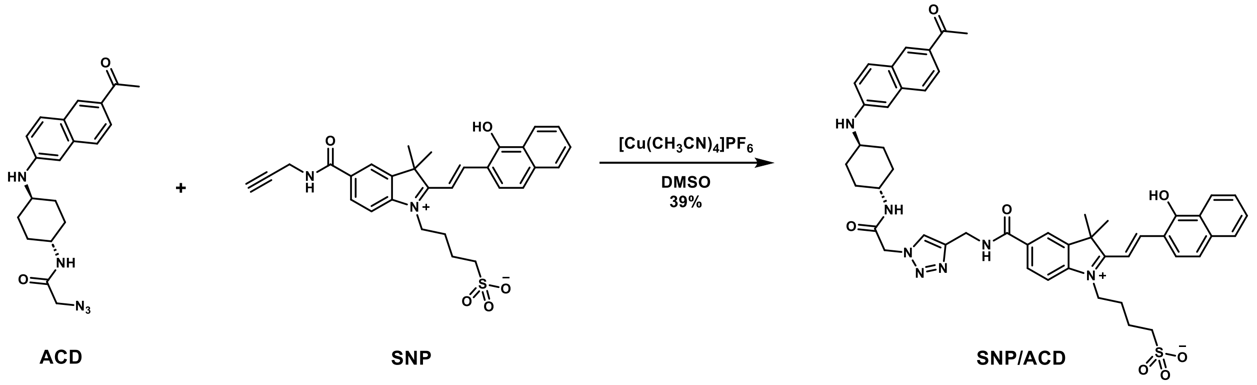

ACD (25 mg, 0.07 mmol) and **SNP** (40 mg, 0.08 mmol) were placed in a microwave vial under nitrogen and dissolved in DMSO (0.8 mL). To this mixture was added $[Cu(CH_3CN)_4]PF_6$ (31 mg, 0.08 mmol) and the reaction mixture was stirred overnight at room temperature. The reaction mixture was purified by preparative C18-HPLC (ACN/H_2O with 0.1% TFA = 20 to 60%) and the resulting crude solid was further purified by silica gel column chromatography (MeOH/DCM = 9 to 20%) to afford **SNP/ACD** (24 mg, 39%) as a purple solid.

^{1}**H NMR** (600 MHz, CD_3OD) δ 8.34 (d, *J* = 2.0 Hz, 1H), 7.96 (s, 1H), 7.82 (dd, *J* = 8.7, 1.9 Hz, 1H), 7.78 (dd, *J* = 8.2, 1.9 Hz, 1H), 7.73 (d, *J* = 8.4 Hz, 1H), 7.71 (d, *J* = 9.2 Hz, 2H), 7.68 (d, *J* = 2.0 Hz, 1H), 7.55 (d, *J* = 8.8 Hz, 1H), 7.38 – 7.31 (m, 2H), 7.27 (ddd, *J* = 8.3, 6.8, 1.3 Hz, 1H), 7.24 (d, *J* = 8.4 Hz, 1H), 7.08 (d, *J* = 10.2 Hz, 1H), 6.98 (dd, *J* = 8.9, 2.4 Hz, 1H), 6.78 (d, *J* = 2.5 Hz, 1H), 6.67 (d, *J* = 8.3 Hz, 1H), 5.88 (d, *J* = 10.2 Hz, 1H), 5.13 (s, 2H), 4.68 (s, 2H), 3.77 – 3.69 (m, 1H), 3.46 – 3.39 (m, 1H), 3.27 – 3.17 (m, 2H), 2.75 – 2.66 (m, 2H), 2.64 (s, 3H), 2.24 – 2.16 (m, 2H), 2.07 – 2.00 (m, 2H), 1.84 – 1.76 (m, 3H), 1.76 – 1.70 (m, 1H), 1.67 – 1.59 (m, 1H), 1.53 – 1.43 (m, 2H), 1.40 – 1.31 (m, 5H), 1.24 (s, 3H).
^{13}**C NMR** (151 MHz, CD_3OD/DMSO-d_6) δ 199.1, 166.5, 151.6, 150.0, 149.8, 139.8, 137.9, 136.0, 131.9, 131.2, 131.2, 129.7, 129.0, 127.8, 126.9, 126.8, 126.6, 126.0, 126.0, 125.6, 125.4, 124.7, 122.4, 122.3, 120.9, 120.3, 119.0, 114.0, 106.9, 103.8, 53.2, 52.7, 52.3, 51.6, 49.7, 44.2, 36.2, 32.3, 32.3, 29.00, 26.9, 26.6, 23.8, 20.7.
HRMS (ESI), calculated mass for $[M]^+$: 895.37270, found 895.37160; Δ = 1.23 ppm.

(5,5-Difluoro-1,3,7,9-tetramethyl-5H-4l4,5l4-dipyrrolo[1,2-c:2',1'-f][1,3,2]diazaborinin-10-yl)methyl acetate (**B1**)

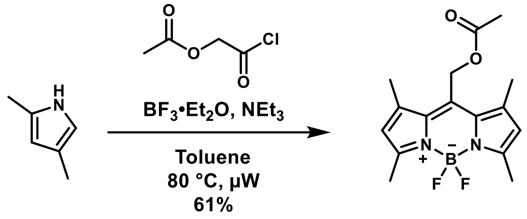

B1

Compound **B1** was prepared according to a modified literature procedure.[10] 2,4-dimethylpyrrole (225 μL, 2.2 mmol), 2-chloro-2-oxoethylacetate (78.5 μL, 0.7 mmol), $BF_3 \cdot Et_2O$ (733 μL, 5.8 mmol), and NEt_3 (407 μL, 2.9 mmol) were placed in a sealed microwave vial under nitrogen and dissolved in toluene (1 mL) at 0 °C. The reaction was stirred for 30 min at 80 °C under microwave irradiation. The toluene was evaporated under reduced pressure. The residue was dissolved in DCM and washed with 1 M HCl, brine, dried over anhydrous Na_2SO_4, filtered and concentrated under reduced pressure. The residue was purified by silica gel column chromatography (EtOAc/Pentane = 10 to 15%) to afford **B1** (142 mg, 61%) as an orange solid.

^{1}H NMR (600 MHz, $CDCl_3$) δ 6.08 (s, 2H), 5.28 (s, 2H), 2.52 (s, 6H), 2.35 (s, 6H), 2.12 (s, 3H).

(1,3,5,5,7,9-Hexamethyl-5H-4l4,5l4-dipyrrolo[1,2-c:2',1'-f][1,3,2]diazaborinin-10-yl)methanol (**B2**)

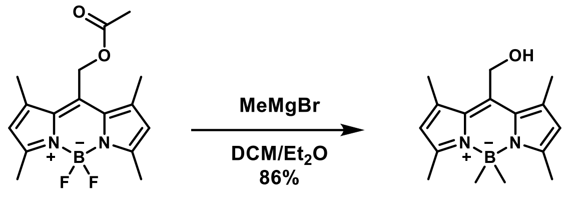

B1 **B2**

Compound **B2** was prepared according to a modified literature procedure.[11] To the solution of **B1** (110 mg, 0.3 mmol) in DCM (10 mL) was added MeMgBr (1 M in Et_2O, 1.7 mL, 5.2 mmol), and the reaction mixture was stirred for 1 hour at room temperature. The reaction was quenched with saturated NH_4Cl solution and EtOAc was added to the mixture. The organic layer was washed with saturated NH_4Cl, H_2O, brine, dried over anhydrous Na_2SO_4, filtered and concentrated under reduced pressure. The residue was purified by silica gel column chromatography (EtOAc/Pentane = 10 to 15%) to afford **B2** (80 mg, 86%) as a red solid.

^{1}H NMR (600 MHz, $CDCl_3$) δ 6.09 (s, 2H), 4.95 (s, 2H), 2.52 (s, 6H), 2.46 (s, 7H), 0.18 (s, 6H).

(1,3,5,5,7,9-Hexamethyl-5H-4l4,5l4-dipyrrolo[1,2-c:2',1'-f][1,3,2]diazaborinin-10-yl)methyl 4-methylbenzenesulfonate (**B3**)

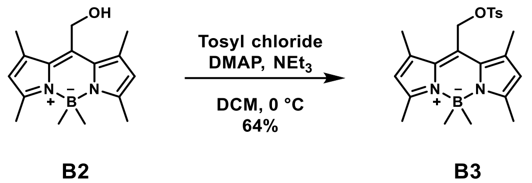

To a solution of **B2** (40 mg, 0.15 mmol) in DCM (3 mL) were added NEt_3 (31 µL, 0.22 mmol), tosyl chloride (42 mg, 0.22 mmol), and DMAP (3.6 mg, 0.03 mmol) at 0 °C, and the reaction mixture was stirred for 30 min at 0 °C. H_2O was added to the reaction mixture and the organic layer was washed with saturated $NaHCO_3$, brine, dried over anhydrous Na_2SO_4, filtered and concentrated under reduced pressure. The residue was purified by silica gel column chromatography (EtOAc/Pentane = 10 to 20%) to afford **B3** (40 mg, 64%) as a red solid.

^{1}H NMR (600 MHz, $CDCl_3$) δ 7.83 (d, J = 8.4 Hz, 2H), 7.34 (d, J = 8.2 Hz, 2H), 6.02 (s, 2H), 5.44 (s, 2H), 2.45 (s, 3H), 2.42 (s, 6H), 2.25 (s, 6H), 0.14 (s, 6H).
^{13}C NMR (151 MHz, $CDCl_3$) δ 153.9, 145.5, 137.6, 133.0, 131.3, 130.6, 130.0, 128.3, 123.1, 62.9, 21.8, 16.8, 16.1.
HRMS (ESI), calculated mass for [M]+: 424.19920, found 424.19834; Δ = 2.03 ppm.

1-((1,3,5,5,7,9-Hexamethyl-5H-4l4,5l4-dipyrrolo[1,2-c:2',1'-f][1,3,2]diazaborinin-10-yl)methoxy)-2-naphthaldehyde (**BDP**)

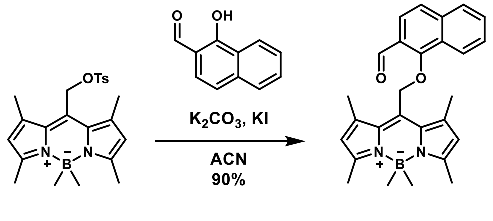

To a solution of **B3** (100 mg, 0.24 mmol) in ACN (3 mL) was added 1-hydroxy-2-naphthaldehyde (43 mg, 0.25 mmol), K_2CO_3 (39 mg, 0.28 mmol), and KI (7.8 mg, 0.05 mmol), and the reaction mixture was stirred overnight at room temperature. The reaction mixture was filtered and washed with DCM. The collected filtrate was concentrated under reduced pressure. The residue was purified by silica gel column chromatography (DCM/Pentane = 0 to 50%) to afford **BDP** (90 mg, 90%) as a red solid.

^{1}H NMR (600 MHz, $CDCl_3$) δ 10.19 (s, 1H), 8.20 (d, J = 8.4 Hz, 1H), 7.86 (d, J = 8.2 Hz, 1H), 7.79 (d, J = 8.6 Hz, 1H), 7.65 (d, J = 8.5 Hz, 1H), 7.62 (ddd, J = 8.2, 6.8, 1.3 Hz, 1H), 7.54 (ddd, J = 8.3, 6.9, 1.4 Hz, 1H), 5.96 (s, 2H), 5.73 (s, 2H), 2.45 (s, 6H), 2.07 (s, 6H), 0.20 (s, 6H).
^{13}C NMR (151 MHz, $CDCl_3$) δ 189.8, 160.6, 153.5, 137.9, 137.3, 134.2, 130.8, 129.5, 128.5, 128.4, 127.1, 126.0, 125.1, 123.8, 123.3, 122.9, 69.4, 16.8, 16.2.
HRMS (ESI), calculated mass for [M]+: 424.23220, found 424.23170; Δ = 1.18 ppm.

(E)-4-(2-(2-(1-((1,3,5,5,7,9-Hexamethyl-5H-4l4,5l4-dipyrrolo[1,2-c:2',1'-f][1,3,2]diazaborinin-10-yl)methoxy)naphthalen-2-yl)vinyl)-3,3-dimethyl-5-(prop-2-yn-1-ylcarbamoyl)-3H-indol-1-ium-1-yl)butane-1-sulfonate (**BDP/SNP**)

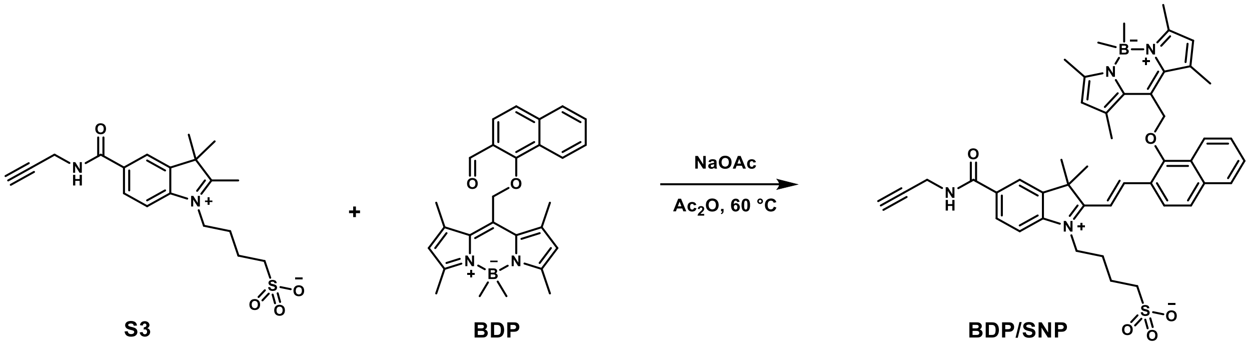

Compound **S3** (51 mg, 0.14 mmol), **BDP** (69 mg, 0.16 mmol), and NaOAc (17 mg, 0.20 mmol) were placed in a microwave vial at room temperature and dissolved in Ac_2O (1 mL). The reaction mixture was stirred for 1 hour at 60 °C and then the solvent was evaporated under reduced pressure. The residue was triturated in Et_2O three times to afford **BDP/SNP** (106 mg, quantitative) as a red solid. The product was used without further purification.

^{1}HNMR (600 MHz, CD_3OD) δ 8.70 (d, J = 16.3 Hz, 1H), 8.30 (d, J = 8.3 Hz, 1H), 8.22 (d, J = 8.8 Hz, 1H), 8.11 – 8.06 (m, 2H), 7.99 (d, J = 8.6 Hz, 1H), 7.96 (d, J = 9.1 Hz, 1H), 7.85 (d, J = 8.8 Hz, 1H), 7.76 – 7.63 (m, 3H), 6.10 (s, 2H), 5.92 (s, 2H), 4.59 (t, J = 8.0 Hz, 2H), 4.21 (d, J = 2.5 Hz, 2H), 2.91 (t, J = 7.0 Hz, 2H), 2.66 (t, J = 3.0 Hz, 1H), 2.42 (s, 6H), 2.15 (s, 5H), 2.13 – 2.06 (m, 2H), 2.03 – 1.94 (m, 2H), 1.51 (s, 6H), 0.11 (s, 6H).
^{13}CNMR (151 MHz, CD_3OD) δ 185.3, 168.1, 160.8, 154.9, 151.4, 145.0, 144.6, 139.5, 139.2, 136.5, 135.9, 132.1, 131.1, 130.2, 130.0, 128.7, 127.0, 125.5, 125.5, 124.5, 124.3, 123.1, 116.5, 114.1, 80.5, 72.4, 71.0, 54.0, 51.1, 48.2, 30.2, 28.0, 26.7, 23.3, 16.9, 16.1.
HRMS (ESI), calculated mass for [M]+: 782.36732, found 782.36627; Δ = 1.34 ppm.

4-(5-(((1-(2-(((1r,4r)-4-((6-Acetylnaphthalen-2-yl)amino)cyclohexyl)amino)-2-oxoethyl)-1H-1,2,3-triazol-4-yl)methyl)carbamoyl)-2-((E)-2-(1-((1,3,5,5,7,9-hexamethyl-5H-4l4,5l4-dipyrrolo[1,2-c:2',1'-f][1,3,2]diazaborinin-10-yl)methoxy)naphthalen-2-yl)vinyl)-3,3-dimethyl-3H-indol-1-ium-1-yl)butane-1-sulfonate (**BDP/SNP/ACD**)

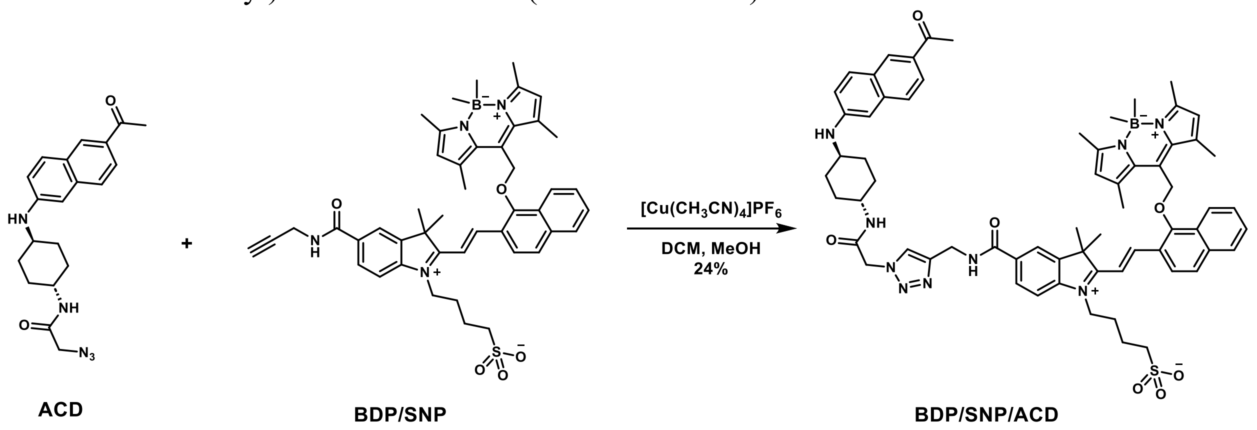

Compound **ACD** (9.3 mg, 26 µmol) and **BDP/SNP** (20 mg, 26 µmol) were placed in a microwave vial under nitrogen and dissolved in DCM (0.4 mL) and MeOH (0.1 mL). To this mixture was added $[Cu(CH_3CN)_4]PF_6$ (1.9 mg, 5.1 µmol) and the reaction mixture was stirred for 1 hour at room temperature. The solvent was evaporated under reduced pressure and the residue was purified by preparative C18-HPLC (ACN/H_2O with 0.1% TFA = 40 to 95%) to afford **BDP/SNP/ACD** (7 mg, 24%) as a red solid.

^{1}H NMR (600 MHz, DMSO-d_6) δ 9.29 (t, J = 5.7 Hz, 1H), 8.52 (d, J = 16.2 Hz, 1H), 8.45 (d, J = 9.0 Hz, 1H), 8.36 – 8.32 (m, 2H), 8.21 (d, J = 8.4 Hz, 1H), 8.18 (s, 1H), 8.11 (s, 2H), 8.07 (d, J = 8.2 Hz, 1H), 7.97 (s, 1H), 7.91 (d, J = 8.8 Hz, 1H), 7.83 (d, J = 16.3 Hz, 1H), 7.77 – 7.71 (m, 3H), 7.63 (t, J = 7.7 Hz, 1H), 7.58 (d, J = 8.8 Hz, 1H), 7.02 (dd, J = 8.9, 2.3 Hz, 1H), 6.79 (d, J = 2.2 Hz, 1H), 6.25 (d, J = 7.8 Hz, 1H), 6.16 (s, 2H), 5.85 (s, 2H), 5.06 (s, 2H), 4.68 (t, J = 8.0 Hz, 2H), 4.59 (d, J = 5.6 Hz, 2H), 3.63 – 3.53 (m, 1H), 3.40 – 3.37 (m, 1H), 2.58 (s, 3H), 2.54 (t, J = 7.1 Hz, 2H), 2.39 (s, 6H), 2.15 – 2.09 (m, 6H), 2.09 – 2.05 (m, 2H), 1.98 – 1.92 (m, 2H), 1.89 (d, J = 12.1 Hz, 2H), 1.85 – 1.79 (m, 2H), 1.47 (s, 6H), 1.45 – 1.36 (m, 2H), 1.32 – 1.22 (m, 2H), 0.09 (s, 6H).
^{13}C NMR (151 MHz, DMSO-d_6) δ 196.8, 182.7, 165.1, 164.5, 163.0, 158.3, 153.0, 148.1, 147.4, 144.5, 143.4, 142.9, 137.9, 137.3, 137.1, 134.9, 134.5, 130.5, 130.4, 130.2, 129.7, 129.5, 128.7, 128.5, 127.4, 127.0, 125.4, 125.3, 124.7, 124.5, 124.0, 123.8, 123.4, 123.3, 121.9, 119.0, 115.5, 113.8, 102.2, 69.5, 52.0, 51.6, 50.1, 49.9, 47.9, 46.9, 35.0, 30.9, 30.8, 27.0, 26.3, 25.6, 22.2, 16.3, 15.4.
HRMS (ESI), calculated mass for [M]+: 1147.55250, found 1147.55000; Δ = 2.18 ppm.

3. Optical spectroscopy

3a. Absorption spectra of SNP_C and SNP_{CL}

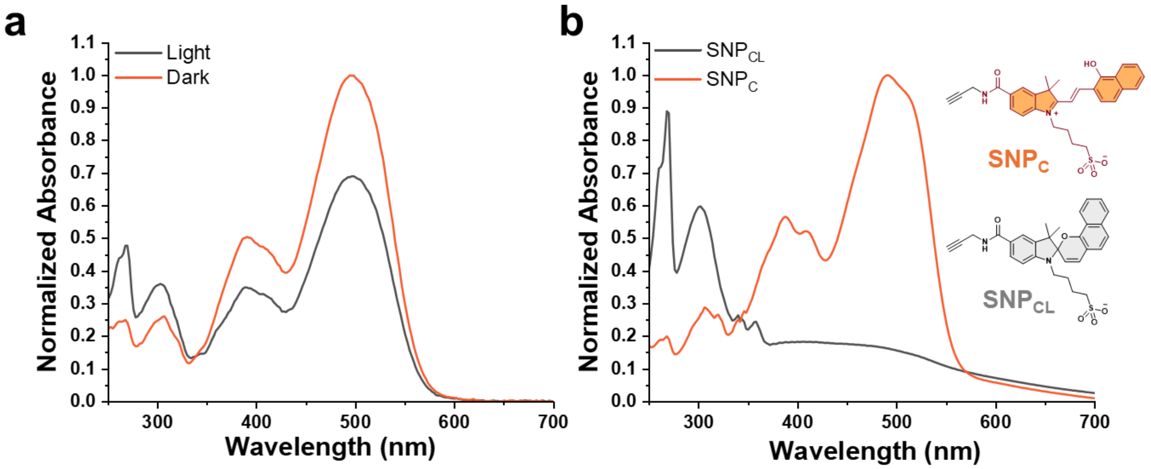

Figure S1. Absorption spectra of **SNP** monomer. (a) Room-temperature absorption spectra of **SNP** in MeOH containing 1 mM HCl before (orange line) and after (black line) irradiation at 405 nm at room temperature. (b) Absorption spectra of the $\mathbf{SNP_C}$ (orange line) and $\mathbf{SNP_{CL}}$ (black line) isomers in MeOH containing 1 mM HCl at 190 K. The $\mathbf{SNP_{CL}}$ spectrum was recorded after irradiation at 405 nm. Minor baseline fluctuations likely arise from condensation or partial freezing of residual water or DMSO in the solvent under cryogenic conditions.

3b. Fluorescence background emission upon HCl addition

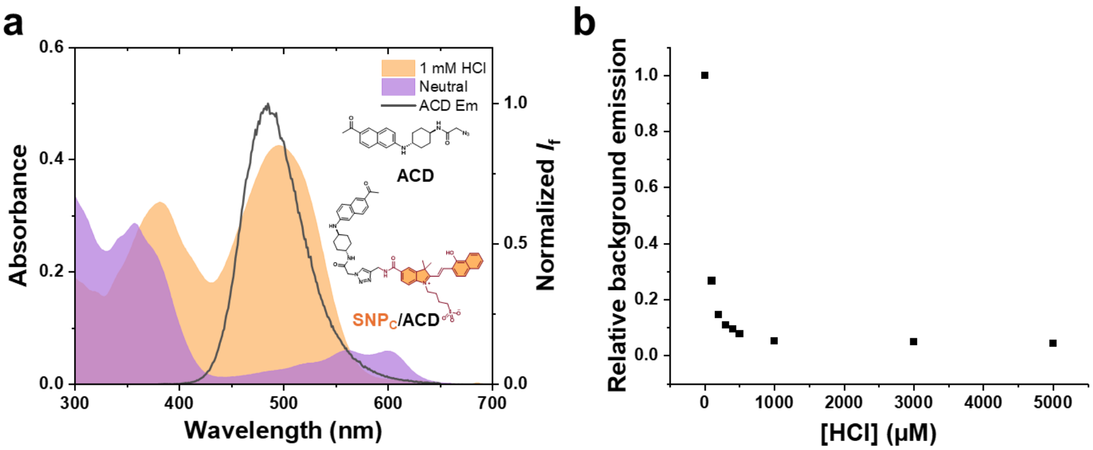

Figure S2. Effect of HCl on background emission. (a) Absorption (filled area) and emission (solid line, excitation at 365 nm) spectra of **SNP** in MeOH with (orange) or without (purple) 1 mM HCl and of **ACD** (black) in MeOH. (b) Relative emission intensity of **SNP/ACD** dyad compared to **ACD** monomer at varying HCl concentrations.

3c. Chemical and photostability

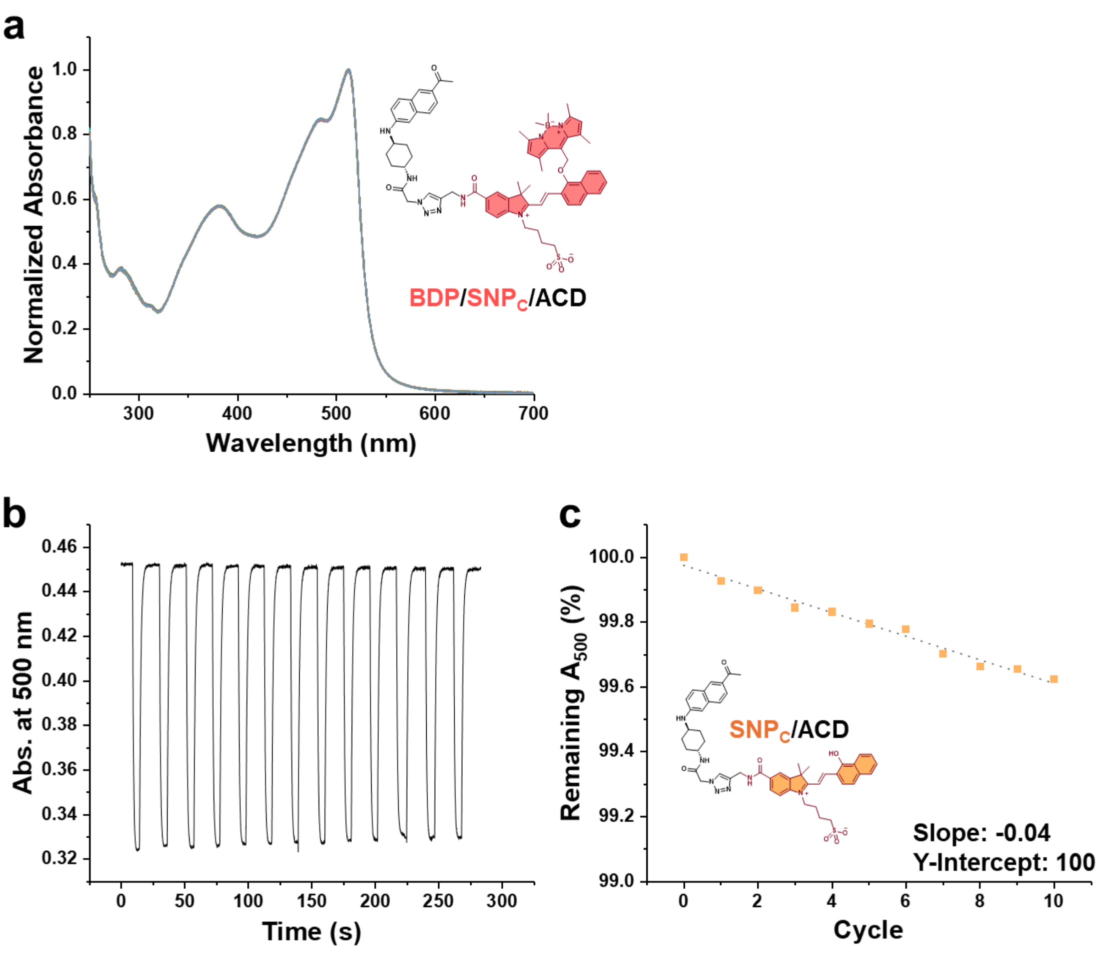

Figure S3. Chemical and photostability. (a) Absorption spectra of **BDP/SNP$_C$/ACD** triad in MeOH with 1 mM HCl recorded over 1 hour. (b) Photostability of **SNP$_C$/ACD** dyad monitored over 10 consecutive cycles of photoisomerization under 405 nm excitation, followed by thermal back isomerization. (c) Absorbance of **SNP$_C$/ACD** dyad at 500 nm after each cycle.

3d. BE compensation for 2for1 performance validation in SNP_C/ACD

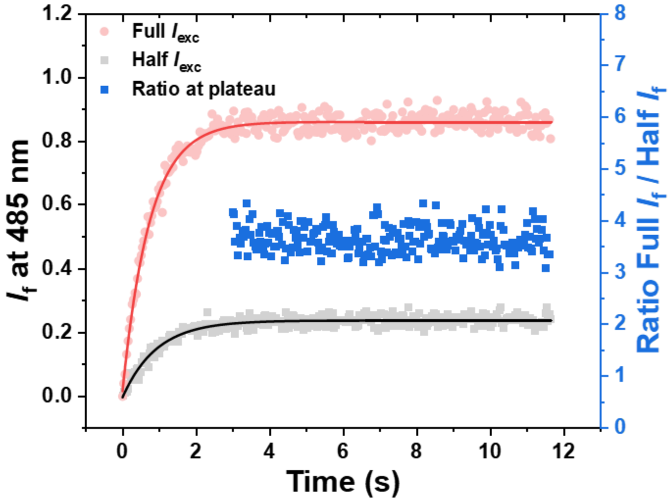

Figure S4. Exponential fitting of fluorescence rise kinetics for the **SNP_C/ACD** dyad under 375 nm irradiation at Full (red) and Half (black) excitation intensities after subtraction of the background emission. Blue points represent the emission intensities ratio at Full/Half excitation intensity at the plateau.

3e. Behavior of monomer fluorophore ACD in 1PE

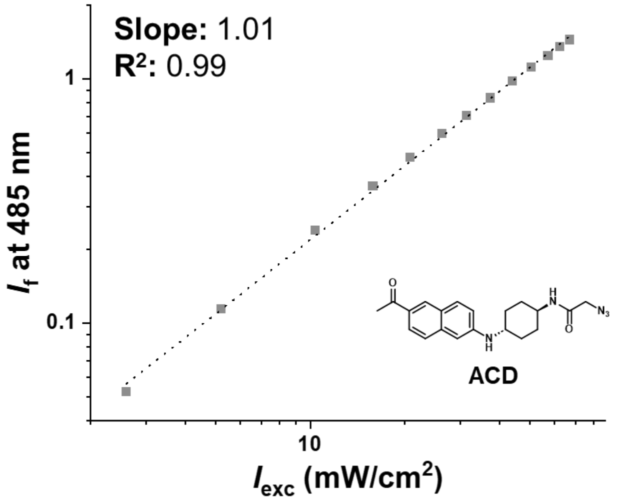

Figure S5. Log–log plot of fluorescence intensity I_f at 485 nm versus excitation intensity I_{exc} for the **ACD** monomer under 375 nm excitation. The slope of 1.01 clearly shows that excitation for fluorescence readout is in the 1PE regime.

4. Kinetic modeling

4a. Modeling of SNP_C/ACD 2for1 behavior

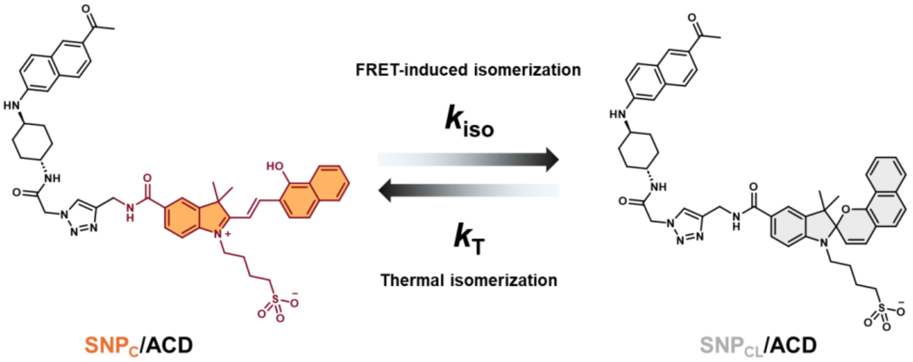

Figure S6. Photo- and thermal isomerization scheme of the **SNP** photoswitch in the **SNP/ACD** dyad, illustrating the 2for1 behavior.

The photoinduced isomerization rate k_{iso} was determined by monitoring the time dependent fluorescence change until the photothermal equilibrium was reached under various excitation intensities and fitting the resulting kinetics to extract the observed rate constant k_{obs}. The respective observed rate constant reflects the sum of the photoinduced isomerization rate and the thermal isomerization rate. The photoinduced isomerization rate is directly proportional to the excitation light absorption by the **SNP_C/ACD** dyad. Therefore,

$$k_{iso} = aI_{exc}$$

$$k_{obs} = k_{iso} + k_T = aI_{exc} + k_T$$

where a is a proportionality constant.

By performing measurements of k_{obs} under multiple excitation intensities and plotting k_{obs} against I_{exc}, 0.8 s^{-1} of thermal isomerization rate k_T was obtained as the y-intercept (Figure S7). From exponential fits of the **SNP/ACD** fluorescence rise under Full I_{exc} (39 mW/cm^2) and Half I_{exc} (78 mW/cm^2), k_{obs} were determined to be 1.4 and 1.1 s^{-1}, respectively (Figure S8). This corresponds to k_{iso} values of 0.6 and 0.3 s^{-1}, illustrating the direct proportionality between photoinduced isomerization rate and excitation intensity, as expected for the designed 2for1 function.

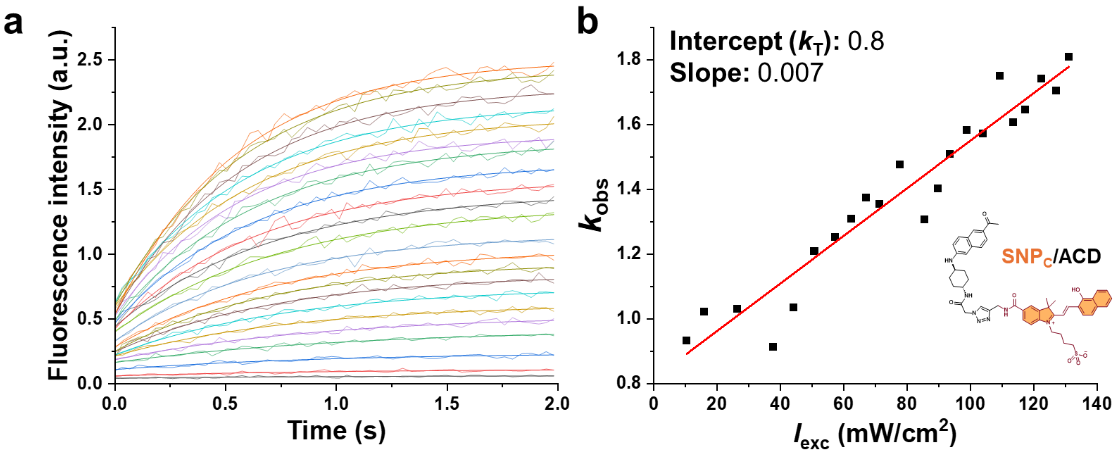

Figure S7. Determination of the thermal isomerization rate constant (k_T) in **SNP/ACD** from fluorescence rise kinetics. (a) Fluorescence rise of **SNP/ACD** at 485 nm under different 375 nm laser excitation intensities together with the mono-exponential fits. (b) Observed rate constant (k_{obs}) plotted versus the excitation intensity (I_{exc}).

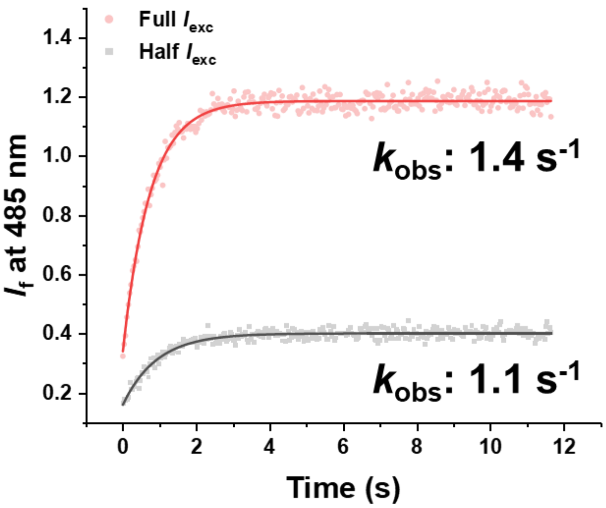

Figure S8. Exponential fitting of fluorescence rise kinetics of **SNP/ACD** under Full (red) and Half (black) I_{exc}.

Although the photoinduced isomerization rate increased exactly by a factor of two upon doubling the excitation intensity, as initially designed, the fluorescence plateau did not exhibit the pure quadratic dependence with a factor of 4 (2^2) expected for ideal two-photon excitation in Figure 3. This discrepancy suggests the presence of additional factors influencing the observed behavior. To investigate this deviation, the 2for1 performance was modeled using a kinetic scheme that contains both photo- and thermal isomerization pathways.

At photothermal equilibrium, the fraction of **SNP$_C$/ACD** converted to **SNP$_{CL}$/ACD** is determined

by the ratio of the relevant kinetic constants:

$$[\mathbf{SNP_{CL}/ACD}] = c\frac{k_{\text{iso}}}{k_{\text{iso}}+k_{\text{T}}} = c\frac{aI_{\text{exc}}}{aI_{\text{exc}}+k_{\text{T}}}$$

where c is the total concentration of the dyad.

In this model, the fluorescence intensity at equilibrium depends on both the concentration of the fluorescent **SNP$_{\mathbf{CL}}$/ACD** isomer and the excitation intensity. Therefore, the overall fluorescence intensity, I_f can be expressed as a function of excitation intensity and the thermal isomerization rate, with proportionality factors *a* and *b*, as follows:

$$I_f = b[\mathbf{SNP_{CL}/ACD}]I_{\text{exc}} = bc\frac{aI_{\text{exc}}{}^2}{aI_{\text{exc}}+k_{\text{T}}}$$

The model predicts that a quadratic dependence of the fluorescence intensity I_f with excitation intensity I_{exc} is attainable when the rate of photoinduced isomerization **SNP$_{\mathbf{C}}$/ACD** → **SNP$_{\mathbf{CL}}$/ACD** (aI_{exc}) is much slower than the thermal isomerization rate **SNP$_{\mathbf{CL}}$/ACD** → **SNP$_{\mathbf{C}}$/ACD** (k_T), that is, when aI_{exc} in the denominator is negligible. However, experimental measurements of **SNP$_{\mathbf{C}}$/ACD** in this regime (I_{exc} = 4 – 366 mW/cm^2) revealed a maximum non-linearity factor (NLF) of 1.53 in the log-log plot of fluorescence versus excitation intensity, not the expected theoretical maximum of 2 (Figure S9 and S11).

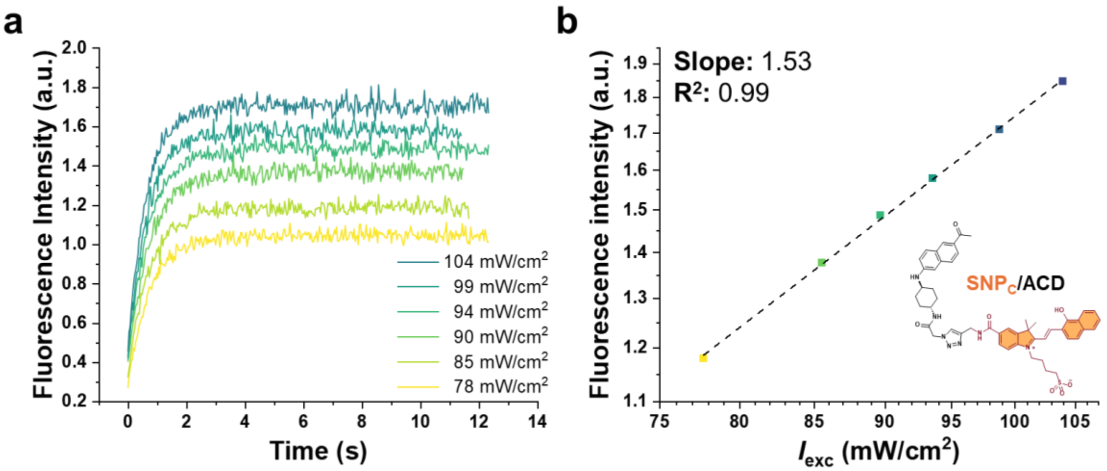

Figure S9. Determination of the nonlinearity factor (NLF) of the **SNP/ACD** dyad. (a) Fluorescence intensities of **SNP/ACD** at 485 nm upon 375 nm excitation at various excitation intensities. (b) The corresponding log-log plot.

To identify the source of this deviation, we introduced an additional term to account for background emission (BE), that is, fluorescence intensity observed prior to photoisomerization to the fluorescent isomeric form (**SNP$_{\mathbf{C}}$/ACD** → **SNP$_{\mathbf{CL}}$/ACD**). The BE was determined to approximately 7% of the maximum attainable fluorescence signal, estimated by comparing the

steady-state emission of **SNP/ACD** to the emission of the **ACD** monomer (Figure S10).

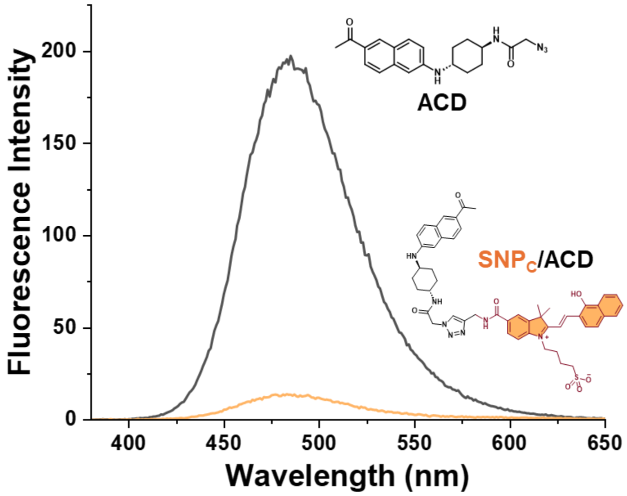

Figure S10. Fluorescence emission of **SNP/ACD** compared to **ACD** upon 375 nm excitation.

Since this BE contribution (X in equation below) scales linearly with the excitation intensity, it was incorporated into the model as:

$$I_\mathrm{f} = bc((1-X)[\mathbf{SNP_{CL}/ACD}] + X)I_\mathrm{exc}$$
$$= bc\left((1-X)\frac{aI_\mathrm{exc}{}^2}{aI_\mathrm{exc}+k_\mathrm{T}} + XI_\mathrm{exc}\right), (0 \le X \le 1) \qquad \text{Equation 1.}$$

From Equation 1, it is seen that the overall emission intensity I_f has two contributions:

1) The contribution from BE = $bcXI_\mathrm{exc}$. This contribution scales linearly with the excitation intensity I_exc.
2) The contribution from photoisomerization to the fluorescent isomer = $bc(1-X)\frac{aI_\mathrm{exc}{}^2}{aI_\mathrm{exc}+k_\mathrm{T}}$. This contribution displays a quadratic dependence on the excitation intensity I_exc when k_T dominates over aI_exc, that is, when the thermal isomerization rate is substantially faster than the rate of photoisomerization.

The BE dominates at low excitation intensities, when the quadratic contribution from photoisomerization to the fluorescent isomer is low. Thus, the non-linearity factor NLF is expected to be close to 1 at low excitation intensities (aI_exc is close to zero). Furthermore, at very high excitation intensities (aI_exc is substantially larger than k_T), isomerization to the fluorescent isomer **SNP$_\mathbf{CL}$/ACD** is virtually quantitative and NLF is approaching 1 again. Thus, a plot of NLF versus I_exc is expected to start around NLF=1 at low I_exc-values, pass through a maximum at intermediate I_exc-values, and decay back to NLF=1 again at high I_exc-values.

Specifically, with a 7% BE contribution, the model predicts a maximum slope of 1.58, which is to be compared to the experimentally obtained value of 1.53 (see Figure S11). Please note that when modeling the kinetic situation, we are not using any free parameters, but instead all parameters are fixed using experimentally obtained data.

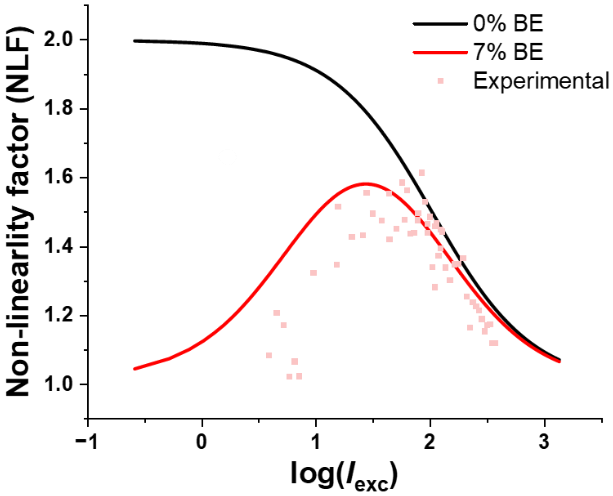

Figure S11. Simulated (solid lines) and experimental (dots) results showing the influence of background emission (BE) on the 2for1 performance of **SNP_C/ACD**. Proportionality factor *a* was determined to be 0.007 from the relationship between k_{iso} and $I_{exc.}$ The values of parameters *b* and *c* do not influence the fit since they cancel out in the evaluation.

4b. Modeling of BDP/SNP$_C$/ACD 3for1 behavior

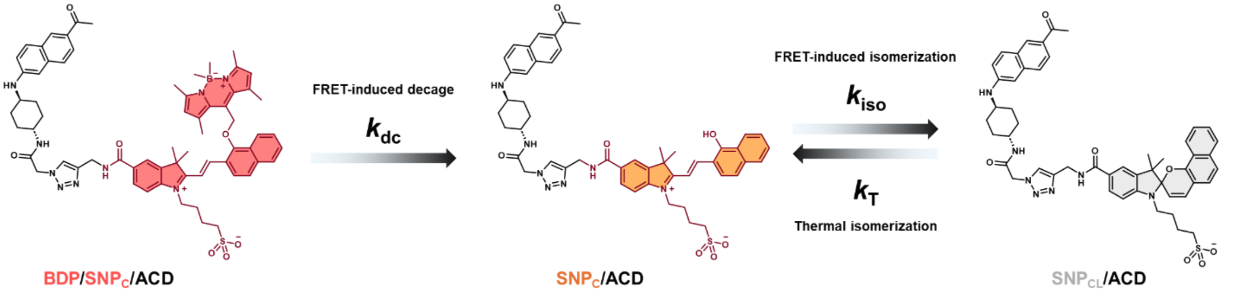

Figure S12. Kinetic scheme of the photodecaging of **BDP** and the subsequent photo- and thermal-isomerization process of the **SNP** photoswitch within the **BDP/SNP$_C$/ACD** triad, demonstrating 3for1 behavior.

Analogous to the kinetic scheme used for the 2for1 model, a 3for1 kinetic model was developed by evaluating the relationship between I_f and I_{exc}. To accurately describe the 3for1 behavior, the model must include the concentration of each isomer and the BE originating from each isomer. Although the 3for1 molecule was designed to exhibit negligible emission from the first two isomers **BDP/SNP$_C$/ACD** and **SNP$_C$/ACD**, experimental observations already revealed a 7% background from **SNP$_C$/ACD**. It is therefore reasonable to anticipate that **BDP/SNP$_C$/ACD** may also exhibit a small, but non-zero, fluorescence contribution (X in equation below). Incorporating these BE components, the total fluorescence intensity can be expressed with proportionality factor b as:

$$I_f = (X[\mathbf{BDP/SNP_C/ACD}] + 0.07[\mathbf{SNP_C/ACD}] + [\mathbf{SNP_{CL}/ACD}])bI_{exc}, \quad (0 \leq X \leq 1)$$

The equilibrium concentrations of each isomer were calculated using established kinetic schemes as described in the literature.[12]

$$[\mathbf{BDP/SNP_C/ACD}] = a\left(\frac{k_{dc}(k_{iso} + k_T - \gamma_1)}{\gamma_1(\gamma_2 - \gamma_1)}e^{-\gamma_1 t} + \frac{k_{dc}(\gamma_2 - k_{iso} - k_T)}{\gamma_2(\gamma_2 - \gamma_1)}e^{-\gamma_2 t}\right)$$

$$[\mathbf{SNP_C/ACD}] = ak_{dc}\left(\frac{k_T}{\gamma_1\gamma_2} + \frac{k_T - \gamma_1}{\gamma_1(\gamma_1 - \gamma_2)}e^{-\gamma_1 t} + \frac{k_T - \gamma_2}{\gamma_2(\gamma_2 - \gamma_1)}e^{-\gamma_2 t}\right)$$

$$[\mathbf{SNP_{CL}/ACD}] = ak_{dc}k_{iso}\left(\frac{1}{\gamma_1\gamma_2} + \frac{1}{\gamma_1(\gamma_1 - \gamma_2)}e^{-\gamma_1 t} - \frac{1}{\gamma_2(\gamma_1 - \gamma_2)}e^{-\gamma_2 t}\right)$$

where $[\mathbf{BDP/SNP_C/ACD}]_0 = a$, $\gamma_1\gamma_2 = k_{dc}(k_{iso} + k_T)$, and $\gamma_1 + \gamma_2 = k_{dc} + k_{iso} + k_T$.

To obtain the BE-value from **BDP/SNP$_C$/ACD**, steady-state fluorescence measurements were performed, comparing its emission intensity with that of **ACD** (Figure S13a). This analysis yielded a 1% contribution. k_{dc} can be determined from the bi-exponential fitting of the fluorescence rise shown in Figure 4 in the main text. The equation for the 3for1 simulation consists of a bi-exponential function. Given that the slower component of the rise in fluorescence intensity corresponds to the rate constant of decaging k_{dc}, the extracted values of 0.0088 s^{-1} and 0.0046 s^{-1}

were determined as k_{dc} under Full (80 mW/cm^2) and Half I_{exc} (160 mW/cm^2), respectively (Figure S13b). For k_{iso} determination, experiments were conducted under identical conditions to those used for the data in Figure 4, ensuring complete experimental consistency (Figure S13c). The resulting values were 1.4 and 0.7 s^{-1} under Full and Half I_{exc}, respectively. These kinetic parameters were used to simulate the overall 3for1 behavior of the **BDP/SNP$_C$/ACD** triad in Figure 4b in the manuscript. No free parameters were used in the fitting procedure, but all were fixed according to the experimentally determined value.

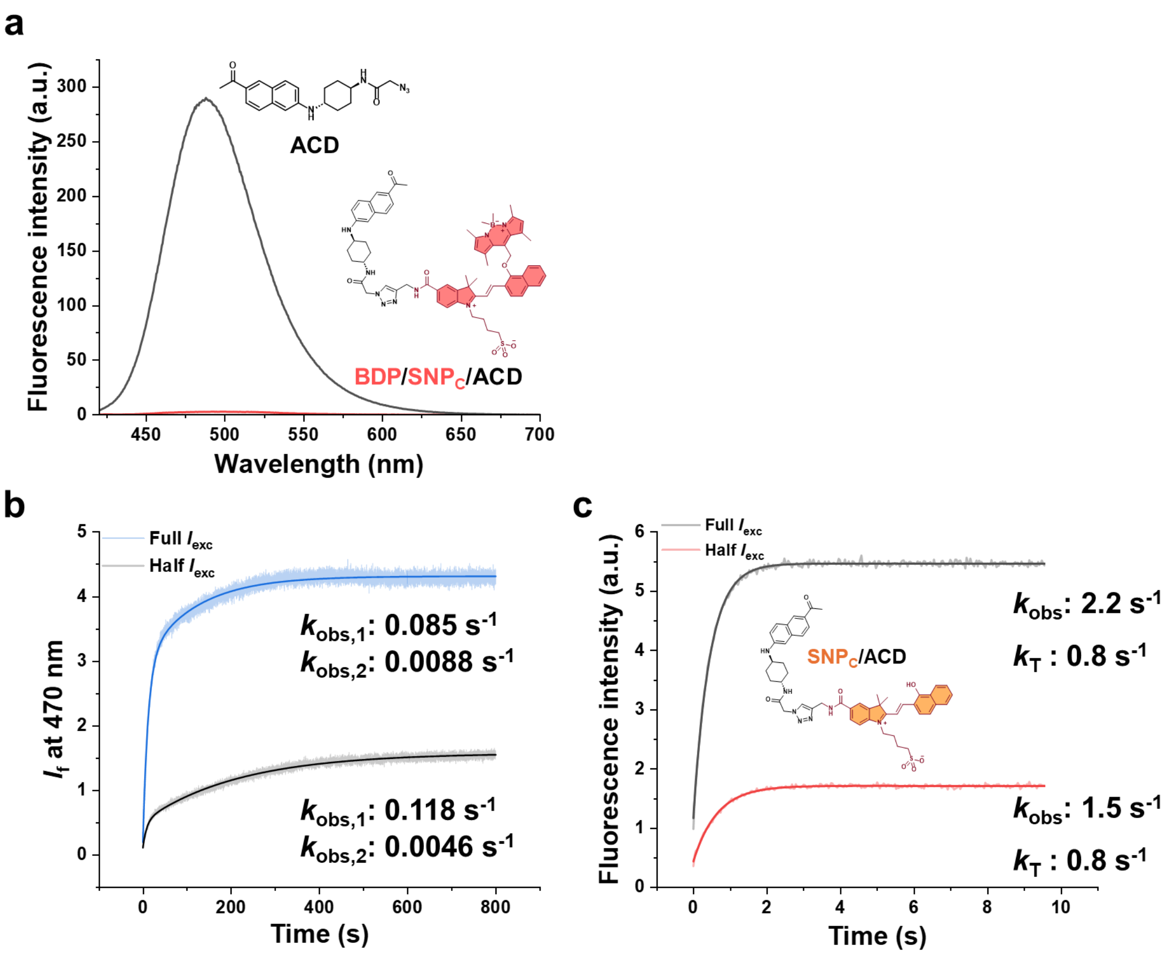

Figure S13. Determination of parameters used for 3for1 modeling. (a) Fluorescence background of **BDP/SNP$_C$/ACD** compared to **ACD** upon 405 nm excitation. (b) Bi-exponential fitting of fluorescence rise kinetics in **BDP/SNP$_C$/ACD**. (c) Exponential fitting of fluorescence rise kinetics in **SNP$_C$/ACD** under identical conditions as in Figure 4.

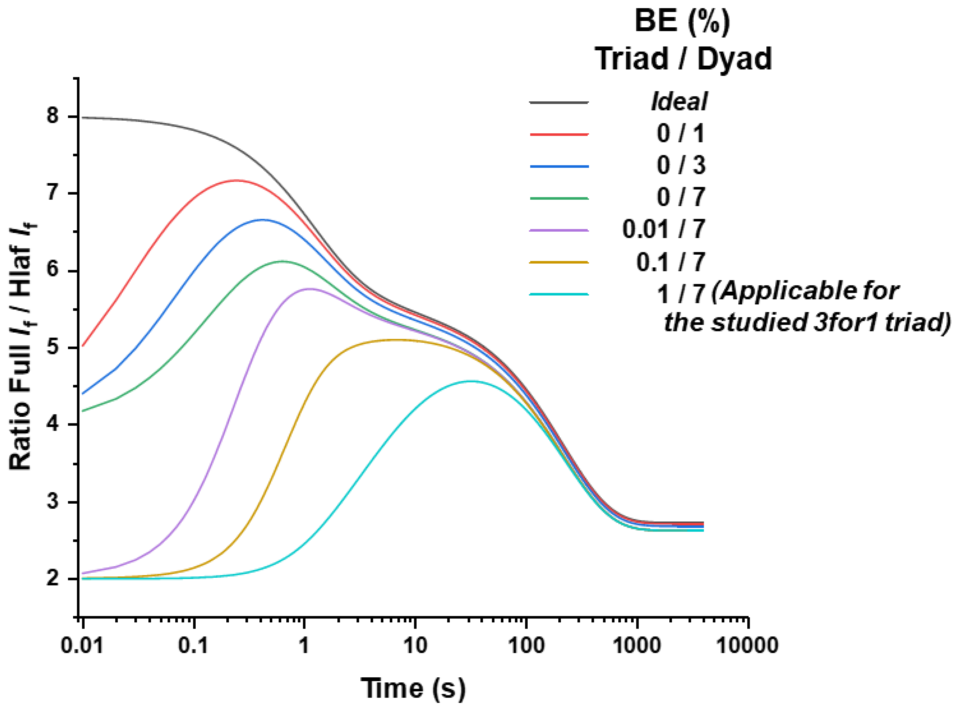

Figure S14. Kinetic simulations showing the impact of BE on the nonlinear performance of the **BDP/SNP$_C$/ACD** triad. The simulated traces demonstrate the fluorescence ratio under varying BE from both the triad and the dyad. It is clearly seen that the initial (t=0) NLF approaches 3.0, corresponding to a ratio of 8.0, as the BE is gradually decreased. The cyan line corresponding to 1% and 7% of BE (triad and dyad, respectively) is applicable for the herein studied 3for1 triad.

5. Theoretical calculations

5a. Optimized structure at ground state

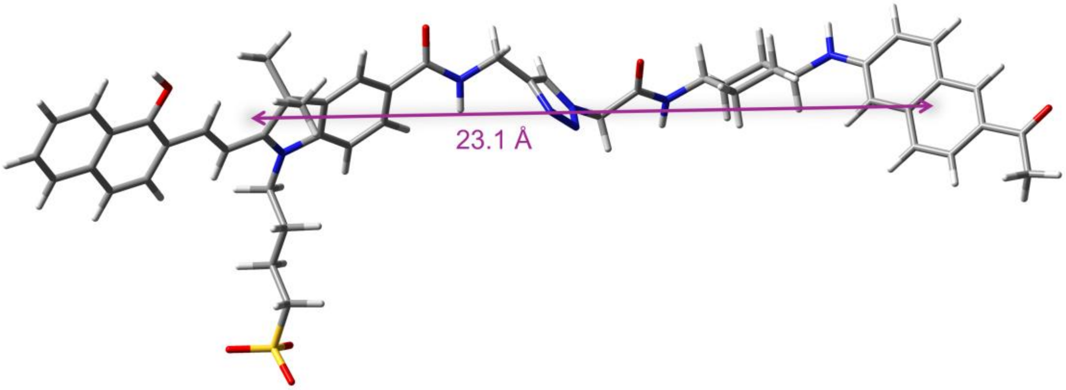

Figure S15. Optimized ground state geometry of the **SNP$_C$/ACD** dyad, calculated at the SMD(water)/M062X/Def2TZVPP level of theory. The purple arrow connects the centers of mass of **ACD** (FRET donor) and **SNP$_C$** (FRET acceptor), with the interchromophore distance indicated.

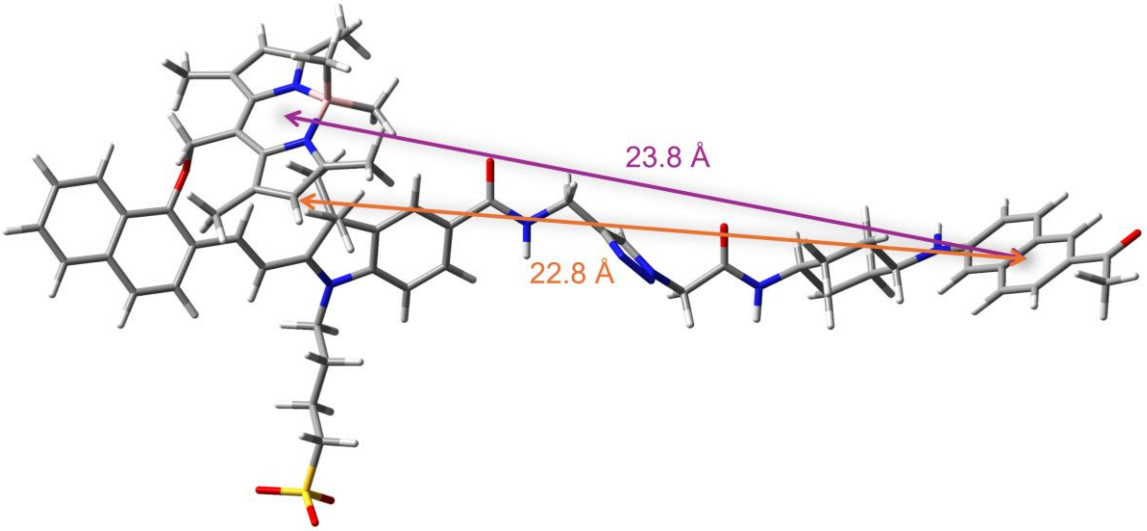

Figure S16. Optimized ground-state geometry of the **BDP/SNP$_C$/ACD** triad, calculated at the SMD(water)/M062X/Def2TZVPP level of theory. The purple arrow connects the centers of mass of **ACD** (FRET donor) and **BDP/SNP** (FRET acceptor), with the interchromophore distance indicated. The orange arrow connects the centers of mass of **ACD** (FRET donor) and **BDP** (FRET acceptor).

Table S1. Additional theoretical insights in FRET for the compounds under study.

Compound	r (Å)[a]	J / 10^{15} (nm^4 M^{-1} cm^{-1})[b]	R_0 (Å)[c]	E_{FRET}[d]
**SNP_C/ACD**	23.1	1.4	45 ± 2	0.9808
**BDP/SNP_C/ACD**	22.8	2.1	47 ± 2	0.9878

[a] FRET-donor-acceptor distance calculated from the optimized ground-state geometry at the SMD(water)/M062X/Def2TZVPP level of theory. [b] Overlap integrals obtained from the experimental absorption and emission spectra of the respective building blocks. Typical error: 10%. [c] Förster radius (R_0) calculated according to standard FRET formalism, using experimental data from Table 1 in manuscript and assuming the orientation factor (κ^2) to be 2/3. Errors in R_0 values were determined by propagating typical deviations of ca. 10% in the fluorescence quantum yield, integral overlap and κ^2, and of ca. 5% for the refractive index. [d] FRET efficiency calculated as $1/[1 + (r/R_0)^6]$.

5b. TDDFT calculations

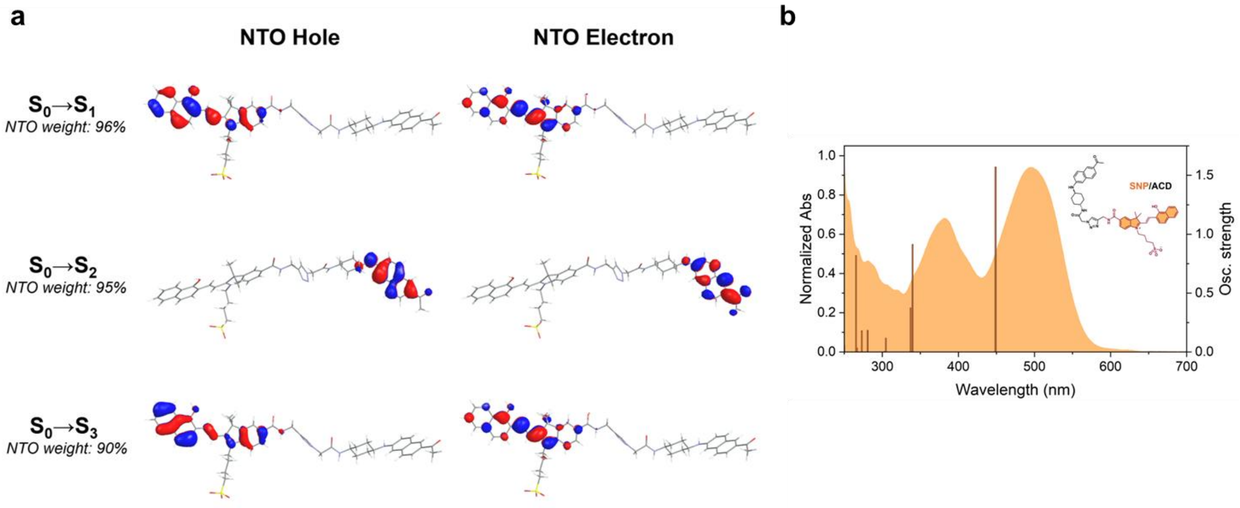

Figure S17. TDDFT calculations for 2for1 dyad. (a) Natural transition orbitals (NTOs) corresponding to the relevant electronic transitions in the UV-Vis region for **SNP$_C$/ACD** (level of theory: SMD(water)/M062X/Def2TZVPP). The percentage contributions of the NTOs to each transitions are indicated. Isosurface value: 0.03 e/bohr^3. (b) Experimental (fill area) and calculated (vertical lines) absorption spectra for **SNP$_C$/ACD**. Level of theory: SMD(water)/M062X/Def2TZVPP).

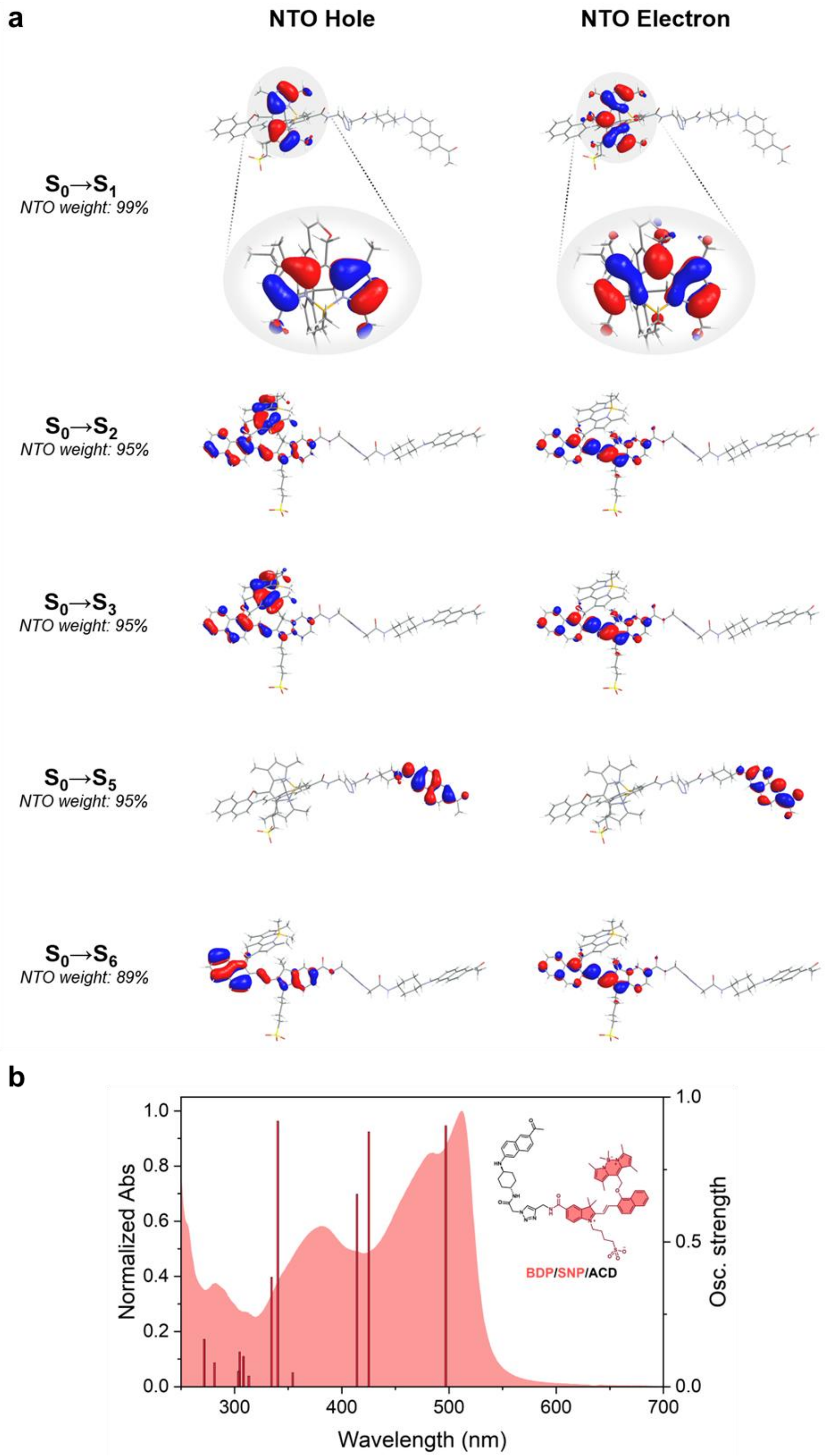

Figure S18. TDDFT calculations for the 3for1 triad. (a) Natural transition orbitals (NTOs) corresponding to the relevant electronic transitions in the UV-Vis region for **BDP/SNP$_C$/ACD** (level of theory: SMD(water)/M062X/Def2TZVPP). The percentage contributions of the NTOs to each transitions are indicated. Isosurface value : 0.03 e/bohr^3. (b) Experimental (fill area) and calculated (vertical lines) absorption spectra for **BDP/SNP$_C$/ACD** obtained at SMD(water)/M062X/Def2TZVPP) level of theory.

Table S2. Calculated electronic and photophysical data for compounds under study.

Compound	Electronic transition	f [a]	Assignation[b]	NTO composition (contribution[c])	NTO weight	E_{theo} (eV)[d] [λ (nm)]	E_{exp} (eV)[e] [λ (nm) – model compound]
**SNP$_C$/ACD**	$S_0 \rightarrow S_1$	1.5714	**SNP**	H-1→LUMO (93)	99	2.76 [449.09]	2.49 [497 – **SNP**]
	$S_0 \rightarrow S_2$	0.9131	**ACD**	HOMO→L+1 (95)	95	3.65 [340.06]	3.42 [363 – **ACD**]
	$S_0 \rightarrow S_3$	0.3732	**SNP**	H-3→LUMO (87)	90	3.68 [337.28]	3.18 [390 – **SNP**]
**BDP/SNP$_C$/ACD**	$S_0 \rightarrow S_1$	0.9012	**BDP**	HOMO→L+1 (96)	99	2.49 [497.35]	2.41 [514 – **BDP**]
	$S_0 \rightarrow S_2$	0.8801	**BDP/SNP**	H-2→LUMO (47), HOMO→LUMO (46)	95	2.91 [425.45]	2.56 [484 – **BDP/SNP**]
	$S_0 \rightarrow S_3$	0.6645	**BDP/SNP**	H-2→LUMO (42), HOMO→LUMO (51)	95	2.99 [414.41]	2.56 [484 – **BDP/SNP**]
	$S_0 \rightarrow S_5$	0.9172	**ACD**	H-1→L+2 (95)	95	3.64 [340.46]	3.42 [363 – **ACD**]
	$S_0 \rightarrow S_6$	0.3770	**SNP**	H-5→LUMO (85)	89	3.70 [334.75]	3.18 [390 – **SNP**]

[a] Oscillator strength. [b] Based on NTOs visual inspection (see below). [c] Percentage contribution approximated by $2c_i^2 \times 100\%$. [c] Absorption energies calculated at the SMD(water)/M062X/Def2TZVPP level of theory. [d] Experimental absorption energies in air-equilibrated aqueous solution of the building blocks composing the compounds, as indicated (please note that the identification of the absorption bands corresponding to the components integrated onto the dyads/triads can be hampered in some cases by spectral overlap; therefore, we chose to compare the theoretical obtained values in the dyad or triad with the experimental ones from the model compounds, which in some cases, as indicated by the interrogation mark, is still not straightforward).

6. Lifetime measurements

6a. TCSPC for the determination of monomer lifetime

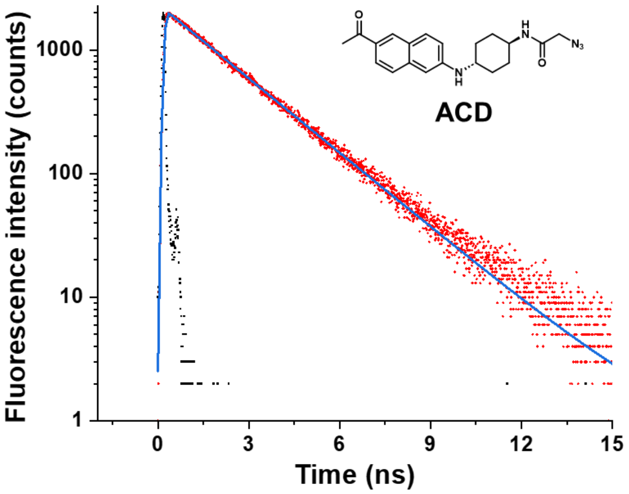

Figure S19. Fluorescence decay traces (red dots) and fitting (blue solid line) at 560 nm of **ACD** monomer excited at 395 nm. The black dots represent instrumental response function. The fluorescence decay showed bi-exponential decay with a major component of 2.0 ns and minor component of 4.7 ns (<10%)

6b. fs-UC for FRET characterization

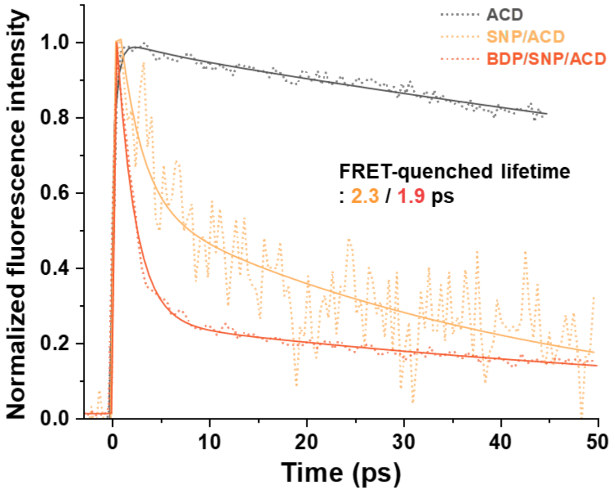

Figure S20. Comparison of fluorescence decays (scattered line) and fitting (smooth line) at 560 nm of **ACD** (black), **SNP$_C$/ACD** (orange), **BDP/SNP$_C$/ACD** (red) excited at 395 nm. The FRET-quenched lifetime in dyad and triad was obtained as 2.3 and 1.9 ps, respectively.

7. References

(1) Frisch, M.; Trucks, G.; Schlegel, H.; Scuseria, G.; Robb, M.; Cheeseman, J.; Scalmani, G.; Barone, V.; Petersson, G.; Nakatsuji, H. Gaussian 16 Revision C. 01, 2016. *Gaussian Inc. Wallingford CT* **2016**, *1*, 572.

(2) Zhao, Y.; Truhlar, D. G. The M06 Suite of Density Functionals for Main Group Thermochemistry, Thermochemical Kinetics, Noncovalent Interactions, Excited States, and Transition Elements: Two New Functionals and Systematic Testing of Four M06-Class Functionals and 12 Other Functionals. *Theor. Chem. Acc.* **2008**, *120*, 215-241.

(3) Weigend, F.; Ahlrichs, R. Balanced Basis Sets of Split Valence, Triple Zeta Valence and Quadruple Zeta Valence Quality for H to Rn: Design and Assessment of Accuracy. *Phys. Chem. Chem. Phys.* **2005**, *7*, 3297-3305.

(4) Marenich, A. V.; Cramer, C. J.; Truhlar, D. G. Universal Solvation Model Based on Solute Electron Density and on a Continuum Model of the Solvent Defined by the Bulk Dielectric Constant and Atomic Surface Tensions. *J. Phys. Chem. B* **2009**, *113*, 6378-6396.

(5) Tomasi, J.; Mennucci, B.; Cammi, R. Quantum Mechanical Continuum Solvation Models. *Chem. Rev.* **2005**, *105*, 2999-3094.

(6) Chung, S.-J.; Zheng, S.; Odani, T.; Beverina, L.; Fu, J.; Padilha, L. A.; Biesso, A.; Hales, J. M.; Zhan, X.; Schmidt, K.; et al. Extended Squaraine Dyes with Large Two-Photon Absorption Cross-Sections. *J. Am. Chem. Soc.* **2006**, *128*, 14444-14445.

(7) Rao, A. S.; Singha, S.; Choi, W.; Ahn, K. H. Studies on Acedan-Based Mononuclear Zinc Complexes toward Selective Fluorescent Probes for Pyrophosphate. *Org. Biomol. Chem.* **2012**, *10*, 8410-8417.

(8) Singha, S.; Kim, D.; Roy, B.; Sambasivan, S.; Moon, H.; Rao, A. S.; Kim, J. Y.; Joo, T.; Park, J. W.; Rhee, Y. M. A Structural Remedy toward Bright Dipolar Fluorophores in Aqueous Media. *Chem. Sci.* **2015**, *6*, 4335-4342.

(9) Gao, H.; Liu, G.; Cui, C.; Wang, M.; Gao, J. Preparation and Properties of a Polyurethane Film Based on Novel Photochromic Spirooxazine Chain Extension. *New J. Chem.* **2022**, *46*, 9128-9137.

(10) Da Lama, A.; Sestelo, J. P.; Sarandeses, L. A.; Martínez, M. M. Microwave-Assisted Direct Synthesis of Bodipy Dyes and Derivatives. *Org. Biomol. Chem.* **2022**, *20*, 9132-9137.

(11) Peterson, J. A.; Fischer, L. J.; Gehrmann, E. J.; Shrestha, P.; Yuan, D.; Wijesooriya, C. S.; Smith, E. A.; Winter, A. H. Direct Photorelease of Alcohols from Boron-Alkylated Bodipy Photocages. *J. Org. Chem.* **2020**, *85*, 5712-5717.

(12) Andraos, J. A Streamlined Approach to Solving Simple and Complex Kinetic Systems Analytically. *J. Chem. Educ.* **1999**, *76*, 1578.

8. NMR spectra

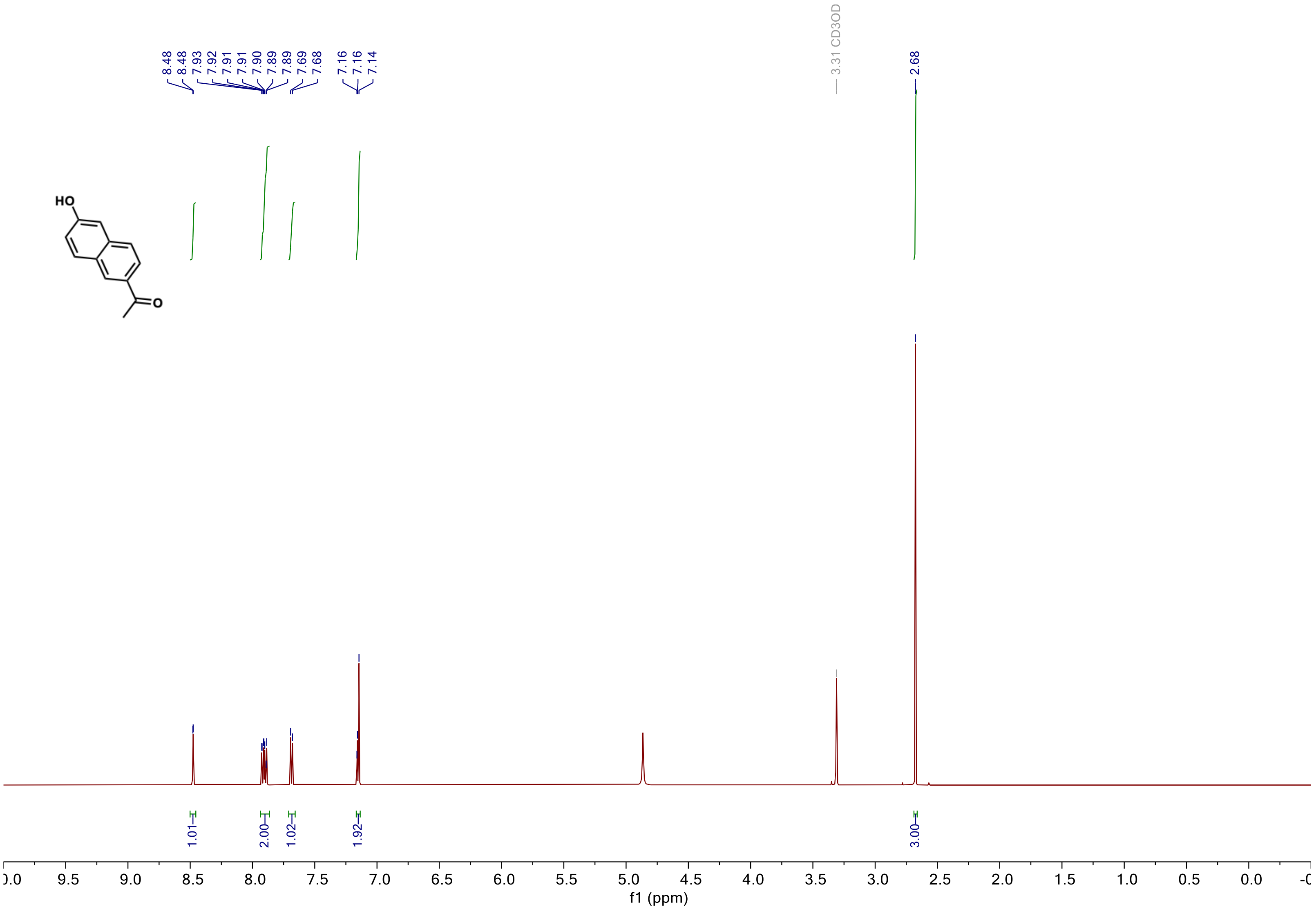

Figure S21 ^{1}H NMR spectrum (600 MHz, CD_3OD) of **A1**.

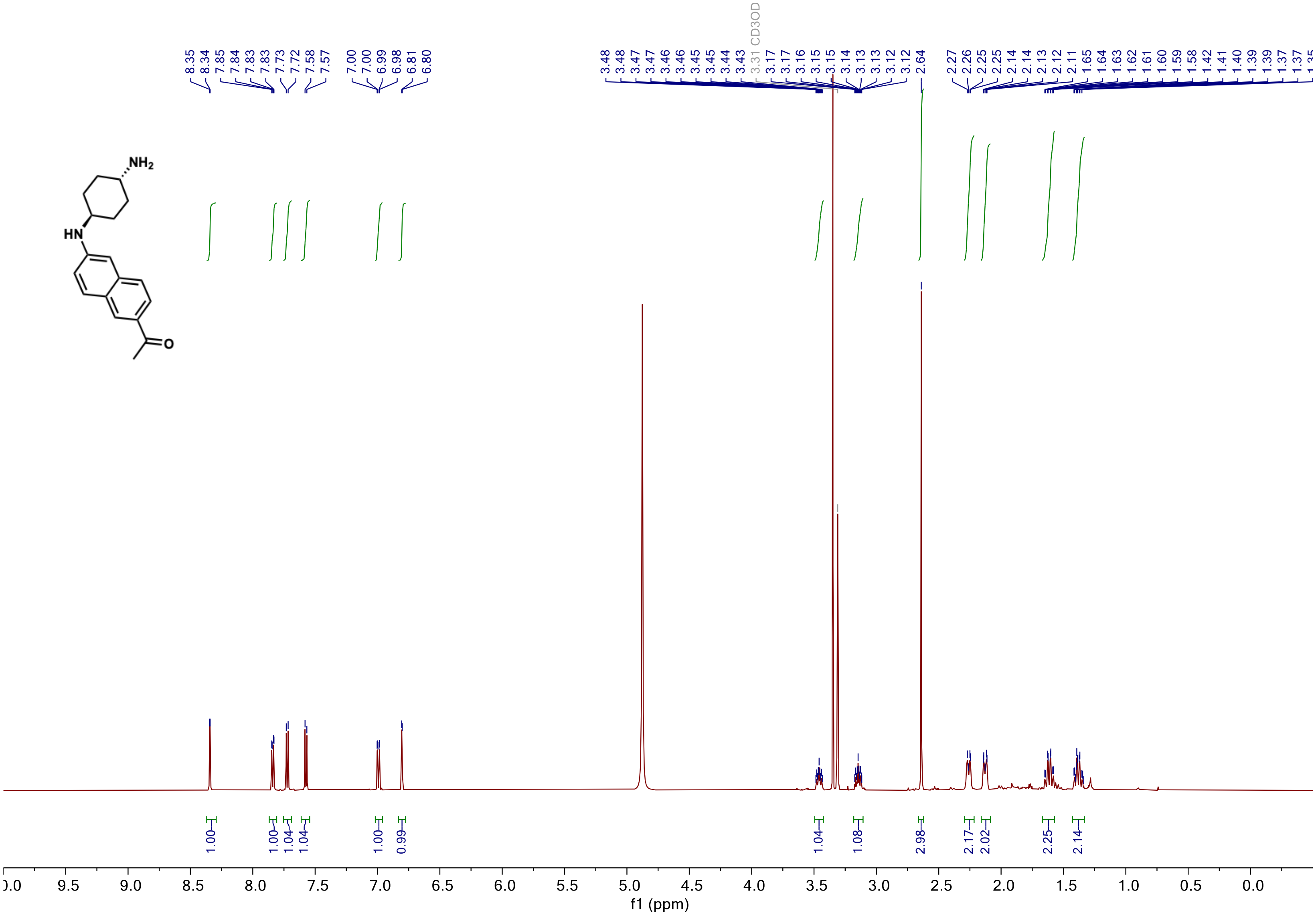

Figure S22 ^{1}H NMR spectrum (600 MHz, CD_3OD) of **A2**

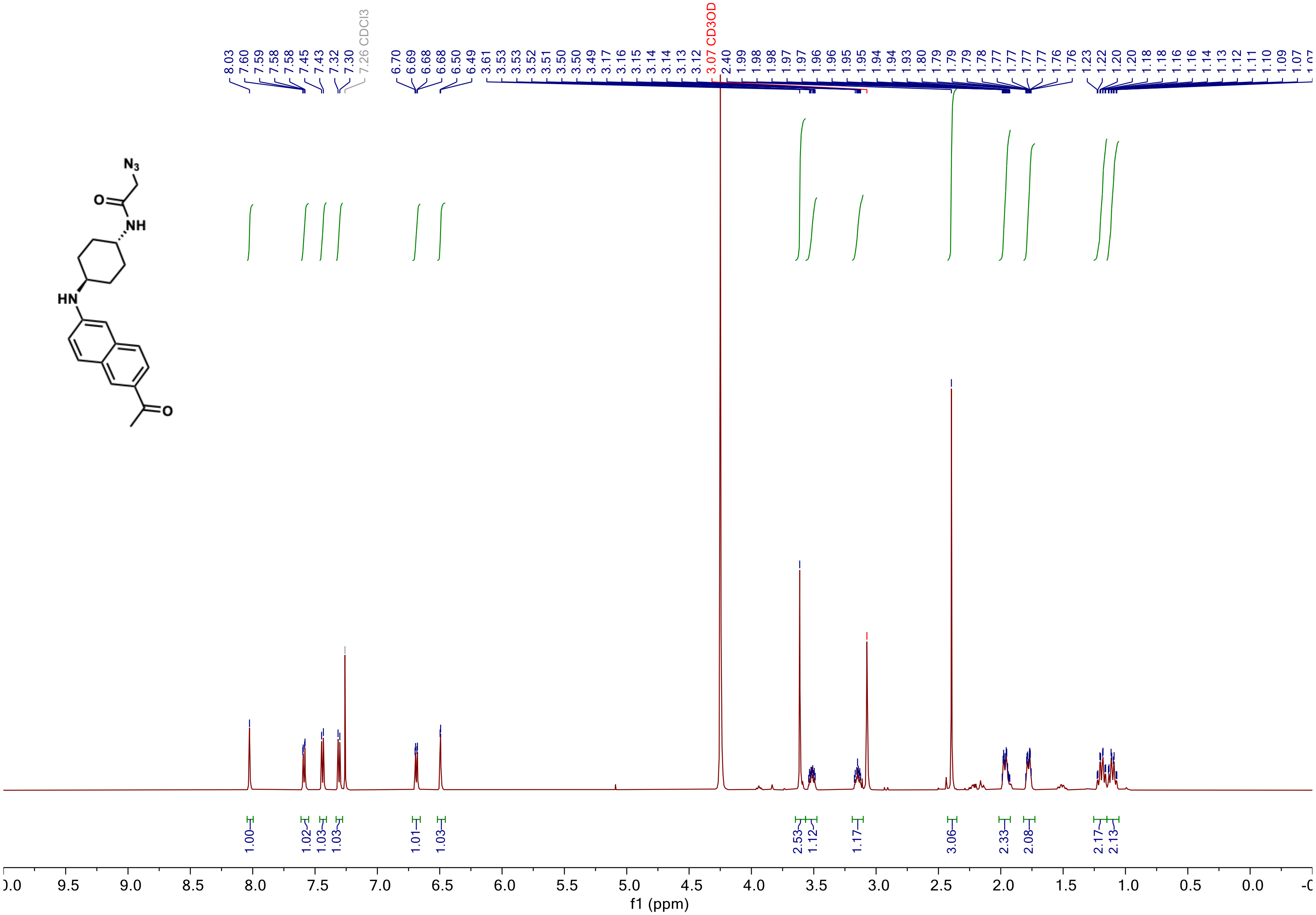

Figure S23 ^{1}H NMR spectrum (600 MHz, $CD_3OD/CDCl_3$) of **ACD**.

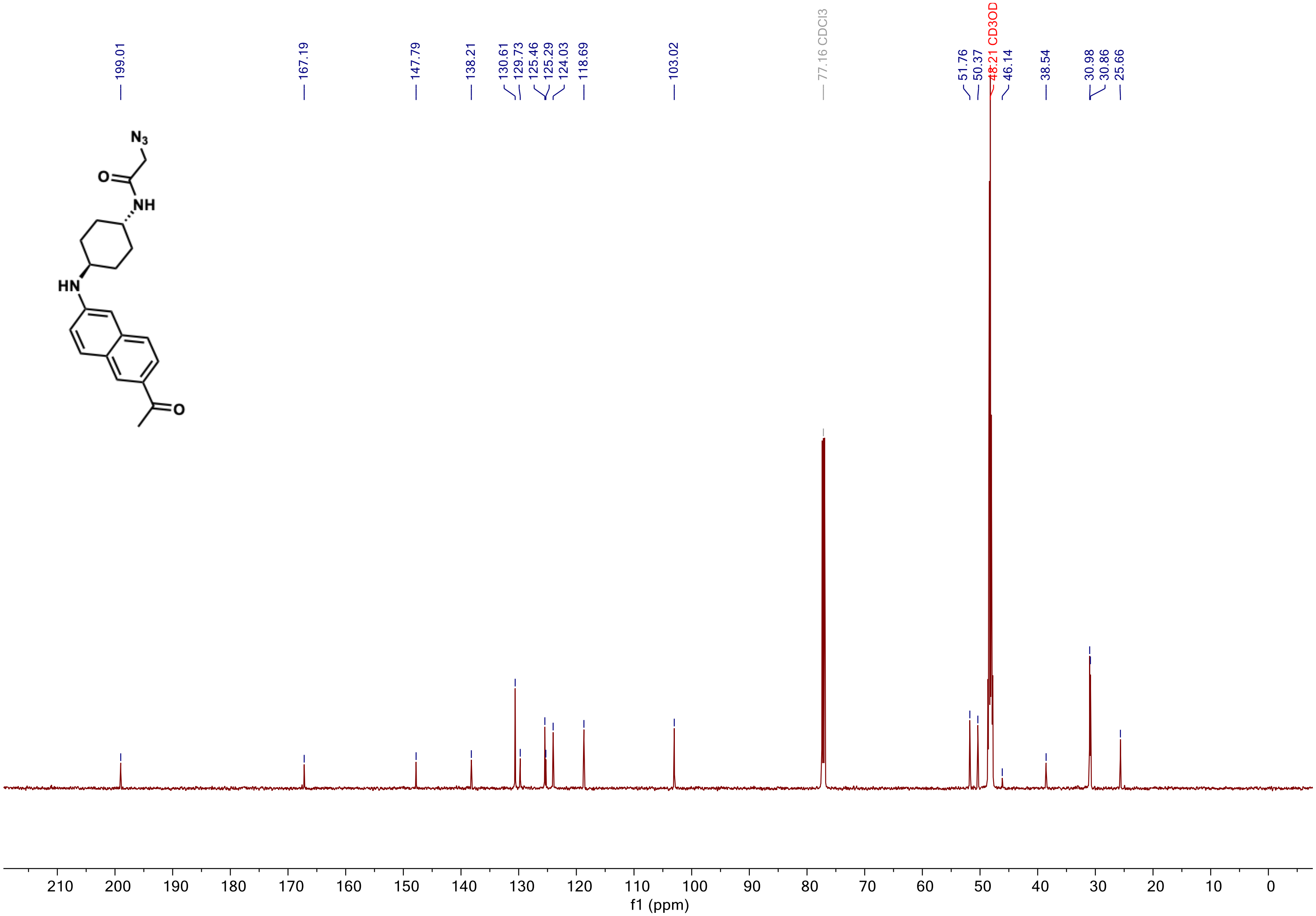

Figure S24 ^{13}C NMR spectrum (151 MHz, $CD_3OD/CDCl_3$) of **ACD**.

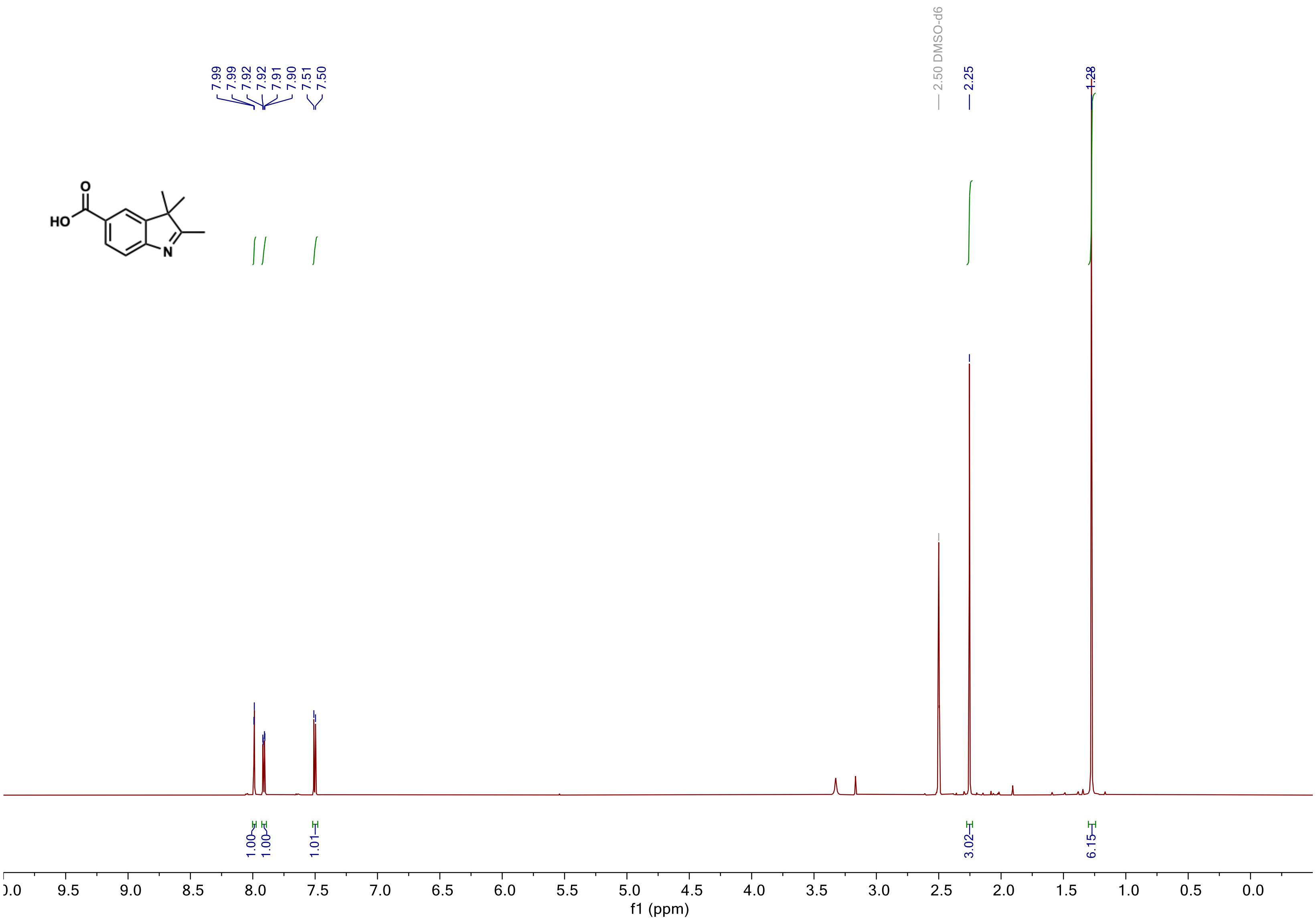

Figure S25 ^{1}H NMR spectrum (600 MHz, DMSO-d_6) of **S1**.

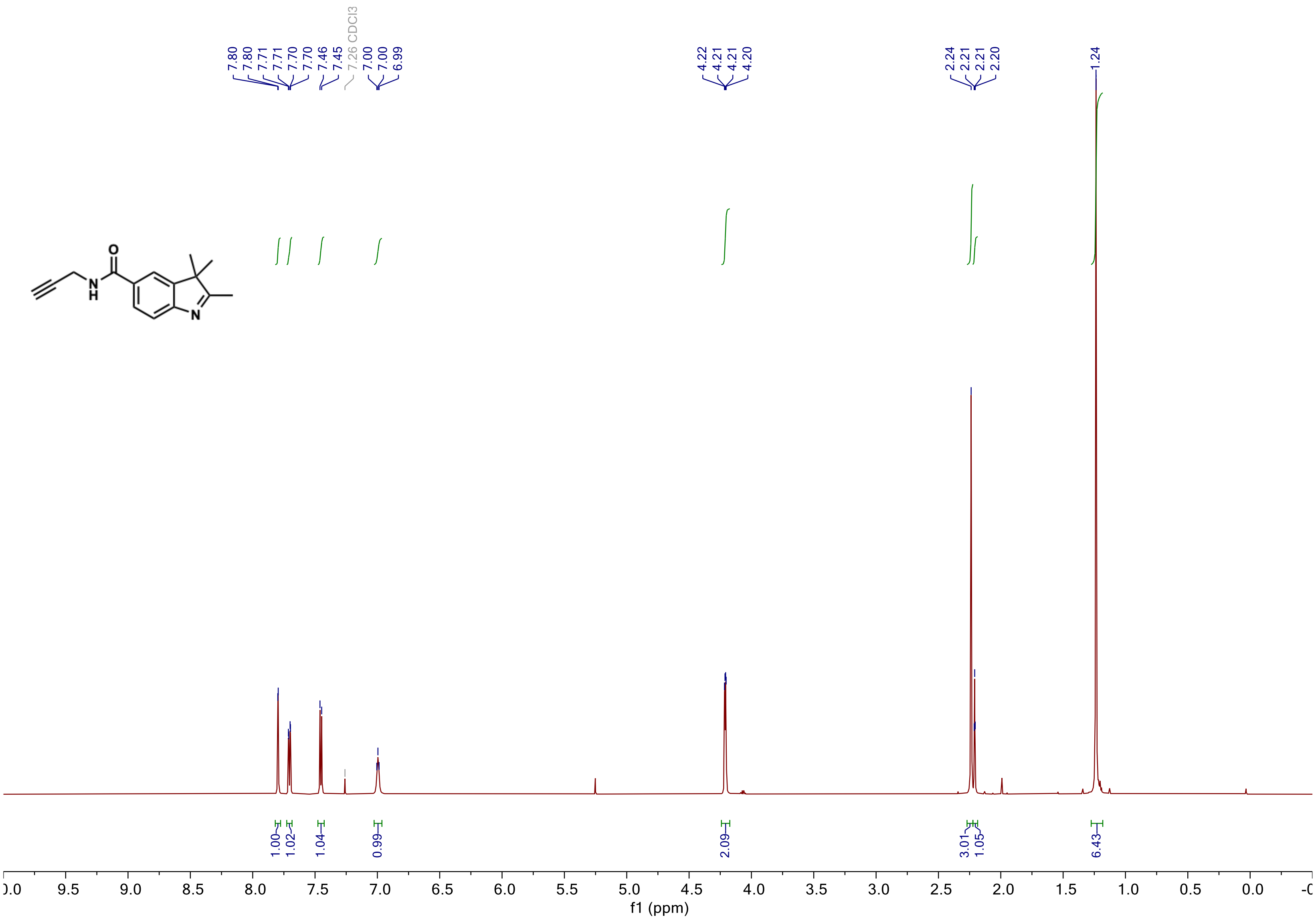

Figure S26 ^{1}H NMR spectrum (600 MHz, $CDCl_3$) of **S2**.

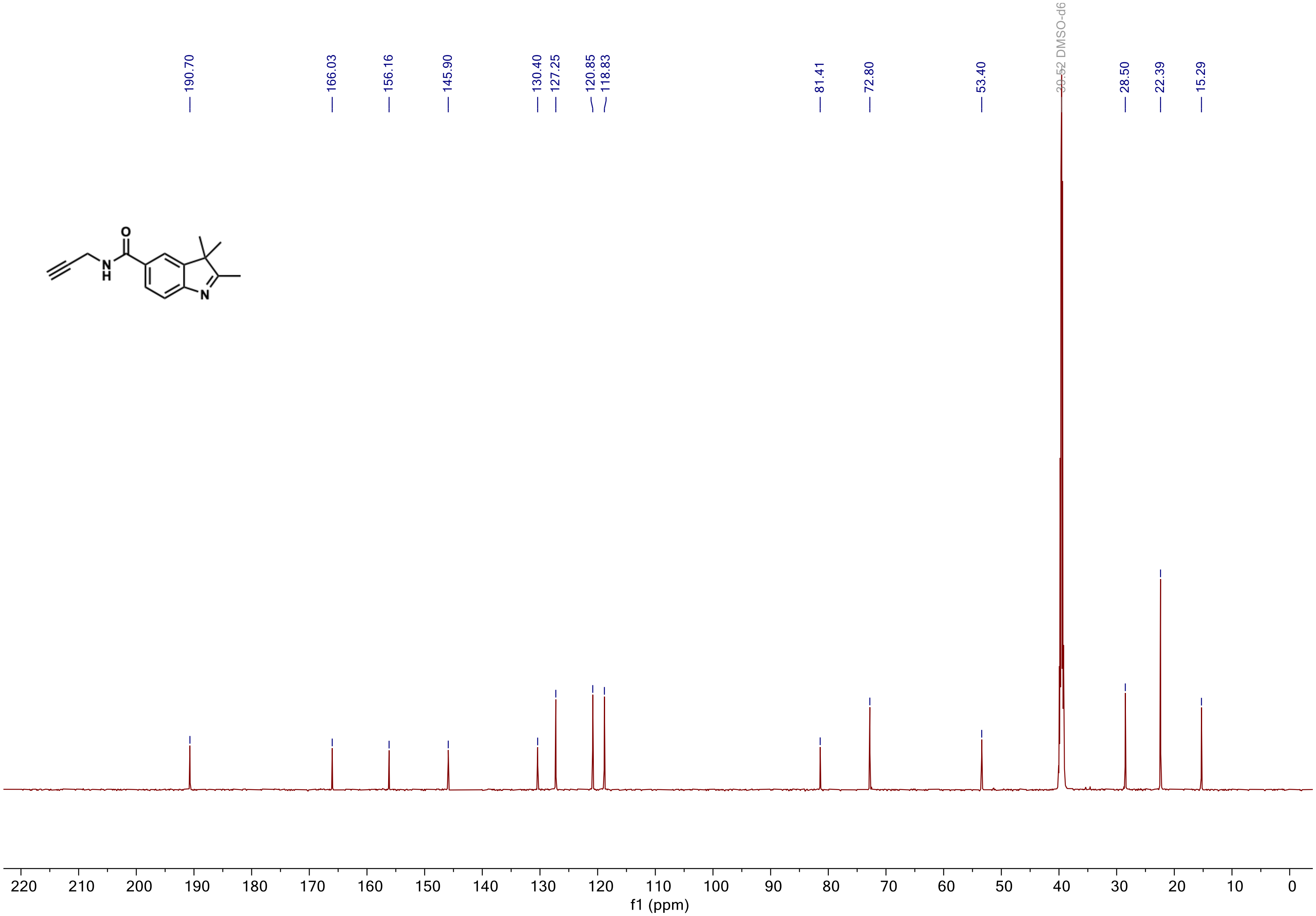

Figure S27 ^{13}C NMR spectrum (151 MHz, DMSO-d_6) of **S2**.

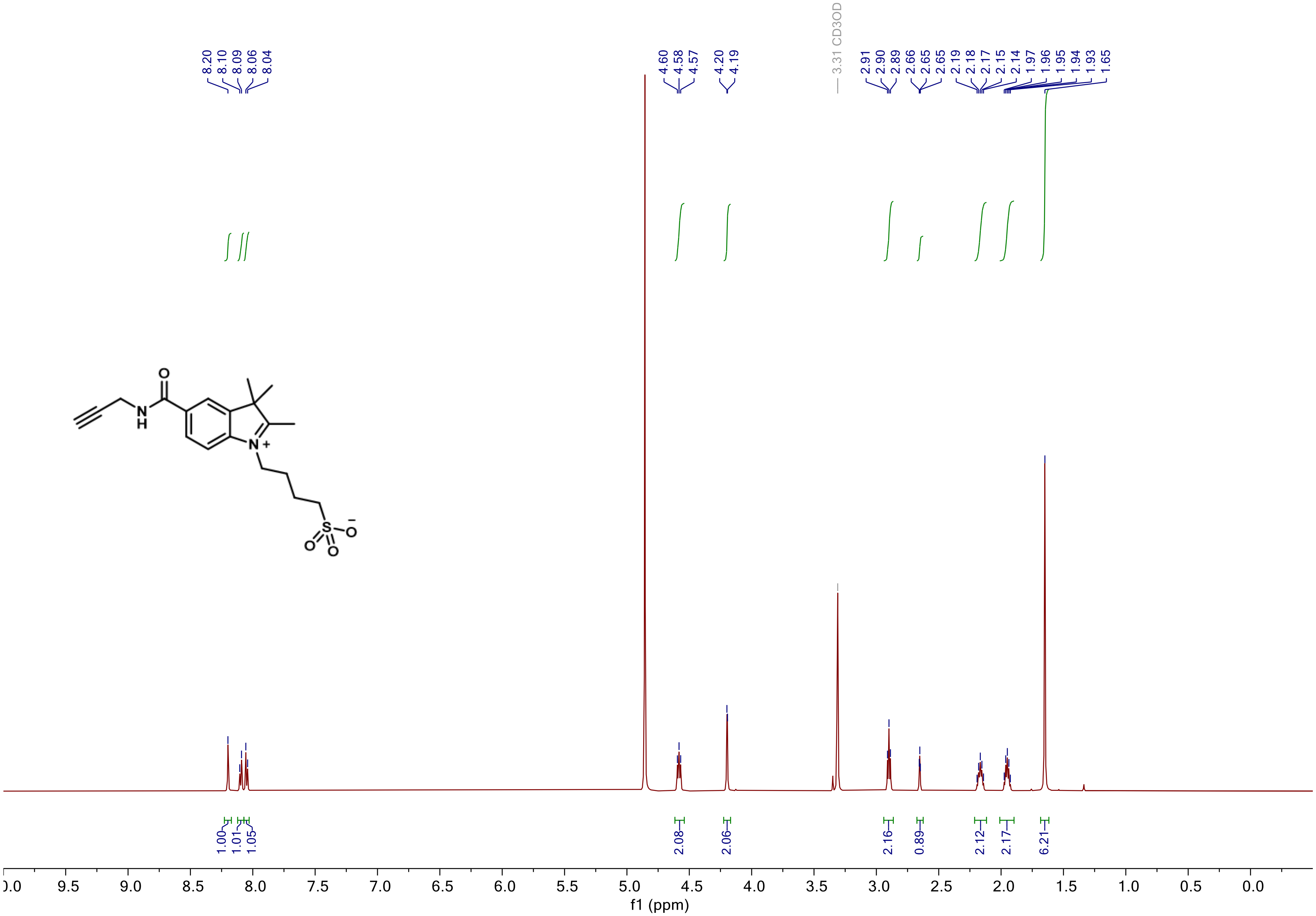

Figure S28 ^{1}H NMR spectrum (600 MHz, CD_3OD) of **S3**.

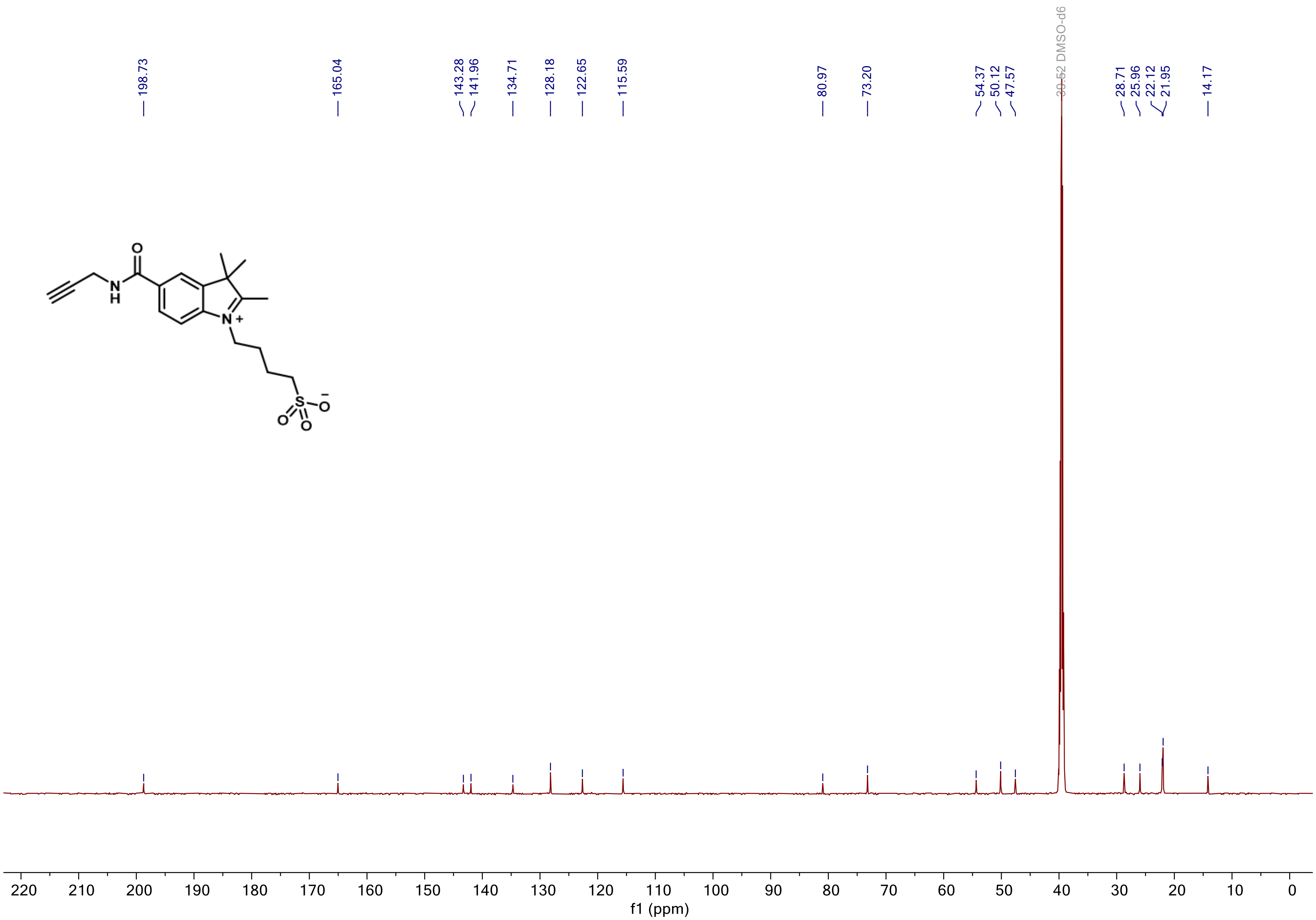

Figure S29 ^{13}C NMR spectrum (151 MHz, CD_3OD) of **S3**.

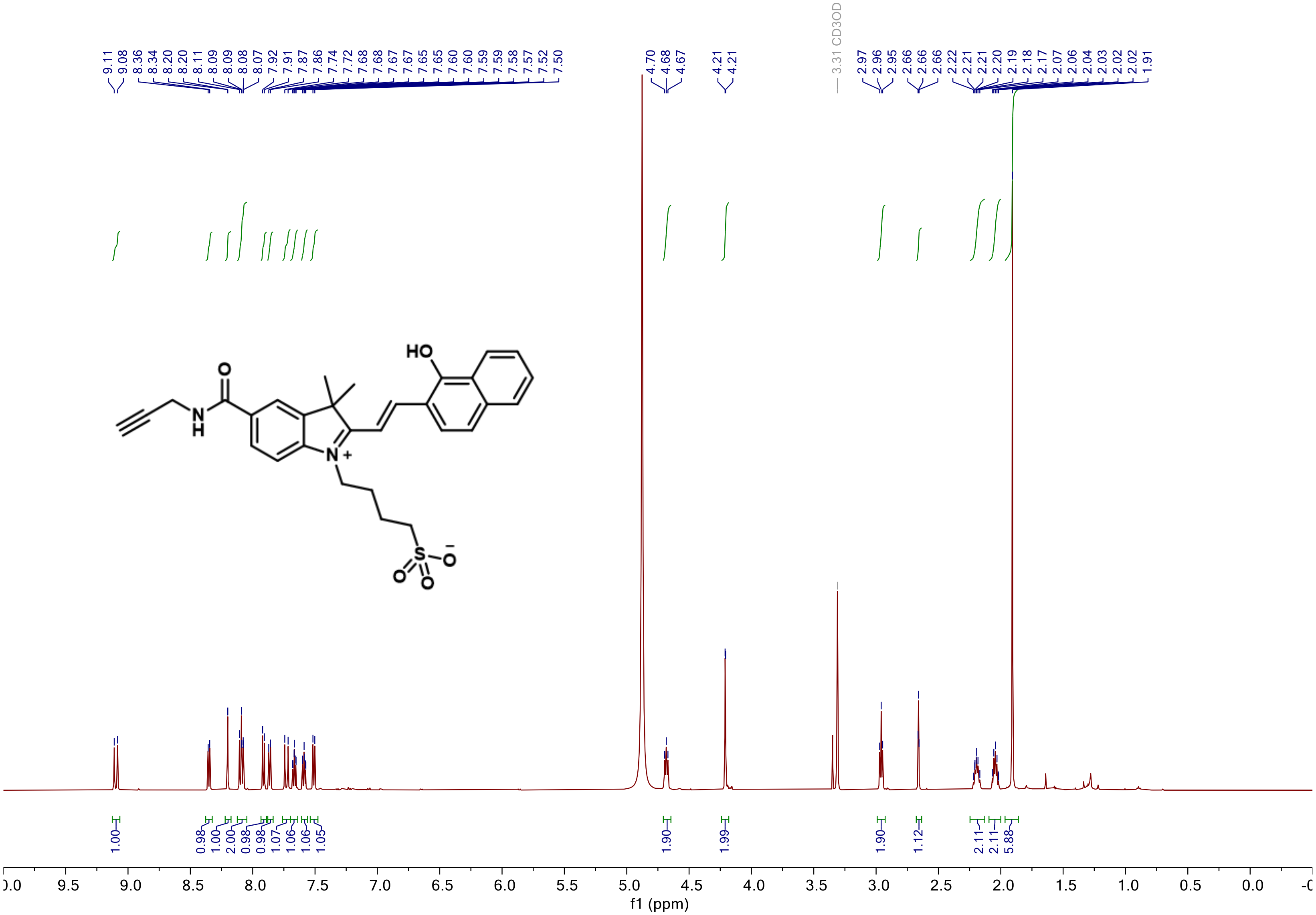

Figure S30 ^{1}H NMR spectrum (600 MHz, CD_3OD) of **SNP**.

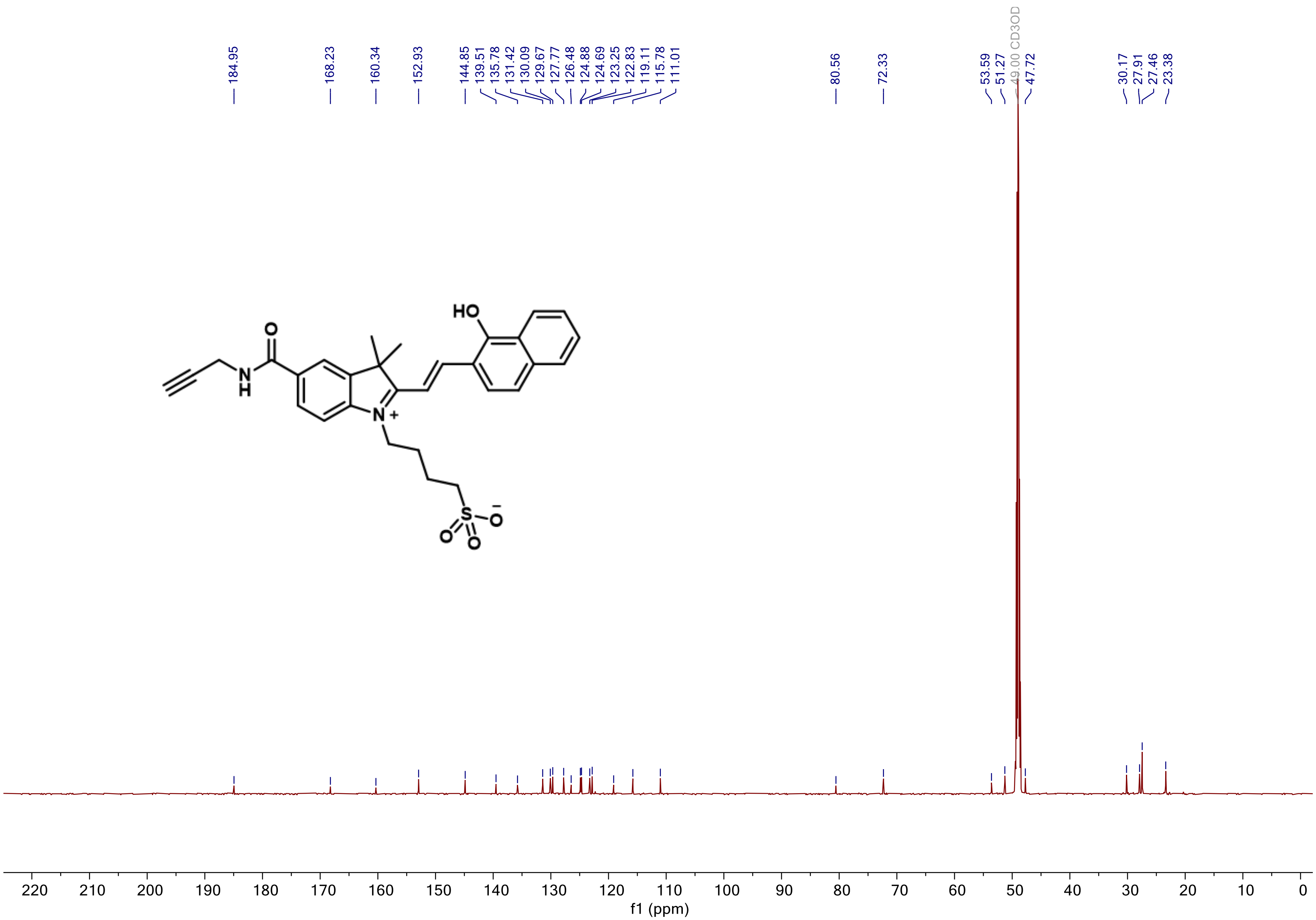

Figure S31 ^{13}C NMR spectrum (151 MHz, CD_3OD) of **SNP**.

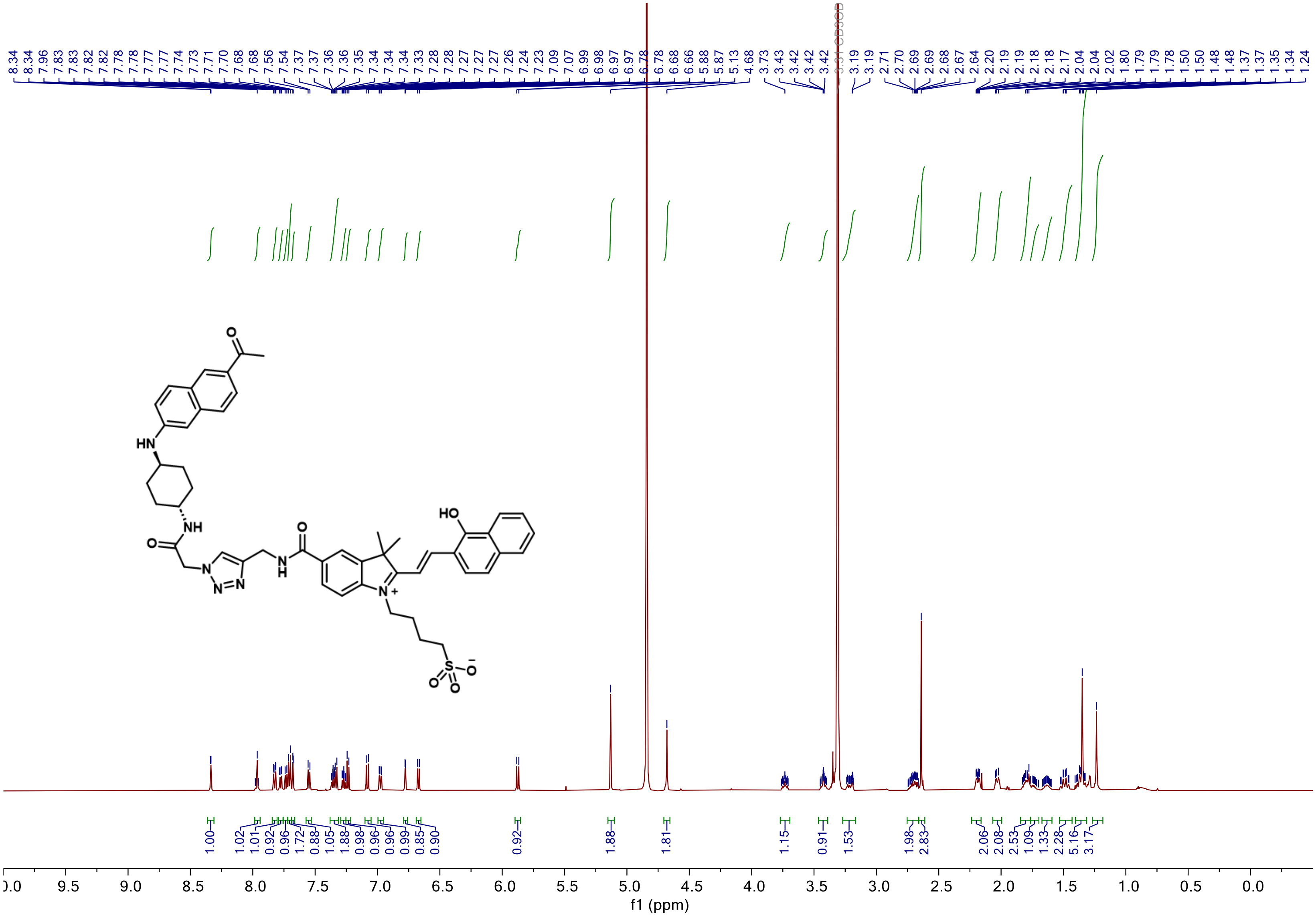

Figure S32 ^{1}H NMR spectrum (600 MHz, CD_3OD) of **SNP/ACD**.

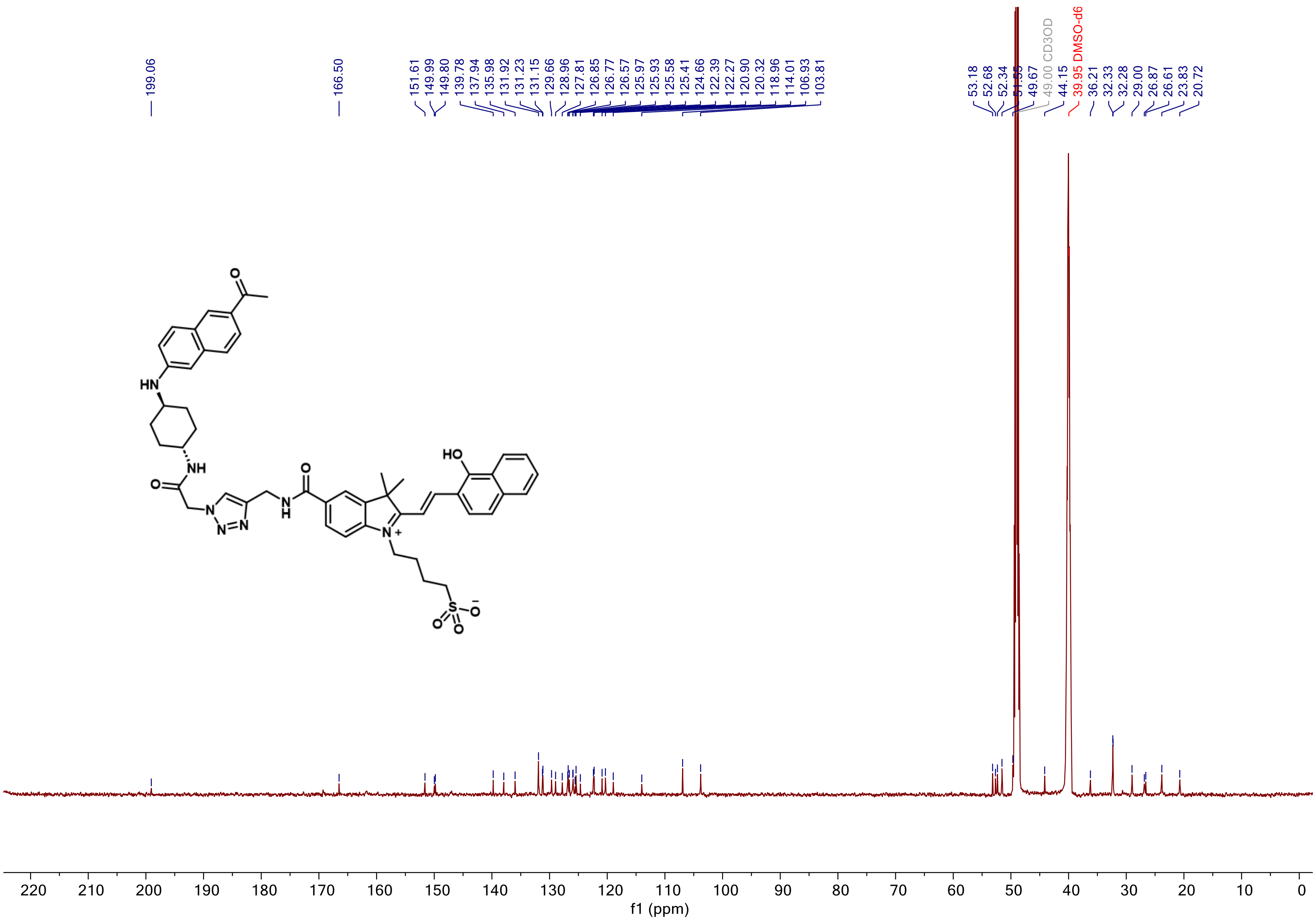

Figure S33 ^{13}C NMR spectrum (151 MHz, CD_3OD/DMSO-d_6) of **SNP/ACD**.

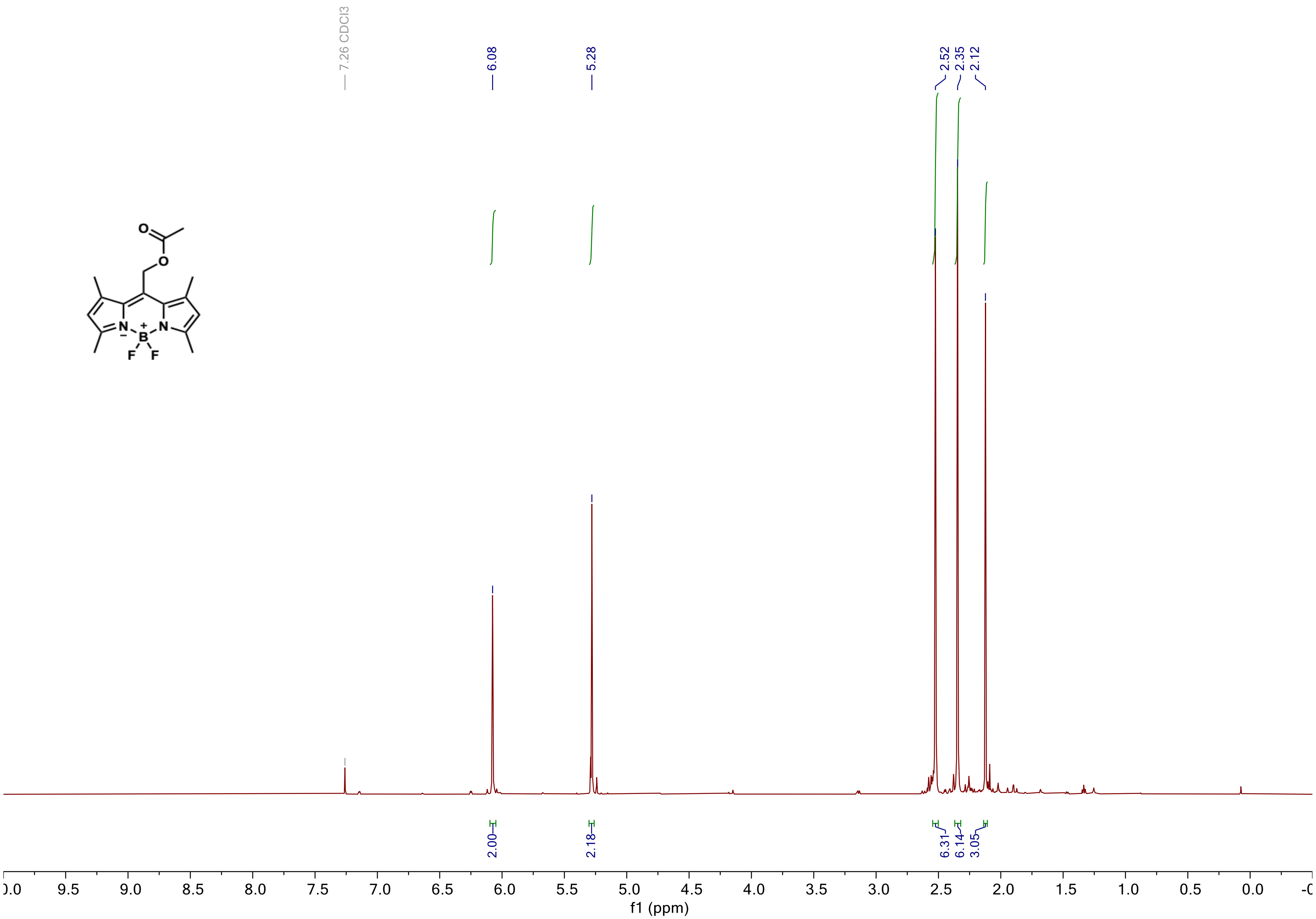

Figure S34 ^{1}H NMR spectrum (600 MHz, $CDCl_3$) of **B1**.

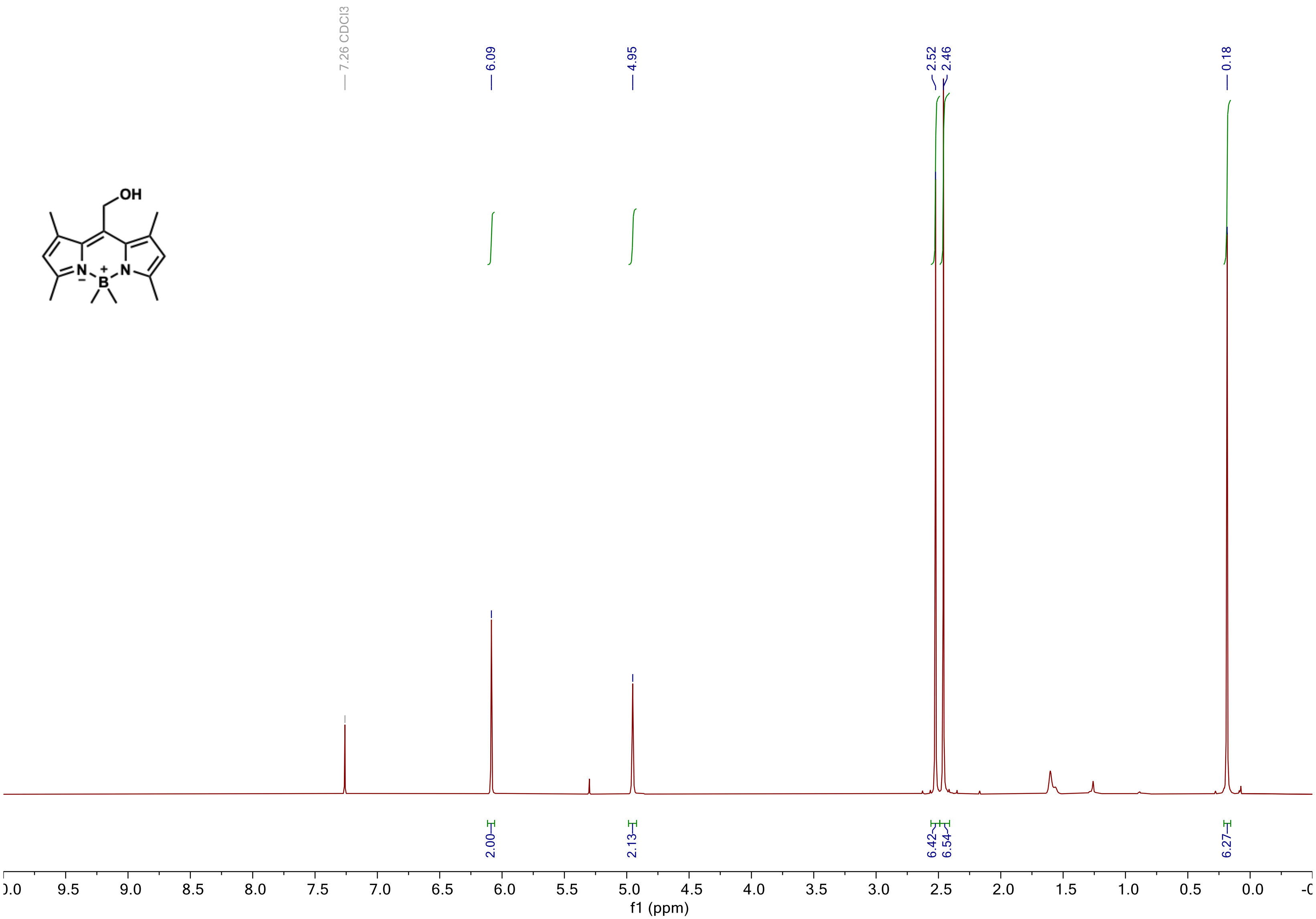

Figure S35 ^{1}H NMR spectrum (600 MHz, $CDCl_3$) of **B2**.

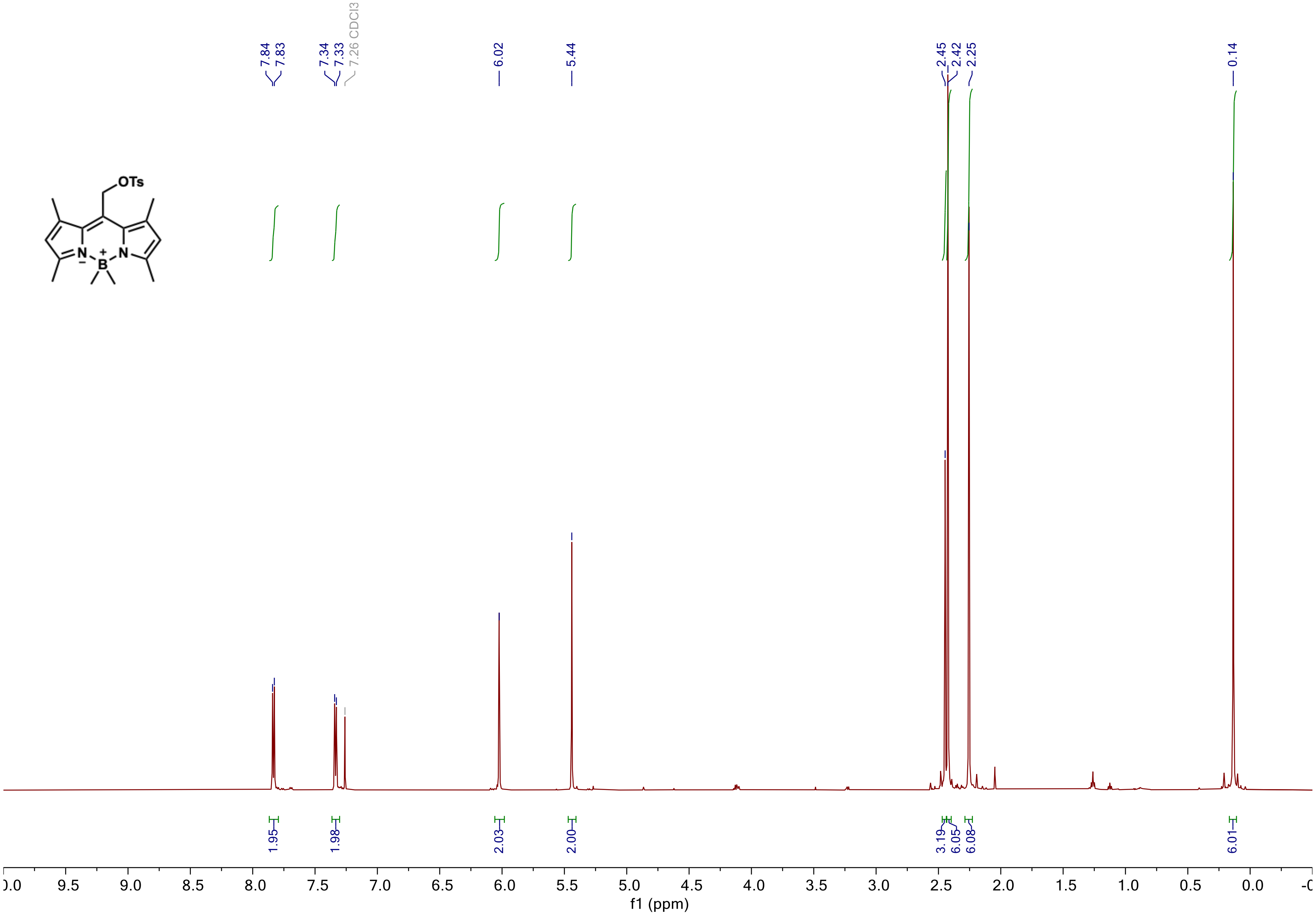

Figure S36 ^{1}H NMR spectrum (600 MHz, $CDCl_3$) of **B3**.

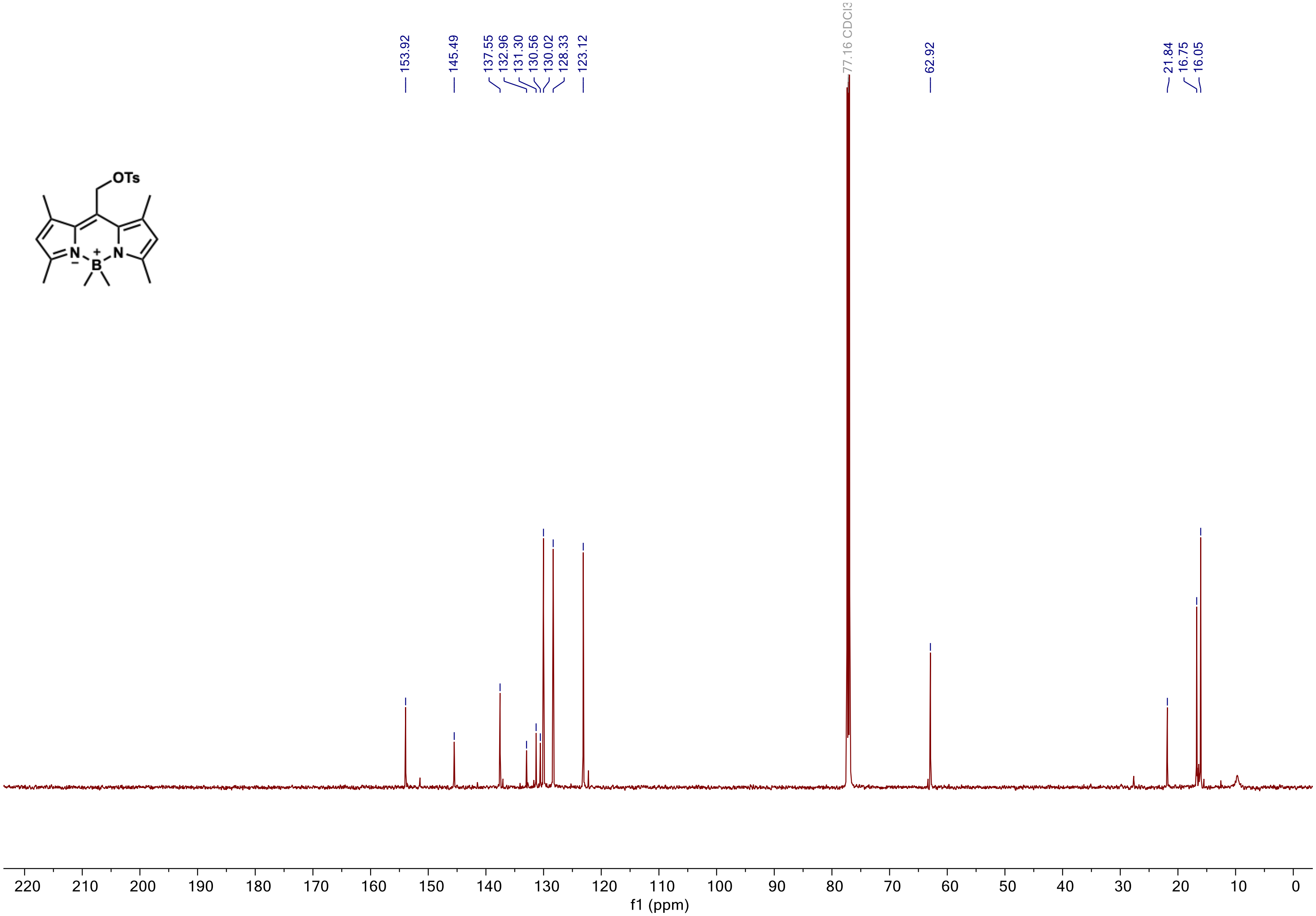

Figure S37 ^{13}C NMR spectrum (151 MHz, $CDCl_3$) of **B3**.

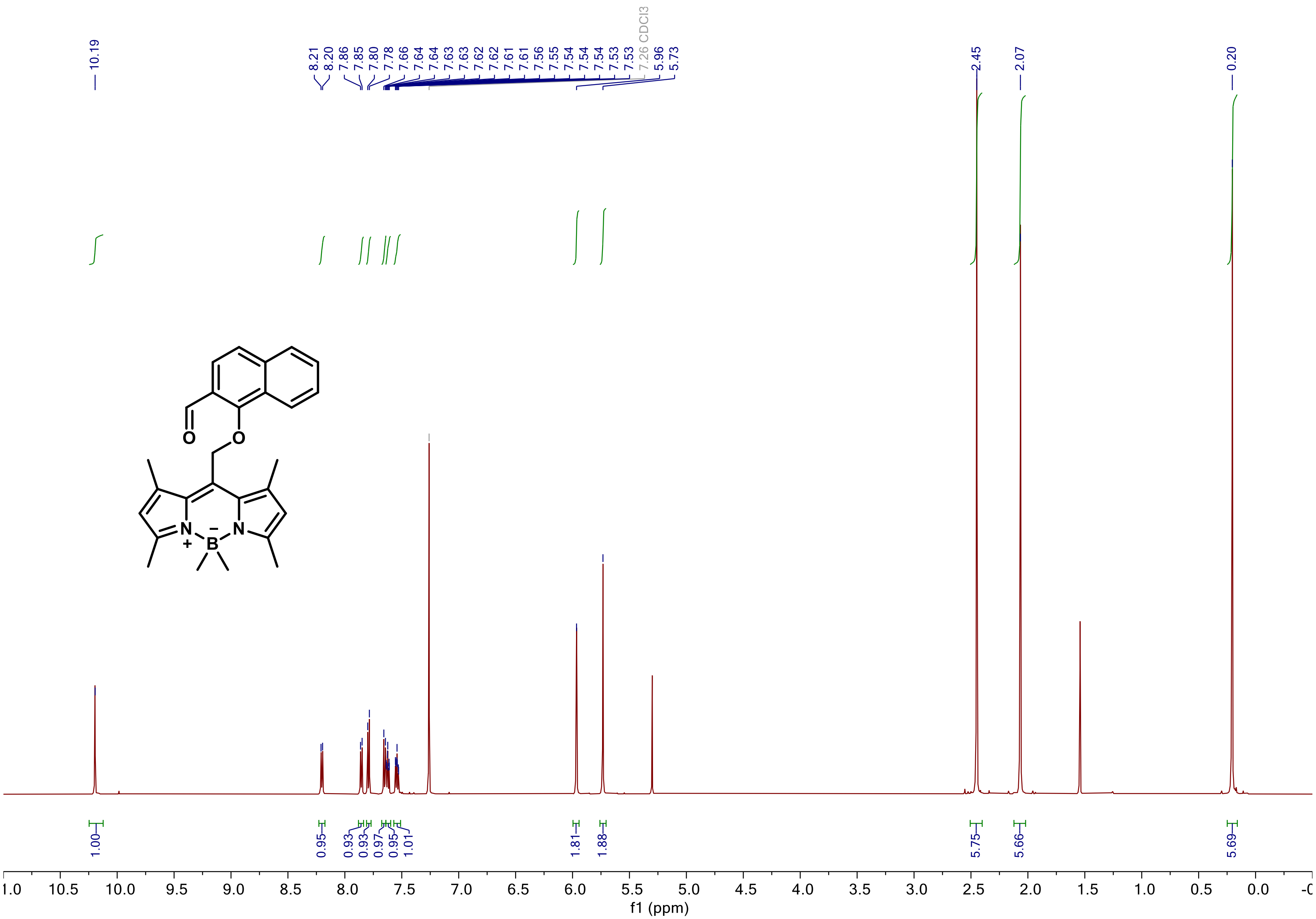

Figure S38 ^{1}H NMR spectrum (600 MHz, $CDCl_3$) of **BDP**.

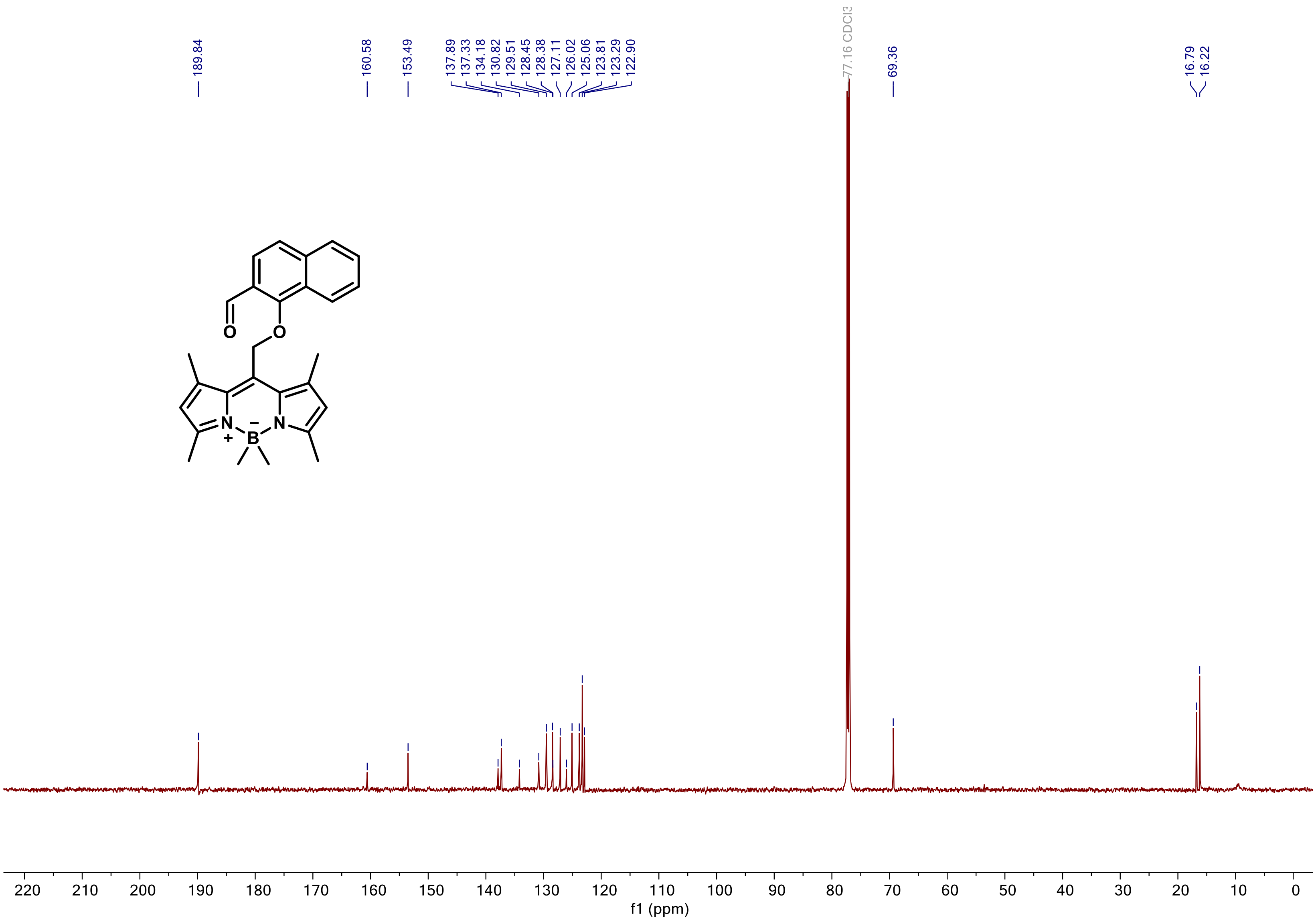

Figure S39 ^{13}C NMR spectrum (151 MHz, $CDCl_3$) of **BDP**.

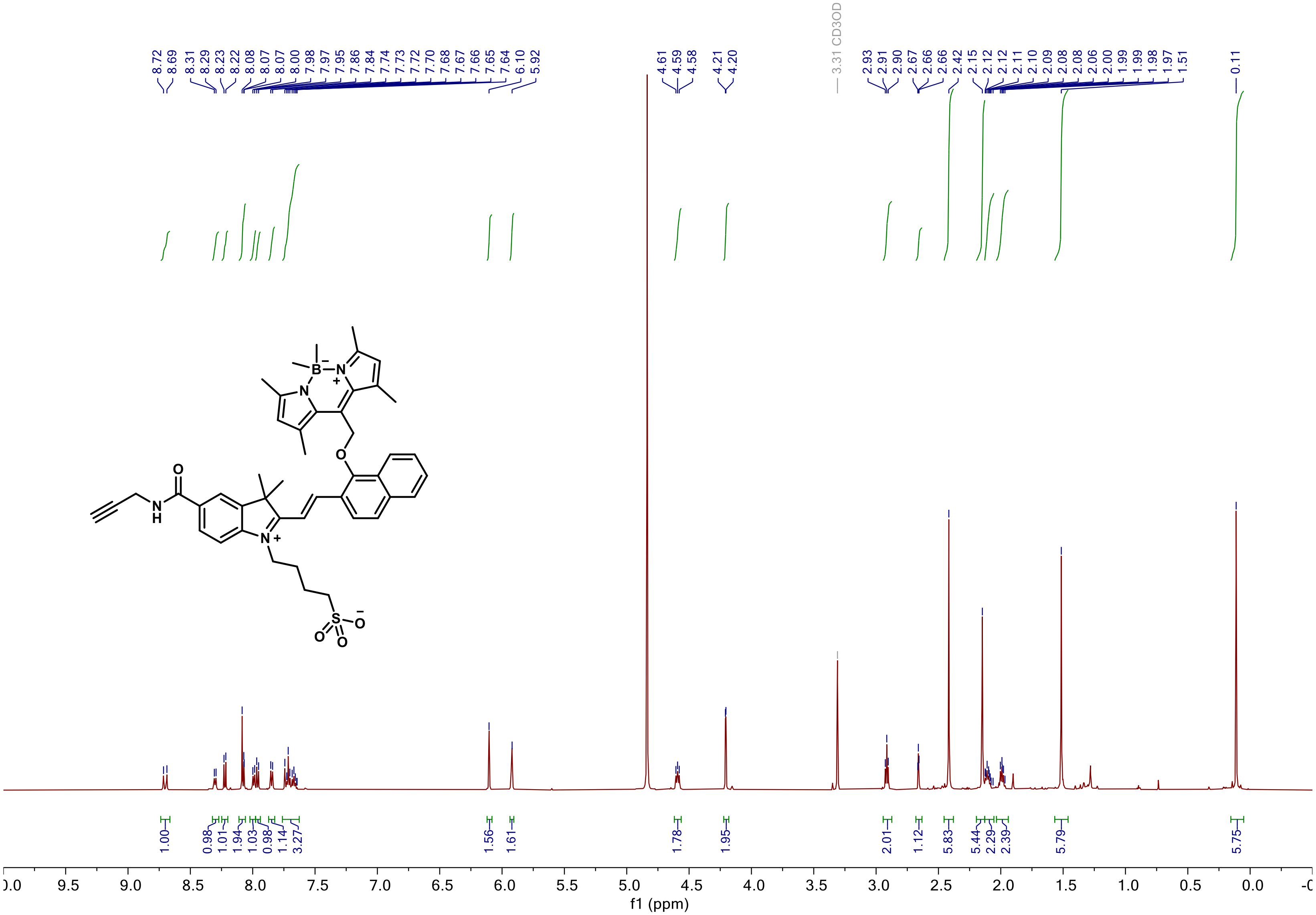

Figure S40 ^{1}H NMR spectrum (600 MHz, CD_3OD) of **BDP/SNP**.

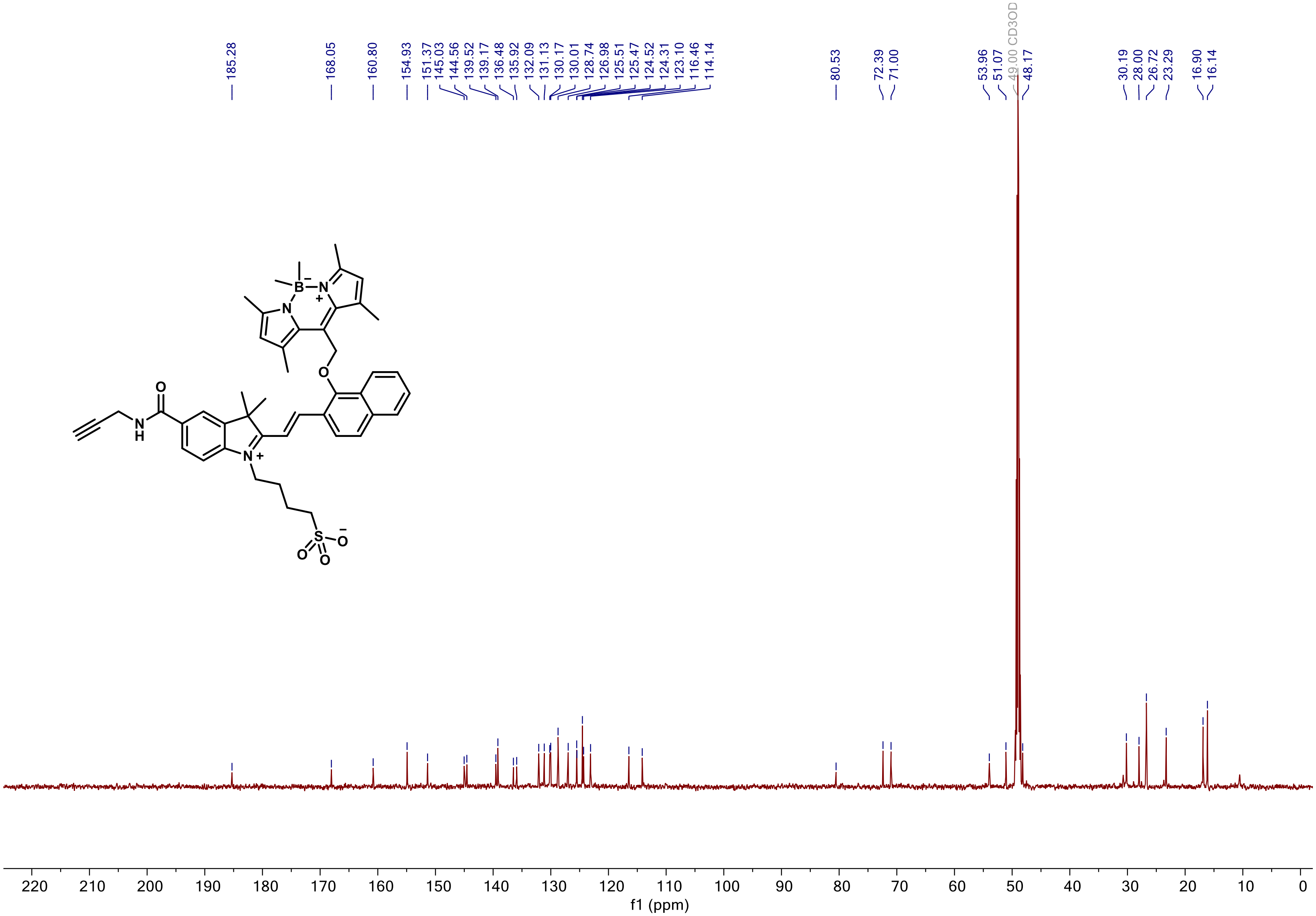

Figure S41 ^{13}C NMR spectrum (151 MHz, CD_3OD) of **BDP/SNP**.

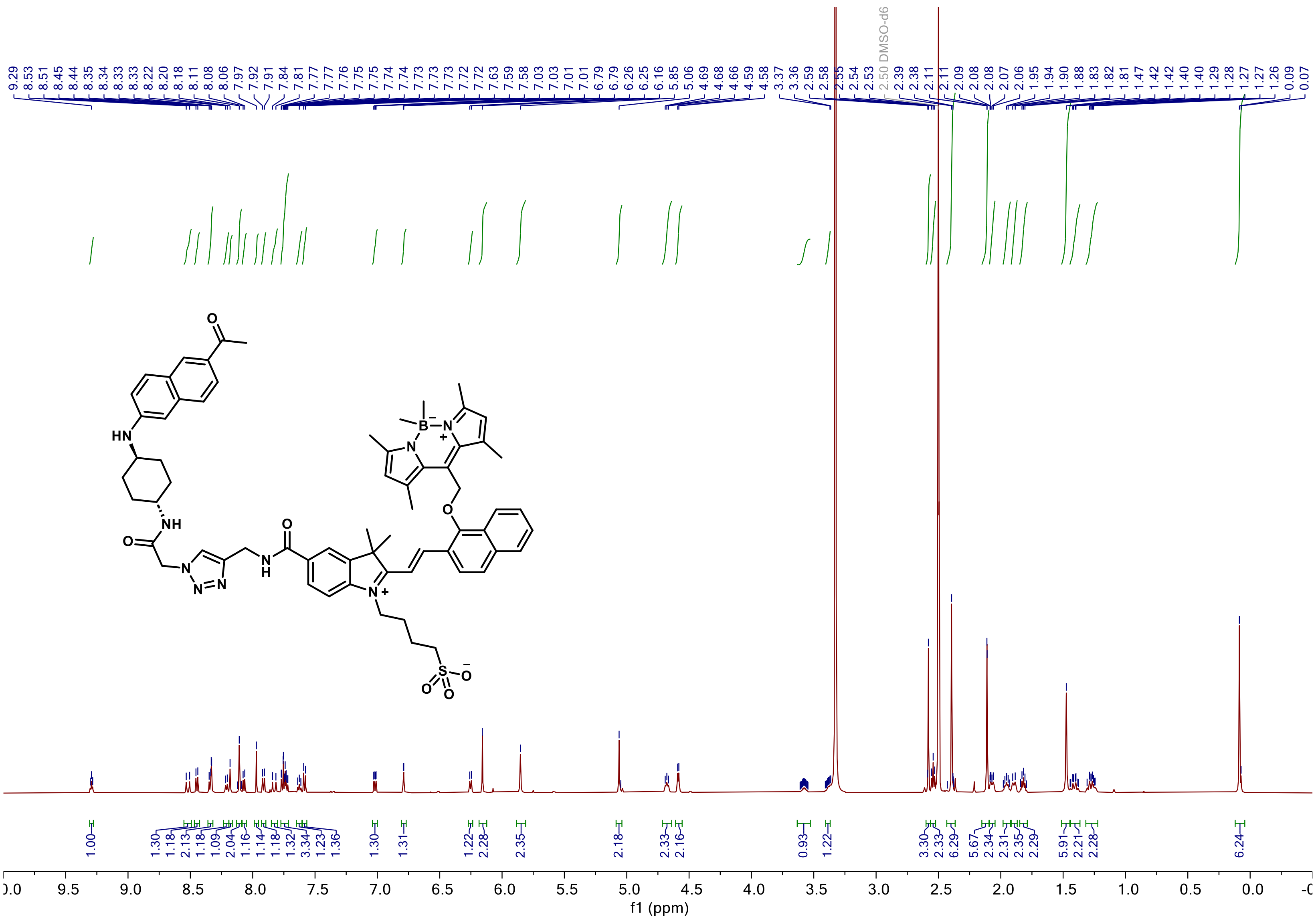

Figure S42 ^{1}H NMR spectrum (600 MHz, DMSO-d_6) of **BDP/SNP/ACD**.

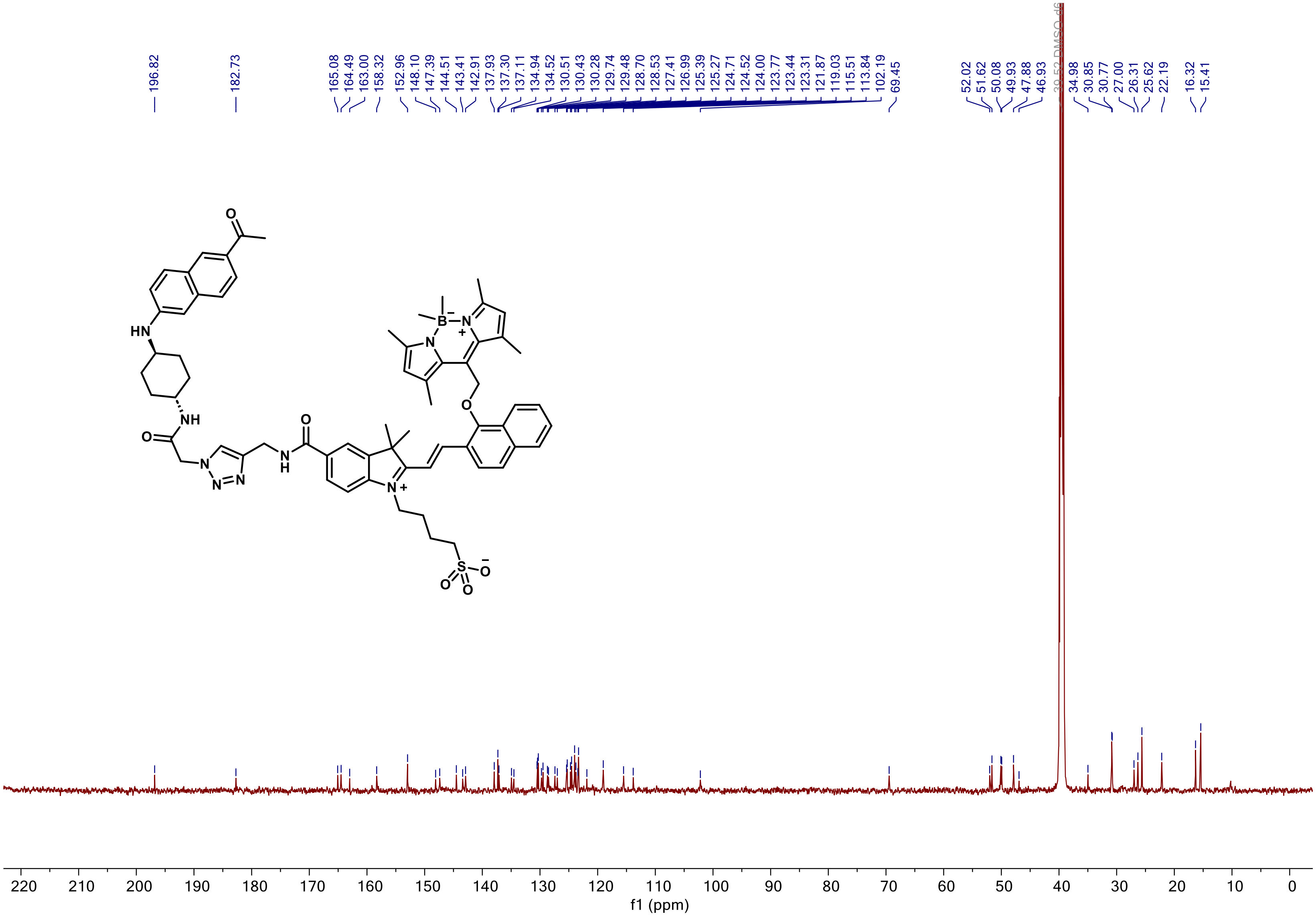

Figure S43 ^{13}C NMR spectrum (151 MHz, DMSO-d_6) of **BDP/SNP/ACD**.

9. Atomic coordinates of optimized structures at the ground state

Table S3. Atomic coordinates for the optimized ground state of **SNP/ACD** at the SMD(water)/M062X/Def2TZVPP level of theory.

**Energy (Hartree)**	-3245.348547
**Imaginary frequencies**	0

**Atom type**		**Atomic coordinates**		
		x	y	z
1	C	-3.17457	1.75501	-0.87139
2	C	-3.25519	0.46775	-1.40384
3	H	-2.37564	-0.01434	-1.80793
4	C	-4.46154	-0.21810	-1.45492
5	H	-4.51426	-1.21377	-1.87080
6	C	-5.56839	0.43578	-0.94859
7	C	-5.51287	1.71125	-0.41229
8	C	-4.31593	2.39048	-0.37755
9	H	-4.24688	3.38990	0.03152
10	N	-6.90291	-0.02414	-0.86983
11	C	-7.68436	0.86207	-0.28083
12	C	-6.88492	2.11070	0.06488
13	C	-7.27770	-1.36624	-1.31898
14	H	-8.30759	-1.34153	-1.66370
15	H	-6.65397	-1.59976	-2.17953
16	C	-7.08348	-2.38865	-0.21018
17	H	-6.04285	-2.36505	0.12166
18	H	-7.70800	-2.11261	0.64280
19	C	-7.44583	-3.78546	-0.69673
20	H	-6.82505	-4.04516	-1.55632
21	H	-8.48595	-3.79818	-1.02814
22	C	-9.05827	0.59669	-0.02777
23	H	-9.42499	-0.38293	-0.29659
24	C	-7.37790	3.34116	-0.71363
25	H	-7.43252	3.12869	-1.78087
26	H	-6.66719	4.15183	-0.55481
27	H	-8.35540	3.66882	-0.36664
28	C	-6.85667	2.37186	1.57857
29	H	-6.13662	3.16611	1.77411
30	H	-6.54672	1.47848	2.12008
31	H	-7.82857	2.69079	1.94829
32	C	-7.24698	-4.81160	0.40546
33	H	-7.87364	-4.59308	1.27097
34	H	-6.20696	-4.84800	0.73179
35	S	-7.66249	-6.46488	-0.09790
36	C	-1.88556	2.51279	-0.81407
37	N	-0.75333	1.80101	-0.82089

38	H	-0.78518	0.79455	-0.77334
39	O	-1.87484	3.74577	-0.75699
40	C	0.54435	2.46231	-0.75802
41	H	0.61947	3.17954	-1.57402
42	H	0.62616	3.01602	0.17853
43	C	1.64592	1.47015	-0.85769
44	O	-6.79098	-6.78797	-1.22923
45	O	-7.40472	-7.31047	1.06847
46	O	-9.07786	-6.44396	-0.47323
47	C	-9.89761	1.50432	0.52576
48	H	-9.51874	2.47924	0.78975
49	C	-11.29735	1.29675	0.79574
50	C	-11.95851	0.06619	0.50971
51	H	-11.38952	-0.74855	0.08450
52	C	-13.27923	-0.10248	0.76176
53	H	-13.77189	-1.04053	0.54306
54	C	-14.04706	0.95867	1.31801
55	C	-13.42742	2.19815	1.61784
56	C	-12.03183	2.34007	1.35059
57	O	-11.38308	3.49038	1.62250
58	H	-11.96115	4.13759	2.04129
59	C	2.43501	1.14278	-1.92898
60	H	2.48867	1.50621	-2.94018
61	N	3.23484	0.16587	-1.46843
62	N	2.98401	-0.09074	-0.18907
63	N	2.01887	0.68667	0.18695
64	C	4.34483	-0.46224	-2.13908
65	H	4.47379	-1.46422	-1.73581
66	H	4.11305	-0.53985	-3.19988
67	C	5.62490	0.34741	-1.96284
68	N	6.72050	-0.25184	-2.42612
69	H	6.64120	-1.19093	-2.79152
70	O	5.61390	1.46742	-1.45659
71	C	8.04590	0.35421	-2.36241
72	H	7.92562	1.41648	-2.58741
73	C	8.66210	0.21520	-0.97245
74	H	8.01295	0.68487	-0.23270
75	H	8.72737	-0.84929	-0.72721
76	C	10.05301	0.83794	-0.94293
77	H	10.49004	0.74235	0.05156
78	H	9.98462	1.90688	-1.16909
79	C	10.96420	0.18916	-1.98651
80	H	11.06438	-0.87488	-1.75438
81	C	10.34624	0.31329	-3.37394
82	H	10.30376	1.37469	-3.64074

83	H	10.98322	-0.18249	-4.10698
84	C	8.94259	-0.28037	-3.41643
85	H	8.99344	-1.35752	-3.22686
86	H	8.50601	-0.14193	-4.40615
87	N	12.29685	0.77135	-2.00667
88	H	12.36348	1.64382	-2.51317
89	C	13.18353	0.64130	-0.96937
90	C	13.06215	-0.31258	0.02527
91	H	12.21364	-0.98252	0.05280
92	C	14.04435	-0.43601	1.02979
93	C	15.16863	0.43395	1.03666
94	C	15.26760	1.41411	0.01124
95	H	16.11858	2.08410	0.01115
96	C	14.31586	1.51531	-0.95166
97	H	14.39837	2.26287	-1.73150
98	C	13.94664	-1.41256	2.05580
99	H	13.09350	-2.07944	2.05882
100	C	14.90612	-1.51717	3.02198
101	H	14.80543	-2.27197	3.78991
102	C	16.02636	-0.64743	3.02956
103	C	16.13907	0.30854	2.04313
104	H	16.99037	0.97823	2.03796
105	C	17.07680	-0.74211	4.07327
106	O	18.02314	0.03183	4.08325
107	C	16.95465	-1.80939	5.12044
108	H	16.91811	-2.79352	4.65138
109	H	16.02577	-1.68146	5.67820
110	H	17.80185	-1.75345	5.79776
111	C	-15.42571	0.80209	1.57703
112	H	-15.88903	-0.14859	1.34588
113	C	-16.16110	1.82851	2.10671
114	H	-17.21717	1.69703	2.30048
115	C	-15.54726	3.06139	2.40026
116	H	-16.13436	3.86896	2.81527
117	C	-14.21144	3.24318	2.16168
118	H	-13.77644	4.20550	2.39792

Table S4. Atomic coordinates for the optimized ground state of **BDP/SNP/ACD** at the SMD(water)/M062X/Def2TZVPP level of theory.

**Energy (Hartree)**	-4002.074318
**Imaginary frequencies**	0

Atom type		Atomic coordinates		
		x	y	z
1	C	1.18667	0.07043	-1.34698
2	C	1.15738	1.45314	-1.53697
3	H	0.23207	1.95258	-1.78976
4	C	2.31136	2.21989	-1.43952
5	H	2.28169	3.28892	-1.59342
6	C	3.47963	1.54498	-1.14326
7	C	3.53248	0.17568	-0.94572
8	C	2.38709	-0.58008	-1.05439
9	H	2.40502	-1.65299	-0.90917
10	N	4.78639	2.06682	-0.98900
11	C	5.64673	1.11992	-0.67936
12	C	4.94306	-0.22546	-0.60650
13	C	5.06175	3.50197	-1.08099
14	H	6.07273	3.63341	-1.45660
15	H	4.38074	3.91073	-1.82467
16	C	4.87346	4.18141	0.26634
17	H	3.85395	4.00692	0.61782
18	H	5.55480	3.72949	0.99131
19	C	5.14169	5.67604	0.15301
20	H	4.45922	6.11501	-0.57720
21	H	6.15824	5.83683	-0.21116
22	C	7.01759	1.40405	-0.40550
23	H	7.31372	2.44262	-0.36301
24	C	5.47054	-1.25684	-1.61984
25	H	5.62923	-0.79800	-2.59537
26	H	4.72426	-2.04356	-1.72778
27	H	6.39803	-1.72224	-1.28841
28	C	4.99626	-0.75623	0.83783
29	H	4.37567	-1.64904	0.90204
30	H	4.61531	-0.01046	1.53624
31	H	6.01695	-1.01507	1.11643
32	C	4.96602	6.36518	1.49531
33	H	5.65424	5.96546	2.24099
34	H	3.94762	6.25552	1.86974
35	S	5.28047	8.11328	1.42124
36	C	-0.04053	-0.77782	-1.46956
37	N	-1.22211	-0.18753	-1.26363
38	H	-1.26773	0.77026	-0.95268
39	O	0.04384	-1.97765	-1.74711

40	C	-2.46423	-0.94191	-1.38230
41	H	-2.50275	-1.40934	-2.36519
42	H	-2.48266	-1.73671	-0.63541
43	C	-3.63924	-0.05134	-1.19798
44	O	4.31957	8.66924	0.46667
45	O	5.07360	8.61387	2.78081
46	O	6.66580	8.26699	0.97179
47	C	7.93279	0.42806	-0.22832
48	H	7.63523	-0.60650	-0.31051
49	C	9.33695	0.63711	0.04951
50	C	9.84829	1.88549	0.50558
51	H	9.16967	2.71793	0.63190
52	C	11.16062	2.02849	0.82566
53	H	11.53868	2.97140	1.19864
54	C	12.06760	0.94539	0.66805
55	C	11.59326	-0.29983	0.18222
56	C	10.20460	-0.43293	-0.08842
57	O	9.71639	-1.61423	-0.56545
58	C	-4.41060	0.58713	-2.13410
59	H	-4.39661	0.60451	-3.20977
60	N	-5.30300	1.28478	-1.41161
61	N	-5.12204	1.08518	-0.10993
62	N	-4.11274	0.28563	0.02897
63	C	-6.42804	2.05285	-1.88479
64	H	-6.63245	2.84685	-1.17116
65	H	-6.16358	2.50038	-2.84175
66	C	-7.65333	1.16932	-2.08653
67	N	-8.80833	1.83049	-2.13058
68	H	-8.80547	2.82254	-1.93678
69	O	-7.54747	-0.04594	-2.24055
70	C	-10.09272	1.17921	-2.36332
71	H	-9.92983	0.40356	-3.11515
72	C	-10.62847	0.52470	-1.09217
73	H	-9.91232	-0.21211	-0.72693
74	H	-10.73132	1.29581	-0.32249
75	C	-11.98261	-0.12313	-1.35710
76	H	-12.36116	-0.59436	-0.44952
77	H	-11.87643	-0.90732	-2.11354
78	C	-12.98658	0.90715	-1.87763
79	H	-13.11935	1.68328	-1.11843
80	C	-12.45152	1.56915	-3.14145
81	H	-12.37484	0.80812	-3.92535
82	H	-13.15604	2.32559	-3.48832
83	C	-11.08396	2.20025	-2.90507
84	H	-11.17664	3.01394	-2.17829

85	H	-10.70303	2.63035	-3.83196
86	N	-14.29046	0.32927	-2.15982
87	H	-14.35846	-0.14181	-3.05159
88	C	-15.11805	-0.16130	-1.18407
89	C	-14.97053	0.12857	0.16072
90	H	-14.14436	0.73173	0.51189
91	C	-15.89511	-0.35695	1.10857
92	C	-16.98874	-1.16246	0.68844
93	C	-17.11424	-1.45776	-0.69697
94	H	-17.94107	-2.07546	-1.02533
95	C	-16.21726	-0.97906	-1.59647
96	H	-16.31897	-1.20596	-2.65084
97	C	-15.76850	-0.06936	2.49324
98	H	-14.93753	0.54155	2.82315
99	C	-16.67274	-0.54828	3.39767
100	H	-16.54974	-0.31175	4.44570
101	C	-17.76532	-1.34910	2.97744
102	C	-17.90419	-1.64260	1.63793
103	H	-18.73365	-2.25411	1.30451
104	C	-18.76016	-1.87640	3.94378
105	O	-19.69331	-2.57147	3.56830
106	C	-18.59951	-1.53910	5.39690
107	H	-18.60636	-0.45695	5.53439
108	H	-17.63909	-1.90571	5.76219
109	H	-19.40697	-1.98965	5.96660
110	C	13.44269	1.08567	0.96057
111	H	13.79729	2.03607	1.33860
112	C	14.30855	0.04498	0.75888
113	H	15.36024	0.16300	0.98291
114	C	13.84067	-1.18136	0.24187
115	H	14.53932	-1.98724	0.06295
116	C	12.51334	-1.35286	-0.04228
117	H	12.16798	-2.28814	-0.46010
118	C	9.76531	-2.76612	0.29833
119	H	10.39661	-3.51797	-0.16615
120	H	10.20752	-2.50263	1.25086
121	C	8.36961	-3.31382	0.46091
122	C	7.64290	-3.05725	1.62192
123	N	6.36005	-3.59846	1.79125
124	B	5.68533	-4.65270	0.81088
125	N	6.54032	-4.56739	-0.51922
126	C	7.83492	-4.05000	-0.59564
127	C	6.25379	-5.14296	-1.68811
128	C	7.35574	-5.01315	-2.55777
129	H	7.39341	-5.38870	-3.56849

130	C	8.36098	-4.34393	-1.89271
131	C	7.95574	-2.27928	2.78484
132	C	6.85721	-2.38130	3.60647
133	H	6.73707	-1.92404	4.57632
134	C	5.90125	-3.20782	2.97977
135	C	4.13156	-4.28699	0.49535
136	H	3.62506	-3.80349	1.32880
137	H	3.55212	-5.18469	0.26596
138	H	4.02524	-3.61558	-0.36065
139	C	5.91232	-6.12751	1.45409
140	H	6.97264	-6.31732	1.65260
141	H	5.56028	-6.91875	0.78599
142	H	5.37951	-6.24946	2.40143
143	C	4.59262	-3.57784	3.58665
144	H	3.81402	-2.87642	3.27828
145	H	4.67965	-3.51663	4.67012
146	H	4.27163	-4.57887	3.31187
147	C	9.17470	-1.49129	3.15173
148	H	8.99429	-0.98910	4.10072
149	H	9.41947	-0.72857	2.41262
150	H	10.05142	-2.12855	3.27677
151	C	4.97690	-5.83230	-2.01986
152	H	4.67200	-6.52150	-1.23421
153	H	5.10452	-6.38807	-2.94640
154	H	4.16816	-5.11419	-2.16061
155	C	9.70153	-4.05420	-2.49037
156	H	10.48939	-4.62644	-1.99663
157	H	9.97037	-3.00143	-2.43026
158	H	9.69216	-4.34440	-3.53959
